# Single-nucleotide variant calling in single-cell sequencing data with Monopogen

**DOI:** 10.1038/s41587-023-01873-x

**Published:** 2023-08-17

**Authors:** Jinzhuang Dou, Yukun Tan, Kian Hong Kock, Jun Wang, Xuesen Cheng, Le Min Tan, Kyung Yeon Han, Chung-Chau Hon, Woong-Yang Park, Jay W. Shin, Haijing Jin, Yujia Wang, Han Chen, Li Ding, Shyam Prabhakar, Nicholas Navin, Rui Chen, Ken Chen

**Affiliations:** 1 Department of Bioinformatics and Computational Biology, The University of Texas MD Anderson Cancer Center, Houston, TX USA; 2 Genome Institute of Singapore (GIS), Agency for Science, Technology and Research (A*STAR), Singapore, Republic of Singapore; 3 Human Genome Sequencing Center, Department of Molecular and Human Genetics, Baylor College of Medicine, Houston, TX USA; 4 Samsung Genome Institute, Samsung Medical Center, Seoul, South Korea; 5 Laboratory for Genome Information Analysis, RIKEN center for Integrative Medical Sciences, Graduate School of Integrated Sciences for Life, Hiroshima University, Higashi-Hiroshima, Japan; 6 Laboratory for Advanced Genomics Circuit, RIKEN Center for Integrative Medical Sciences, Yokohama, Japan; 7 Human Genetics Center, Department of Epidemiology, Human Genetics and Environmental Sciences, School of Public Health, The University of Texas Health Science Center, Houston, TX USA; 8 Center for Precision Health, McWilliams School of Biomedical Informatics, The University of Texas Health Science Center, Houston, TX USA; 9 McDonnell Genome Institute, Washington University School of Medicine, St. Louis, MO USA; 10 Department of Medicine, Washington University School of Medicine, St. Louis, MO USA; 11 Department of Genetics, Washington University School of Medicine, St. Louis, MO USA; 12 Siteman Cancer Institute, Washington University School of Medicine, St. Louis, MO USA; 13 Department of Systems Biology, The University of Texas MD Anderson Cancer Center, Houston, TX USA

**Keywords:** Data integration, Software, Genetic markers, RNA sequencing

## Abstract

Single-cell omics technologies enable molecular characterization of diverse cell types and states, but how the resulting transcriptional and epigenetic profiles depend on the cell’s genetic background remains understudied. We describe Monopogen, a computational tool to detect single-nucleotide variants (SNVs) from single-cell sequencing data. Monopogen leverages linkage disequilibrium from external reference panels to identify germline SNVs and detects putative somatic SNVs using allele cosegregating patterns at the cell population level. It can identify 100 K to 3 M germline SNVs achieving a genotyping accuracy of 95%, together with hundreds of putative somatic SNVs. Monopogen-derived genotypes enable global and local ancestry inference and identification of admixed samples. It identifies variants associated with cardiomyocyte metabolic levels and epigenomic programs. It also improves putative somatic SNV detection that enables clonal lineage tracing in primary human clonal hematopoiesis. Monopogen brings together population genetics, cell lineage tracing and single-cell omics to uncover genetic determinants of cellular processes.

## Abstract (web summary)

Monopogen identifies single-nucleotide variants in single-cell sequencing data.

## Main

Defining the precise cellular contexts in which risk-associated variants affect cellular processes will help to better understand the molecular mechanisms of disease risks and to inform therapeutic strategies. This is important because recent studies have shown that many genetic variants affect tissue traits in a cell-type-specific manner^1,2^. Traditional bulk RNA analysis is usually biased toward abundant cell types defined by a limited set of marker genes^3^.

Single-cell sequencing has enabled comprehensive estimation of cellular composition and acquisition of cell-type-specific molecular profiles^4^, including rare cell types^5^. As opposed to bulk data, single-cell data allow linking genetics to cellular molecular traits such as variability in cellular expressions^6^, cell type abundance^7^ and gene regulatory networks^8^. As such, single-cell analyses in a population-based setting are becoming mainstream^9^.

Although single-cell omics projects are increasingly profiling cell types/states on diverse tissue samples, such as those collected by the ancestry networks of the human cell atlas (HCA)^10^ and human tumor atlas network (HTAN)^11^, the genetic ancestry of the samples and its contribution to cellular molecular traits are largely unexplored. To acquire an accurate genetic profile, it is often necessary to resequence the study samples using bulk whole-genome sequencing (WGS)/whole-exome sequencing, which requires additional sequencing efforts and costs.

A potential cost-effective approach is to call genetic variants directly from single-cell sequencing data, akin to previous studies using low-pass WGS^12,13^ or bulk RNA sequencing^14^. A systematic comparison shows that traditional tools for bulk analysis, such as Samtools^15^ and GATK^16^, detected less than 8% of variants from full-length SMART-seq2 data and considerably less from droplet-based data^17^. Possible reasons for low variant detection are as follows: (1) the single-cell RNA sequencing (scRNA-seq) reads are usually enriched in specific genomic regions, such as 5′ or 3′ end of genes; (2) genes are usually expressed in cell-type/state-specific patterns and thus are highly variable across genome regions, leading to uneven sequencing depth distribution; (3) coverage is likely affected by allelic imbalance inherent in RNA profiles and (4) sequencing reads tend to have many errors due to technological infidelity.

To fill in this gap, we developed Monopogen, a computational framework that enables researchers to detect single-nucleotide variants (SNVs) from a variety of single-cell transcriptomic and epigenomic sequencing data. To achieve sensitive germline SNV detection and accurate genotyping, Monopogen uses high-quality haplotype and linkage disequilibrium (LD) data from an external reference panel to overcome uneven sequencing coverage, allelic dropout and sequencing errors in single-cell sequencing data. To enable accurate somatic SNV calling, Monopogen further conducts LD scoring at the cell population level within each sample, leveraging the expectation that most alleles are identical and in perfect LD with neighboring alleles across the genome, except for those that are somatically altered in a subpopulation of cells. A statistical algorithm that tests against the above expectation, combined with error-suppressing machine learning algorithms, is developed to detect putative somatic SNVs. Monopogen thus brings together population genomics, single-cell genomics and cellular lineage tracing analysis to uncover genetic drivers of cellular processes in ongoing single-cell sequencing studies from various platforms, including scRNA-seq, single-nucleus RNA sequencing (snRNA-seq), scATAC-seq and scDNA-seq^10,11^.

## Results

### Workflow of Monopogen

**Fig. 1:**
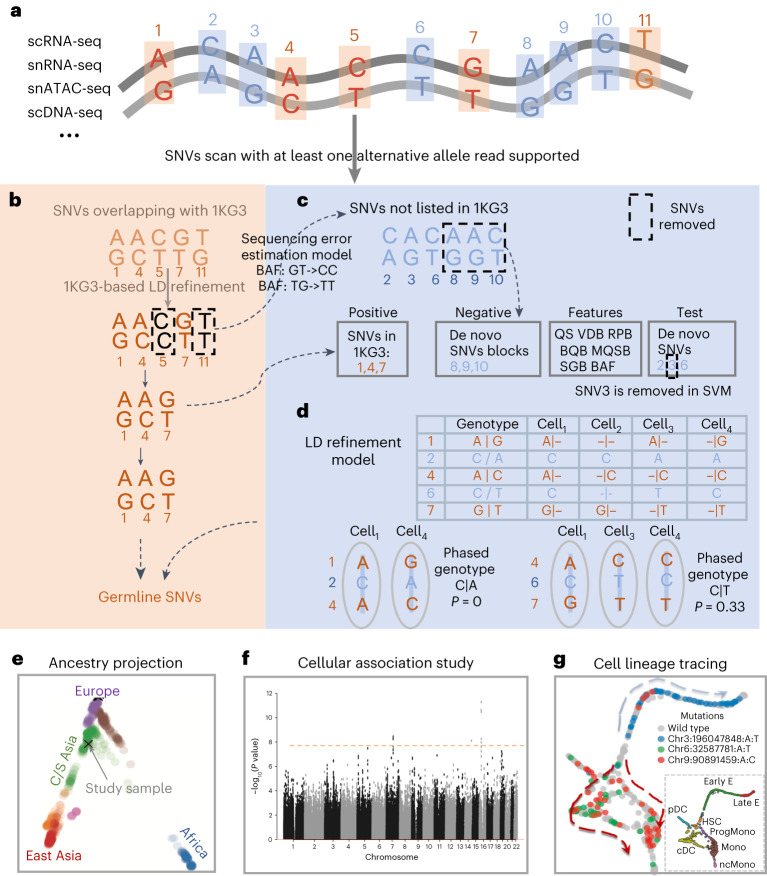
An overview of Monopogen workflow. Monopogen includes germline and putative somatic SNV calling modules. **a**, Monopogen starts from individual bam files produced by single-cell sequencing technologies, including scRNA-seq, snRNA-seq, snATAC-seq and scDNA-seq. Sequencing reads with multiple alignment mismatches (default four) are removed. Putative SNVs are identified sensitively from pooled pileup containing at least one nonreference read. **b**, For SNVs present in the external reference panel (such as 1KG3), genotype likelihoods are further refined based on LD in the reference panel. The loci showing persistent discordance are used to estimate a sequencing error model. **c**, For the remaining loci, we identify putative somatic SNVs by focusing on ones if there is sufficient sequencing depth and alternative allele frequency (calibrated by a sequencing error model). The SVM module is designed to remove low-quality SNVs. The variant calling metrics including the QS for calling, VDB for filtering splice-site artifacts, Mann–Whitney *U* test of RPB, Mann–Whitney *U* test of BQB, Mann–Whitney *U* test of ratio of MQSB, SGB and BAF. The germline SNVs are considered as the positive training sets, while the continuous de novo SNV chunks (>2 SNVs) that do not include any germline SNV are set as the negative sets. The remaining de novo SNVs are considered as the test set. **d**, The alleles observed at a de novo SNV site are statistically phased together with adjacent germline alleles to calculate an LD refinement score that estimates the percentage of cells in which the alleles do not cosegregate with neighboring germline alleles. De novo SNVs with high LD refinement scores are classified as the putative somatic SNVs, and their genotypes at the single cell/cluster level are inferred using Monovar. **e**, Projection of study samples onto the HGDP enables genetic ancestry inference. **f**, Genome-wide association study of cellular quantitative traits can be performed when there is sufficient sample size. **g**, Lineage tracing at single cell or clonal level. QS, quality score; VDB, variant distance bias; RPB, read position bias; BQB, base quality bias; MQSB, mapping quality and strand bias; SGB, segregation-based metric; HGDP, Human Genome Diversity Project.

Monopogen includes germline and putative somatic SNV calling from single-cell sequencing data. It starts from individual bam files of single-cell sequencing data, produced by scRNA-seq, snRNA-seq, single-nucleus assay for transposase-accessible chromatin using sequencing (snATAC-seq), single-cell DNA-seq, etc. (Fig. 1a). Monopogen leverages LD patterns at the human population level to enhance germline SNV detection and LD patterns at the cell population level to enhance putative somatic SNV detection. Sequencing reads with high alignment mismatches (default four mismatches) are removed. Putative SNVs are detected from pooled (across cells) read alignment wherever an alternative allele is found in at least one read. For SNVs that are present in an external haplotype reference panel, such as the 1000 Genomes phase 3 (1KG3) panel, the input genotype likelihoods (GL) estimated by Samtools are further refined by leveraging LD from the reference panel to account for genotyping uncertainty in sparse sequencing data. The loci showing persistent discordance after LD refinement are used to estimate a sequencing error model for de novo SNV calling (Fig. 1b). For the remaining loci satisfying minimal total sequencing depth and alternative allele frequency cutoffs, a support vector machine (SVM) module is designed to distinguish SNVs from sequencing errors (Fig. 1c and Supplementary Fig. 1a, step 2). Briefly, the SVM module uses a series of variant calling metrics as features. The germline SNVs are set as the positive set, and consecutive de novo SNV chunks (>2 SNVs) are set as the negative set. We extend the machinery of LD refinement from the human population level to the cell population level to detect somatic SNVs that are only present in subpopulations of cells. Briefly, for de novo SNVs passing the SVM filtering, we statistically phase the observed alleles with adjacent germline alleles to estimate the degree of LD in the cell population (Fig. 1d and Supplementary Fig. 1a, steps 3–4; Methods). We assume that only two alleles are present in the cell population and examine only the gain of heterozygosity SNVs. We calculate a probabilistic LD refinement score that quantifies the degree of LD, considering widespread sparseness and allelic dropout in single-cell sequencing data (Methods). The LD refinement score ranges from 0 to 0.5. It is closer to 0 for a germline SNV as it has strong LD with the adjacent germline SNVs, that is, sharing the same two haplotypes in all the cells (Supplementary Fig. 1b). The score is greater than 0 for a somatic SNV as the recently gained somatic allele cosegregates with germline alleles in only a subpopulation of cells (Fig. 1d, Supplementary Fig. 1a, step 4, and Supplementary Fig. 1b). SNVs with larger LD refinement scores are classified as putative somatic SNVs. Their genotypes at single cell or cluster level are further inferred using Monovar (Supplementary Fig. 1a, step 5)^18^. The germline SNVs from Fig. 1b can be used for global or local ancestry inference (Fig. 1e) or cellular quantitative trait mapping when the sample size is sufficient (Fig. 1f), and the putative somatic SNVs can be used for lineage tracing at cellular or clonal resolution (Fig. 1g).

Monopogen is implemented in Python, automatically splitting the genome into small chunks (defined by the users), performing variant scan and LD refinement in massive parallelization for individual chunks and merging the results (Supplementary Note).

### Benchmarking of Monopogen performance on germline SNV calling

**Fig. 2:**
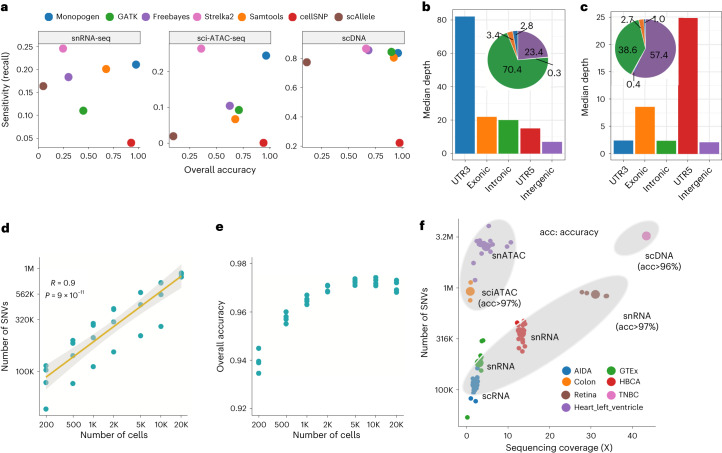
Benchmarking of Monopogen performance in various single-cell sequencing platforms. **a**, Overall accuracy and SNV detection sensitivity (recall) in representative snRNA-seq (*n* = 4), sci-ATAC-seq data (*n* = 2) and scDNA-seq data (*n* = 1) using matched WGS data as the gold standard, comparing Monopogen against Samtools, GATK, FreeBayes, Strelka2, cellSNP and scAllele. The *x* axis denotes the overall accuracy and *y* axis denotes the detection sensitivity (recall). The closer a dot is to the top-right corner, the better the corresponding method has performed. Note, in **a** for Monopogen evaluation, only the SNVs present in the 1KG3 were considered. **b**,**c**, Median sequencing depth of SNVs found from snRNA-seq data (**b**) and sci-ATAC-seq data (**c**) over gene annotations. The pie charts show the percentage of SNVs in each category. **d**, Number of SNVs versus the number of cells in the retina data via downsampling. The *x* and *y* axes are on logarithmic scale. Pearson’s correlations were applied to calculate the *R* and the *P* values. **e**, Overall accuracy versus cell number. **f**. Number of SNVs detected from seven single-cell sequencing datasets. The sequencing coverage was calculated as the $L\times n/(3.2\times {10}^{9})$, where *L* is the read length and *n* is the total number of reads in one sample. Each small dot corresponds to a sample, while each big dot is the mean value of a dataset. All the dots are colored by dataset. The top ellipse covers samples from scATAC-seq data and the bottom ellipse samples from scRNA-seq data.

We used three single-cell sequencing datasets (snRNA-seq from four retina tissue samples, sci-ATAC-seq from two colon tissue samples and scDNA-seq from one triple-negative breast cancer (TNBC) sample) having matched WGS data to evaluate SNV calling performance. In all these samples, the overall accuracy (Methods) of the Monopogen calls was higher than 95% for the germline SNVs present in the 1KG3 panel, 97% for 5/7 of the samples (Fig. 2a and Supplementary Table 1). The high accuracy is largely due to the LD-based genotyping refinement. The overall accuracy without LD-based refinement for bulk-based SNV callers, such as calls from Samtools, GATK, FreeBayes and Strelka2, was less than 73% on snRNA-seq and sci-ATAC-seq (Supplementary Table 2). Further examination shows that over 85% of the genotyping errors from Monopogen misclassified 0/1 as 1/1 (Supplementary Table 1), due partly to allele drop artifacts in the single-cell data.

In the retina snRNA-seq data, Monopogen detected 827–905 K germline SNVs, achieving a recall of 21% (Fig. 2a and Supplementary Table 1). GATK, Samtools and FreeBayes achieved a recall of 11–20% at the expense of lower accuracy (<73%). Although Strelka2 detected ~25% SNVs, the accuracy was lower than 25%. Most (70.4%) SNVs from Monopogen were detected in intronic regions, only less than 7% in exonic regions (Fig. 2b). As expected, sequencing depth was higher in genes than in intergenic regions. Off-target reads appear sufficiently leveraged to derive accurate genotypes through LD-based refinement.

In addition, Monopogen detected ~100 K new SNVs in the retina snRNA-seq data that are not presented in the 1KG3 panel, after performing sequencing depth filtering (>100) and sequencing error model calibration. The overall accuracy of this set is 35% and is 86% for the subset detected in more than 90% of the transcriptomic clusters determined by Seurat^19^ (Supplementary Table 3).

In the colon sci-ATAC-seq data, Monopogen detected 752 K to 1.1 M germline SNVs, achieving a recall of 25%. In contrast, the recall for Samtools, GATK and FreeBayes was less than 12%. Strelka2 detected ~30% SNVs with an accuracy lower than 40%. Most (57.4%) of the SNVs from Monopogen were found in intergenic regions and 38.6% in gene regions (Fig. 2c). We also included two SNV callers cellSNP and scAllele that were designed for single-cell sequencing data. cellSNP had the lowest SNV detection (<5%), and scAllele had the lowest accuracy (<10%) across three benchmarking datasets.

Given single-cell sequencing is highly sparse, sequencing coverage is one of most key factors affecting SNV detection (Supplementary Fig. 2a–c). We evaluated Monopogen’s performance on downsampled retina snRNA-seq data containing random subsets of 200–20,000 cells (~29.4 K reads per cell; Supplementary Table 1). We observed a linear relationship between the number of SNVs and cell numbers in a logarithmic scale (Fig. 2d; Pearson correlation coefficient is 0.9). Monopogen detected ~100 K SNVs from only 200 cells and 500 K SNVs from 1,000 cells (Fig. 2d). Despite downsampling, the overall accuracy of Monopogen remained robust to cell number and was always higher than 94% (Fig. 2e). The downsampling sequencing coverage scheme showed a similar pattern to the downsampling cell scheme (Supplementary Fig. 2d,e). The performance of Monopogen was robust to sequencing depth and errors. The overall accuracy had only slight decreases when sequencing error rates were less than 2.5%. Even at an exceedingly high sequencing error rate of 5%, Monopogen still achieved ~85% genotyping accuracy (Supplementary Fig. 2e), demonstrating the efficiency of LD-based genotyping refinement on challenging scenarios.

We further evaluated Monopogen in four other cohorts, which are as follows: human breast cell atlas (HBCA; 20 donor samples), peripheral blood mononuclear cells from Asian Immune Diversity Atlas (AIDA; 20 donor samples), genotype-tissue expression project (GTEx; seven donor samples) and human heart left ventricle atlas (65 samples). These datasets have a variety of cell numbers, number of reads per cell and read length (Supplementary Table 4). To make a fair comparison across datasets, we investigated the relationship between sequencing coverage and number of SNVs. As expected, Monopogen detected more SNVs from single-cell epigenomics sequencing data than from single-cell transcriptomics sequencing data (Fig. 2f). Although these samples do not have matched WGS profiles, there are 54 human left ventricle samples having paired scRNA-seq and scATAC-seq. The genotyping concordance between the two modalities was also as high as 97% (Supplementary Table 5 and Supplementary Fig. 5a), further demonstrating the robustness of Monopogen SNVs calling on various sequencing platforms.

### Accurate global and local ancestry inference from single-cell sequencing data

**Fig. 3:**
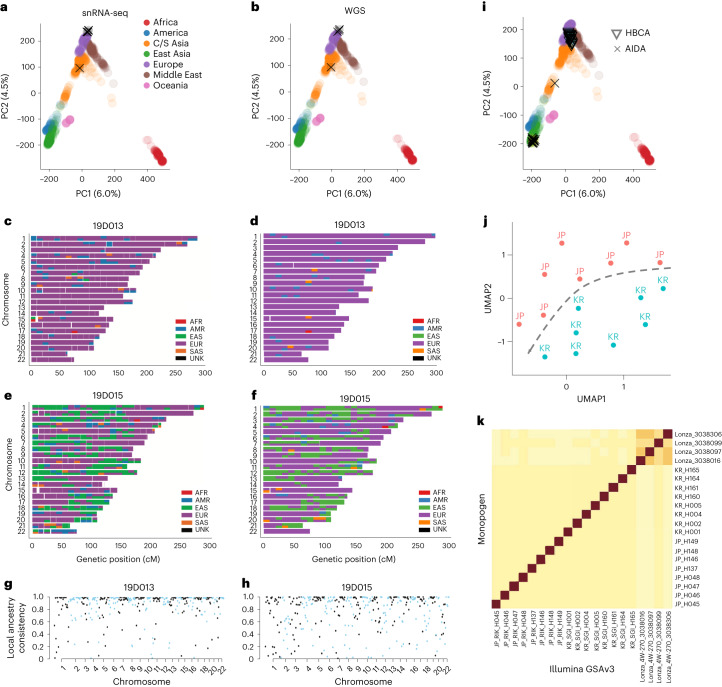
Global and local ancestry inference using single-cell genotypes derived by Monopogen. **a**,**b**, Genetic ancestry of the four retina samples using Monopogen genotypes derived from snRNA-seq data (**a**) and genotypes from matched WGS data (**b**). Colored dots represent individuals in the HGDP reference panel, and black crosses represent the retina samples. The variance explained by PC1 and PC2 from the HGDP panel was labeled. **c**,**d**. Local ancestry inference of a European sample 19D013 using genotypes from the snRNA-seq (**c**) and the WGS (**d**) data. The 3,202 phased genotypes from 1KG3 were used as the reference for local ancestry inference. Colors in each chromosome denote the inferred source ancestry with a bin size of 1 centimorgan (cM). **e**,**f**, Local ancestry results from an admixed sample 19D015. **g**,**h**, Local ancestry inference accuracy for 19D013 (**g**, overall score: 0.96) and 19D015 (**h**, overall score: 0.90). Each dot denotes the ancestry accuracy for each segment (1 cM). **i**, PCA-projection analysis shows the ancestry of samples in the AIDA and the HBCA cohorts. **j**, UMAP of Korean and Japanese samples in the AIDA using genotypes called Monopogen. The UMAP was constructed based on the top five PCs of Korean and Japanese genotypes (on 584,164 SNVs). **k**, Concordance between Illumina GSAv3 genotyping array data and Monopogen calls across the AIDA samples. Darker colors denote a higher level of concordance between two data modalities. Calculation of the concordance scores is detailed in Methods.

We performed genetic ancestry inference using genotypes called from Monopogen. We projected the Monopogen-called snRNA-seq genotypes and the matched WGS genotypes of the four retina samples, respectively, onto a map, consisting of source samples with East Asia, America, Middle East, Europe, Oceania, Africa and Central/South Asia in the Human Genome Diversity Project (HGDP)^20^. We found that the PC coordinates were highly consistent between the WGS genotypes and the single-cell genotypes called by Monopogen (Fig. 3a,b). The mapping results were consistent with self-reported ethnicities for all the samples, including three Europeans and a self-reported Hispanic sample. We further performed local ancestry inference using RFMix^21^. On all the samples, the chromosomal painting results based on single-cell data (Fig. 3c–f and Supplementary Fig. 3) appeared highly consistent with self-reported ethnicities and with those obtained from the WGS data. For example, the source consistency across genomic bins was as high as 0.96 for one of the European samples (19D013; Fig. 3g) and 0.90 for the Hispanic sample (19D015; Fig. 3h). We did observe some genomic bins showing discrepant sources, due largely to sparseness of single-cell-derived SNVs in those regions. The global ancestry inference results remained largely unchanged when downsampling the data to only 200 cells (~29.4 K reads per cell; Supplementary Fig. 4).

We also performed projection analysis on another 40 samples in the HBCA and the AIDA cohorts that do not have matched WGS data. Again, the global ancestry inferred from single-cell sequencing was consistent with self-reported ethnicities except for one putative admixed sample in the AIDA cohort (Fig. 3i). In the AIDA cohort, it is difficult to separate Japanese and Korean samples by PCA-projecting them onto the HGDP panel. However, these two populations can be well separated by performing independent UMAP analysis using Monopogen-derived genotypes (Fig. 3j). Furthermore, Monopogen shows consistent performance in identifying donor-specific SNVs in the AIDA samples, based on the concordance of Monopogen-derived genotypes and Illumina GSAv3 genotypes (Fig. 3k), demonstrating the possibility of distinguishing individuals from the same ancestry. This indicates that the LD-based genotyping refinement from the commonly used 1KG3 panel did not over-correct genotypes on subpopulation or individual levels, despite sparse sequencing coverage.

### Genome-wide association study of cellular quantitative traits

To demonstrate the utilization of Monopogen in establishing the link between genetic variants and cellular quantitative traits in a cell-type or cell-state-specific manner, we characterize the genetic contribution to metabolic processes (such as ATP production) and epigenetic programs in healthy cardiomyocytes. These relationships are usually disguised by previous bulk-based data analysis.

As a demonstration, we collected snRNA-seq and snATAC-seq data of ~4 M cells generated from a human heart left ventricle tissue samples of 65 donors, 54 of which have data from both modalities. Around 791 K SNVs in snRNA-seq and 2.59 M SNVs in snATAC-seq were identified from Monopogen (Supplementary Table 5 and Supplementary Fig. 5a). The variant calling consistency between two modalities was as high as 97% at overlapping loci (Supplementary Fig. 5b,c). Variant calls were further merged for samples of paired modalities.

Ancestry admixture analysis using inferred genotypes shows that this cohort contains samples with diverse ancestry, which are as follows: European (71.1%), Asian (10.2%) and African (8.5%). Six samples appeared admixed (Supplementary Fig. 6a).

**Fig. 4:**
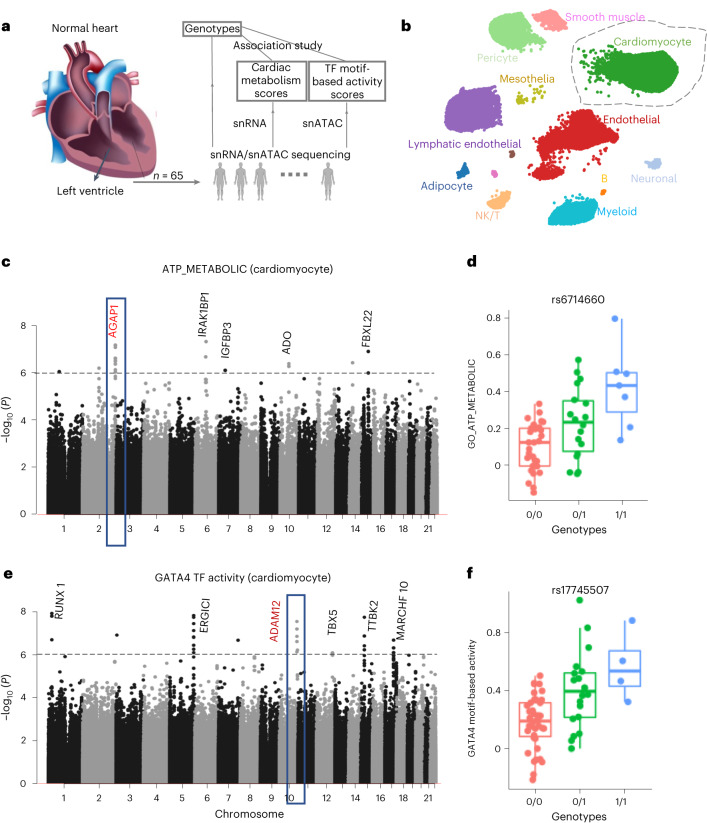
Genetic association study of cardiomyocyte molecular traits using snRNA-seq and snATAC-seq data from heart left ventricle tissues. **a**, Analysis workflow. Details can be seen in Methods. **b**, A UMAP of snRNA-seq cells colored by cell types annotated using Azimuth heart database. **c**, Manhattan plot showing association of Monopogen SNVs with pathway scores of ATP_METABOLIC in cardiomyocytes. The gray line denotes the *P* value threshold of 10^−6^. Genes closest to the top-scoring loci are labeled. **d**, Boxplot shows the difference of ATP_METABOLIC scores across the three genotypes of rs6714660 (one of the leading variants in *AGAP1*). **e**, Manhattan plot showing the association of SNVs with the *GATA4* motif-based transcription factor activity level in cardiomyocytes. The gray line denotes the *P* value threshold 10^−6^. **f**, Boxplot shows the difference in *GATA4* activity level across the three genotypes of rs17745507 (one of the leading variants in *ADAM12*). For each box in **d**,**f**, the centerline defines the median, the height of the box is given by the interquartile range (IQR) and the whiskers are given by 1.5× IQR. All samples (*n* = 54) are given as points.

To explore the cardiac metabolism process, we extracted cardiomyocyte cells from each sample by annotating cells using the human heart Azimuth database (Fig. 4b and Supplementary Fig. 6b). Using pathway expression level as a proxy for ATP metabolism level, we derived cardiac ATP metabolism level by aggregating the expression levels of 216 genes in GO_ATP_METBOLIC pathway (Methods). We performed association analysis using the GCTA tool^22^, including the top five ancestry PCs as covariates. *P* value of 10^−5^ was used as the threshold to identify potential associations due to the small sample size. The inflation factor of the Quantile–Quantile plot was close to 1 (0.983; Supplementary Fig. 7a). A total of 250 variants were associated with cardiac ATP metabolism score (*P* < 10^−5^), which can be further binned into 42 gene regions (Supplementary Table 6), including five genes (at least two variants supported) with *P* value < 10^−6^ (Fig. 4c). Among genes in the regions, *IGFBP3* and *FBXL22* are well known to affect adult cardiac progenitor cells^23^ or cardiac contractile function^24^. *ADO* functions as an oxygen sensor involved in N-degron pathways^25^. These associations further confirm the tight coupling of ATP production and myocardial contraction, which is essential for normal cardiac function^26^. *AGAP1*, indicated by its tag SNV (rs6714660; Fig. 4d), is involved in cardiac ATP production in the Krebs cycle^27^.

We also derived transcription factor (TF) activity scores from the snATAC-seq data (Methods). We then scanned for genetic variants associated with the activity level of *GATA4*, one of the most important TFs highly activated in cardiomyocytes at various developmental stages. The inflation factor of Quantile–Quantile plot was close to 1 (0.984; Supplementary Fig. 7b). A total of 257 variants were identified (*P* < 10^−5^), which can be further binned into 42 gene regions (Supplementary Table 7), six of which (at least two variants supported) with *P* < 10^−6^ (Fig. 4e). Among the genes in the regions, *TBX5–GATA4* and *RUNX1*–*GATA2* complexes are well known for their interdependence in coordinating cardiogenesis^28–31^. *ADAM1*2, indicated by its tag SNV (rs17745507; Fig. 4f), is known to have a key role in cardiac hypertrophy by blocking the shedding of heparin-binding epidermal growth factor^32^. These results indicate a potential association between *GATA4* and cardiac hypertrophy through the mediation of *ADAM12*. Also identified were some variants (*P* < 10^−5^), located in the zinc-finger family genes, such as *ZNF595* and *ZNF750*, that act as cofactors with the zinc-finger TF *GATA4* (Supplementary Table 7).

In summary, we were able to reveal potential genetic determinants of cardiac health via metabolic and epigenomic trait mapping of cardiomyocytes, despite the relatively small sample size. Associations identified in this fashion may lead to a better understanding of the pathogenicity of noncoding variants in a cell-type-aware manner.

### Putative somatic SNV detection on single-cell sequencing

To evaluate the somatic SNV detection module of Monopogen, we examined 1,534 cells from sample of one patient with TNBC sequenced using a single-cell DNA-seq platform^33^. From the matched normal and tumor bulk WGS data of around 87× coverage each, we identified a total of ~3.5 M germline SNVs and 19,766 somatic SNVs (Methods). We classified new SNVs detected by Monopogen into the following three categories: somatic, germline and unknown in the bulk sample (Methods).

To conduct effective somatic SNV detection, we first examined the rational of applying two-locus and three-locus LD refinement models (Methods) using germline SNVs that had phased genotypes at the cell population level. The two-locus model showed low level of LD refinement (<0.01) when the distance between two adjacent loci was less than 100 bp, which indicates physical phasing within the length of the reads. Genotype correlation between two adjacent loci decreased substantially when distance increases over 100 bp. Unlike the pattern in two-locus model, the three-locus model showed a gradual increase of LD refinement score with increased haplotype length. There are over 70% of cosegregated alleles when the length of haplotypes is less than 5 kb, providing rich information for phasing germline SNVs that do not exist in the 1KG3 panel. This pattern was consistent across all the chromosomes (Supplementary Figs. 8–10).

**Fig. 5:**
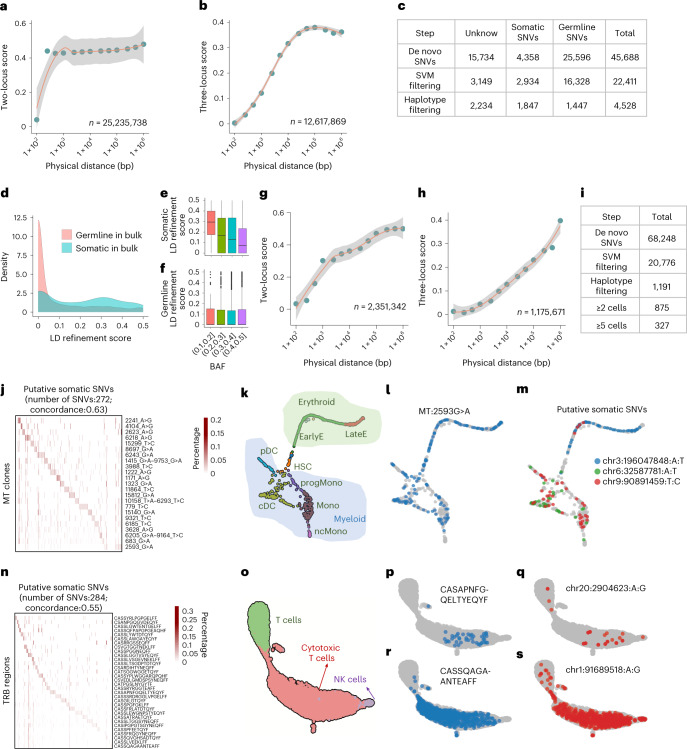
Somatic SNV detection in single-cell sequencing. **a**,**b**, LD refinement scores on germline SNVs from the TNBC single-cell DNA data. It is shown with two-locus model in **a** and three-locus model in **b**. **c**, Evaluation of de novo SNVs from Monopogen by comparison with categories defined in matched bulk DNA sample (Methods). **d**, Distribution of LD refinement scores for de novo SNVs that are classified as germline and somatic SNVs from the bulk sample. **e**,**f**, Boxplot displaying the relationship between LD refinement score and BAF, with SNVs classified as somatic (**e**, *n* = 339) and germline SNVs (**f**, *n* = 2,425). The centerline defines the median, the height of the box is given by the interquartile range (IQR), the whiskers are given by 1.5× IQR and outliers are given as points beyond the minimum or maximum whisker. **g**,**h**, LD refinement scores on germline SNVs from the bone-marrow sample measured in single-cell RNA data. It is shown with two-locus model in **g** and three-locus model in **h**. In **a**, **b**, **g** and **h**, the length of haplotypes is grouped into 13 bins (Methods). The x axis is in logarithmic scale. The y axis shows the mean value of LD refinement score within each bin together with the 95% confidence interval. The total number of haplotypes used for evaluation is labeled at the right-bottom of each panel. **i**, Number of SNVs detected in each step from Monopogen. **j**, Heatmap displaying the detected percentage of putative somatic SNVs in each mtDNA clone (the sum of each row is 1). **k**, UMAPs displaying the cell types annotated in myeloid and erythroid lineages. **l**,**m**. UMAPs displaying the mutated cell distribution for mtDNA variant 2593G:A (**l**) and three selected putative somatic SNVs from scRNA-seq (**m**). **n**, Heatmap displaying the detected percentage of putative somatic SNVs in each TRB clone. **o**–**s**. UMAPs displaying the cell types annotated in T/NK cell lineages (**o**), the mutated cell distribution for TRB region CASAPNFGQELTYEQYF (**p**) and the putative somatic SNV chr20:2904623A:G (**q**), the mutated cell distribution for TRB region CASSQAGAANTEAFF (**r**) and the somatic SNV chr1:91689518A:G (**s**).

Initially, Monopogen identified 45,668 de novo SNVs, among which only 9.5% were classified as somatic, 56.0% germline and the remaining unknown. This highlighted the challenge of somatic SNVs detection from pooled single-cell profiles without using external information. The SVM module substantially reduced the number of unknown SNVs by 90%, while keeping 67.3% of the somatic SNVs and 63.8% of the germline SNVs (Fig. 5a), demonstrating the efficacy of the SVM module on distinguishing SNVs from sequencing errors. This could also be confirmed by examining the feature distribution difference between the positive and the negative labels (Supplementary Fig. 8)

The LD refinement module further removed 91% of the germline SNVs, leading to a total of 1,847 somatic SNVs and 1,447 germline SNVs that are validated by bulk WGS, in addition to 2,234 unknowns in the final de novo SNV call set (Fig. 5c). As expected, LD refinement score distribution for germline SNVs were skewed toward 0 (Fig. 5d). A fraction of somatic SNVs also showed score closing to 0, partly due to the confounding B-allele frequency (BAF) effect (Fig. 5e). Somatic and germline SNVs become inseparable when BAF is close to 0.5. Among the putative somatic SNVs detected (Supplementary Table 8), there were 11 known oncogenes and 12 tumor suppressors. The unknown SNVs from Monopogen may contain low-abundance somatic SNVs that were missed by matched bulk sequencing.

We next evaluated the somatic SNV detection module on 9,346 cells obtained from a bone-marrow sample with clonal hematopoiesis^34^. The cells were profiled using 10× single-cell sequencing combined with mitochondrial transcriptome enrichment (that is, MAESTER technology), leading to joint profiling of gene expressions and mtDNA mutations from the same cells. We also first examined the rational of the two-locus and three-locus LD refinement models from scRNA-seq profiles (Fig. 5g,h and Supplementary Figs. 12 and 13). Different from the single-cell DNA-seq data, the score remained low even though the distance between two adjacent loci was longer than 10 kb, which can be explained by allelic imprinting (or allelic expression) in the transcriptomes. The three-locus LD refinement score showed a similar gradual increase with increased distance, with around 90% of cosegregated alleles when haplotype length is 10 kb. The germline LD refinement patterns examined in both single-cell RNA and single-cell DNA data proved the possibility of capturing both short-distance (within physical reads) and long-distance molecular linkage in single-cell populations even under sparse short-read sequencing. Similarly, feature distributions between the positive and the negative labels were different (Supplementary Fig. 11), enabling SVM classification.

Joint profiling of mtDNA and transcriptomics provided an opportunity to validate the somatic SNVs via comparison of clonal architecture inferred orthogonally from mtDNA variants. We focused on 1,049 cells with both putative somatic SNVs and mtDNA variants detected. There were 391 putative somatic SNVs detected in at least two cells, and 69.6% (272/391) of them were significantly (*P* < 0.01, Wilcox test) enriched in at least one mtDNA clone (Fig. 5j), with around 12 somatic SNVs in each mtDNA clone. The average cellular concordance between the matched somatic SNV clones and mtDNA clones was 0.63 (Methods). These somatic SNVs allowed finer delineation of the clonal architecture. For example, the most variable mtDNA variant 2593G>A was observed in most of the cell types in both myeloid and erythroid lineages (Fig. 5k,l and Supplementary Fig. 14). However, somatic SNVs such as chr3:196,047,84A:T appeared predominantly in erythroid lineage, while chr9:90891459T:C and chr6: 32,587,781A:T predominantly in myeloid lineage (Fig. 5m and Supplementary Fig. 14).

Joint profiling of T-cell antigen receptor (TCR) variable region and transcriptomics also provided an opportunity to validate somatic SNVs. We noted that 60.3% (284/471) of somatic SNVs were enriched in TRB regions and 52.7% (126/239) in TRA regions (Fig. 5n, Supplementary Fig. 15c), with average cellular concordance of 0.55 and 0.54 for the TRB and the TRA regions, respectively. In T cells and cytotoxic T lymphocytes, there are somatic SNVs localized in subregions of a cell type in the transcriptomic UMAPs (Fig. 5q,c). For example, chr20:2904623A:G clone was detected in the bottom of the cytotoxic T-cell cluster (similar pattern with TRB clone CASAPNFGQELTYEQYF in Fig. 5p and Supplementary Fig. 15b). Some mutations (for example, chr1:91689518A:G) spanned across all the T cells (similar pattern with TRB clone CASSQAGAANTEAFF in Fig. 5r and Supplementary Fig. 15b), indicating these putative somatic SNVs may represent multiple T-cell clonotypes that have occurred from multipotent hematopoietic stem cells.

## Discussion

In this study, we developed Monopogen, a computational tool enabling researchers to identify SNVs at high accuracy from sparse single-cell transcriptomic and epigenomic sequencing data. Single-cell sequencing technologies, like other targeted sequencing technologies^13,35,36^, can generate reads that map outside of the target regions, which has become a rich, under-used resource for genomic variant discovery. By leveraging these reads, in conjunction with the known LD patterns in major human populations, Monopogen identified around 100 K to 1 M SNVs in 10X Chromium single-cell or nucleus RNA-seq data, and 1–2.5 M SNVs in single-cell ATAC-seq data at genotyping accuracies higher than 0.95. We found through downsampling experiments that Monopogen can be applied in most single-cell sequencing datasets, including those with low (~200) cell numbers. Although not evaluated in this work, there should be no barrier to apply Monopogen on data produced by other single-cell sequencing platforms such as the full-length smart-seq^37^. With SNVs called by Monopogen, global and local ancestry inference can be reliably performed in studies that have only single-cell sequencing but not bulk sequencing or array-based genotyping data, which greatly increases the chance of discovering genetic factors underlying diverse cellular quantitative traits and disease. In addition, leveraging the power of having phased haplotypes from germline SNVs, the LD refinement models applied at cell population level enabled us to substantially increase the accuracy of somatic SNV detection in sparse, short-read, single-cell sequencing data.

Health disparity is a substantial socioeconomic challenge. Ongoing large-scale single-cell studies (such as HCA and the CZI genetic ancestry network) are aiming at creating a genetically unbiased reference and avoiding the Eurocentric biases in previous human genetic studies^38^. Our study has clearly shown that single-cell sequencing data can potentially be used as a resource to not only determine the genetic ancestry of study samples but also expand the reference to further delineate human populations. For example, we found a clear separation of Japanese and Korean samples in the AIDA cohort based on variants and genotypes determined from single-cell data by Monopogen. Moreover, although our analysis and assessment were based on publicly available reference population databases such as 1KG3, we expect that the power of variant calling and ancestry inference will become greater when using local population panels^39,40^ or proprietary databases with larger population size and greater diversity.

Monopogen adds a genomic modality to current single-cell transcriptomic and epigenomic assays^9,41,42^, which makes it possible to use these assays for functional genetics investigations. For example, we identified SNVs that are associated with the metabolism and epigenetic regulation of cardiomyocytes in heart samples. Many similar analyses can be performed, for example, identifying genetic determinants of cancer immune response using pan-cancer single-cell T-cell atlas data^43^.

Although the single-cell sequencing data is quite sparse, the LD-refinement models enable us to quantify if neighboring SNVs cosegregate in the entire population or only a subpopulation of cells, due to their colocalization on a DNA haplotype or RNA transcript. Phasing genotype profiles at the cell population level opens an opportunity to unravel the clonal affiliations of somatic SNVs that are buried in bulk-seq data. The current two and three loci LD refinement models can be further extended to include multiple loci, when sequencing dropout issues are alleviated, or the sequencing reads become longer in the future. We have shown that the combination of single-cell transcriptomics with somatic SNVs detected by Monopogen can depict finer clonal architecture in a bone-marrow sample undergone clonal hematopoiesis, which may facilitate similar investigations, such as resolving clonal lineage in cancer evolution studies^18,44,45^.

Our study has several limitations. Although Monopogen can potentially detect putative somatic SNVs, it is challenging to separate germline from truncal somatic SNVs whose BAFs are close to 0.5. However, those SNVs can be easily detected via bulk sequencing. In the human heart left ventricle analysis, we demonstrated the utilization of Monopogen-called genotypes to identify associations of ATP metabolism and *GATA4* activity levels in one cell type, cardiomyocytes. In the context of discovery, such analysis can be extended to other cell types and cellular quantitative traits of interest that could be objectively measured. However, such association analysis should be guided by strong prior knowledge to reduce the burden of multiple hypothesis testing.

In summary, we developed a computational tool Monopogen to maximize the genetic information from available single-cell sequencing data, which can lead to immediate benefits on genetic ancestry mapping, association analysis using current large-scale single-cell atlas data^10,11^ and somatic clonal lineage delineation^45^. In the long term, with the increasing generation of sparse single-cell sequencing data and expansion of data modalities, our work will become increasingly relevant for assessing the effects of genetic ancestry and discovering genetic mechanisms underlying complex traits in human populations and diseases.

## Methods

### Monopogen workflow

#### Reads filtering

Monopogen starts from individual bam files of single-cell sequencing data. Reads with high alignment mismatches (default four mismatches) and lower mapping quality (default 20) are removed.

#### SNV discovering

We first scan the putative SNVs in a sensitivity way. Any loci are detected from pooled (across cells) read alignment from one sample wherever an alternative allele is found in at least one read. For each candidate SNV locus *m* with observed sequencing data information *d*, we record its genotype likelihoods (GL) that incorporate errors from base calling and alignment as

1 $${\mathrm{GL}}\left(m{{|}}d\right)=\left\{{\mathrm{GL}}\left(g=i|d\right),i\in 0,1,2\right\},$$

where *g* = 0 denotes homozygous reference allele, *g* = 1 denotes heterozygous and *g* = 2 denotes homozygous alternative allele. Calculation of GL(*g*│*d*) is performed using Samtools mpileup tool^15^.

#### Germline variant calling refinement

Given that scRNA-seq data has high genotyping uncertainties and is quite sparse, we leverage the LD from the 1KG3 database to further refine the GL, including 3,202 samples with a total of ~80 M phased SNVs after quality control. We focus only on putative SNVs existing in both the 1KG3 panel and the single-cell sequencing data. Denotes *H* the set of reference haplotypes ($\left|H\right|=\mathrm{6,404}$). The Beagle hidden Markov model^46,47^ is used to identify the target haplotype of SNV *m* with its adjacent loci, including (1) definition of state space; (2) initial probabilities, (3) transmission probabilities and (4) emission probabilities. Equation (1) is further updated as the genotype probabilities conditioning on the haplotypes in the reference panel as

2 $${\mathrm{GP}}\left(m{{|}}H,d\right)=\left\{P\left(g=i|d,H\right),i\in 0,1,2\right\}.$$

#### Sequencing error modeling

For each locus *m*, we calculate the observed genotype as the one with the highest posterior probability from Eqs. (1) and (2), respectively. Denote

$${G}_{m|d}=\mathop{{{\arg }}\,{{\max }}}\limits_{i}\mathrm{GL}(\,g=i|d),$$

and

$${G}_{m|H,d}=\mathop{{{\arg }}\,{{\max }}}\limits_{i}\mathrm{GP}(g=i|d,H).$$

The final genotype of locus *m* is set as ${G}_{{m|H},d}$ if ${G}_{{m|H},d}={G}_{{m|d}}$. The heterozygous loci that are imputed to homozygotes are considered as sequencing errors (that is, ${G}_{{m|H},d}=0$ and ${G}_{{m|d}}=\mathrm{1,2}$). We classify this discordance into 12 categories:

$$\begin{array}{l}C=\left\{\right.{\mathrm{AT}}\to {\mathrm{AA}},{\mathrm{AT}}\to {\mathrm{TT}},{\mathrm{CT}}\to {\mathrm{CC}},{\mathrm{CT}}\to {\mathrm{TT}},{\mathrm{GT}}\to {\mathrm{GG}},{\mathrm{GT}}\to {\mathrm{TT}},{\mathrm{AC}}\to \\{\mathrm{AA}},{\mathrm{AC}}\to {\mathrm{CC}},{\mathrm{AG}}\to {\mathrm{AA}},{\mathrm{AG}}\to {\mathrm{GG}},{\mathrm{CG}}\to {\mathrm{CC}},{\mathrm{CG}}\to {\mathrm{GG}}\left\}\right.\end{array}$$

The median BAF across all inconsistent loci in each category *c* is denoted as BAF_*c*_. This is considered the threshold to separate the sequencing error from the true heterozygous. SNVs with ${G}_{{m|H},d}={G}_{{m|d}}$ are retained as the germline SNVs (that is, SNVs). Others are only used to build the sequencing error model and are not included in the final genotyping call set.

#### De novo SNV scanning

For putative SNVs absent in the 1KG3, we implement the following two filters: (1) the total sequencing depth filtering (default 100); and (2) BAF less than the threshold from the above sequencing error model. For example, one putative SNV genotyped as *A*/*T* with its BAF lower than ${{\max }}\,\{\mathrm{BA{F}_{AT\to AA},BA{F}_{AT\to TT}}\}$ is removed due to difficulties in separating true heterozygotes from sequencing errors.

#### Putative somatic SNV calling

The somatic SNVs calling includes the following two major modules: (1) removing low-quality SNVs using an SVM and (2) distinguishing somatic from germline SNVs using LD refinement models at the cell population level.

##### Remove low-quality SNVs using SVM

In the SVM module, all detected germline SNVs overlapped with 1KG3 are considered as the positive set. We define de novo SNVs found consecutively (default >2 SNVs) in genomic chunks that do not contain any germline SNV as the negative set. This is because the chance of only detecting multiple somatic SNVs in one region without any germline SNVs is typically low due to the low average somatic mutation rate in most datasets. SNVs calling quality metrics including quality score for calling, variant distance bias for filtering splice-site artifacts, Mann–Whitney *U* test of read position bias, Mann–Whitney *U* test of base quality bias, Mann–Whitney *U* test of ratio of mapping quality and strand bias, segregation-based metric and BAF are selected as features. The model is trained using the svm function implemented in R package e1071. The de novo SNVs with a predicted probability of positive labels less than 0.5 are set as sequencing errors and excluded from downstream analysis.

##### Estimate LD refinement score from germline SNVs

The de novo SNVs passing the SVM filtering are further interrogated using the LD refinement models. The LD refinement models assume that only two alleles are present in the cell population. We first estimate the LD refinement scores on germline SNVs that quantify the degree of their LD, taking into consideration widespread sparseness and allelic dropout in single-cell sequencing data. We then implement germline LD patterns to statistically phase the observed alleles of de novo SNVs in the cell population.

We assume that the germline SNV block includes *n*_*m*_ SNVs with genotype vector being $\left\{{G}_{1},{G}_{2},\cdots ,{G}_{{n}_{m}}\right\}$. Denote ${G}_{i}={A}_{i}^{1}|{A}_{i}^{2}$, where ∙|∙ represents the phased genotype. The cell level genotype matrix *G* on these germline SNVs can be represented as

$${{G}}={\left[\begin{array}{cc}\begin{array}{cc}{c}_{11}^{1}{{\Big|}}{c}_{11}^{2} & {c}_{12}^{1}{{\Big|}}{c}_{12}^{2}\\ {c}_{21}^{1}{{\Big|}}{c}_{21}^{2} & {c}_{22}^{1}{{\Big|}}{c}_{22}^{2}\end{array} & \begin{array}{cc}\cdots & {c}_{1C}^{1}{{\Big|}}{c}_{1C}^{2}\\ \cdots & {c}_{2C}^{1}{{\Big|}}{c}_{2C}^{2}\end{array}\\ \begin{array}{cc}\vdots & \vdots \\ {c}_{{n}_{m}1}^{1}{{\Big|}}{c}_{{n}_{m}1}^{2} & {c}_{{n}_{m}2}^{1}{{\Big|}}{c}_{{n}_{m}2}^{2}\end{array} & \begin{array}{cc}\vdots & \vdots \\ \cdots & {c}_{{n}_{m}C}^{1}{{\Big|}}{c}_{{n}_{m}C}^{2}\end{array}\end{array}\right]}_{{n}_{m}\times C}$$

where *n*_*m*_ is the number of germline SNVs and *C* is the number of cells. ${c}_{{ij}}^{1}$ and ${c}_{{ij}}^{2}$ denote the number of reads supporting allele ${A}_{i}^{1}$ and ${A}_{i}^{2}$ in cell *j*, respectively. If no reads are detected in allele ${A}_{i}^{h}\,(h=\mathrm{1,2}),\,{c}_{{ij}}^{h}$ is set to 0. It is noted that *G* is quite sparse and the majority of its elements are zero (that is, ${c}_{{ij}}^{1}=0$ and ${c}_{{ij}}^{2}=0$). Even for an element with reads detected, rarely can both alleles be captured (one example can be seen in Supplementary Fig. 1a, step 3).

Due to the sparsity of single-cell data, not all adjacent germline SNVs are informative for LD refinement. Here we first define a two-locus neighborhood index in cell *j* to identify informative germline SNV pairs as

3 $${{\mathrm{Neighb}}}_{2}\left(k,i,j\right)=\left\{\begin{array}{ll}1,{if}\,{c}_{{kj}}^{1}{+c}_{{kj}}^{2} > 0,\,{c}_{{ij}}^{1}{+c}_{{ij}}^{2} > 0,\,{\rm{and}} & \,{c}_{{lj}}^{1}+{c}_{{lj}}^{2}=0\,{\rm{for}}\,k < l < i\\ 0, \, {\mathrm{others}}\end{array}.\right.$$

Illustration of two-locus neighborhood index can be seen in Supplementary Fig. 1b. Denote ${{\mathscr{H}}}_{2}$ as the set including all two-locus neighborhoods, we have

$${\mathbf{\mathscr{H}}}_{2}=\left\{\left({c}_{{kj}}^{1}\left|{c}_{{kj}}^{2},\,{c}_{{ij}}^{1}\right|{c}_{{ij}}^{2}\right)\,{{\mathrm{s}}.{\mathrm{t}}.}\,{{\mathrm{Neighb}}}_{2}\left(k,i,j\right)=1,1\le k < i\le {n}_{m},1\le j\le C\right\}$$

We next group elements in ${{\mathscr{H}}}_{2}$ based on the distance of SNVs as

$${\boldsymbol{\mathscr{H}}}_{2}^{d}=\left\{\left({c}_{{kj}}^{1}\left|{c}_{{kj}}^{2},{c}_{{ij}}^{1}\right|{c}_{{ij}}^{2}\right)\,\mathrm{s.t.}\,\left({c}_{{kj}}^{1}\left|{c}_{{kj}}^{2},{c}_{{ij}}^{1}\right|{c}_{{ij}}^{2}\right)\in {\boldsymbol{\mathscr{H}}}_{2}\,{\mathrm{and}}\,\left|{d}_{k}-{d}_{i}\right|=d\right\}.$$

The two-locus haplotype in ${{\mathcal{H}}}_{2}$ with allele cosegregated can be represented as

$$\begin{array}{l}{\boldsymbol{\mathcal{H}}}_{2}^{{\,d}{({\mathrm{cosegregated}}})}\\=\left\{\left({c}_{{kj}}^{1}\left|{c}_{{kj}}^{2},\,{c}_{{ij}}^{1}\right|{c}_{{ij}}^{2}\right)\,{{\mathrm{s}}.{\mathrm{t}}.}\,\left({c}_{{kj}}^{1}\left|{c}_{{kj}}^{2},\,{c}_{{ij}}^{1}\right|{c}_{{ij}}^{2}\right)\in {\boldsymbol{\mathcal{H}}}_{2}^{\,d},\,\,({c}_{{kj}}^{1}{c}_{{ij}}^{1} > 0\,{\mathrm{or}}\,{c}_{{kj}}^{2}{c}_{{ij}}^{2} > 0)\right\}.\end{array}$$

Thus, the two-locus LD refinement score with physical distance being *d* is calculated as

4 $$p(\boldsymbol{\mathcal{H}}_{2}^{\,d})=1-\left|{\boldsymbol{\mathcal{H}}}_{2}^{\,d\left({\mathrm{cosegregated}}\right)}\right|/\left|{\boldsymbol{\mathcal{H}}}_{2}^{\,d}\right|.$$

Regarding the three-locus mode, we first define the three-locus neighborhood index in cell *j* as

5 $${{\mathrm{Neighb}}}_{3}\left(k,i,l,j\right)=\left\{\begin{array}{l}1,\,{if}\,{{\mathrm{Neighb}}}_{2}\left(k,i,j\right)=1,\,{{\mathrm{Neighb}}}_{2}\left(i,l,j\right)=1,\,{c}_{{kj}}^{1}{c}_{{lj}}^{1} > 0\\ 0,{\mathrm{others}}\end{array}.\right.$$

The three-locus neighborhood means that the upper and lower SNVs detect the same allele. Illustration of three-locus neighborhood index can be seen in Supplementary Fig. 1b. Denote ${{\mathscr{H}}}_{3}$ as the set including all three-locus neighborhoods, we have

$$\begin{array}{l}{\boldsymbol{\mathscr{H}}}_{3}=\left\{\left({c}_{{kj}}^{1}\left|{c}_{{kj}}^{2},\,{c}_{{ij}}^{1}\right|{c}_{{ij}}^{2},\,{c}_{{lj}}^{1}{\rm{|}}{c}_{{lj}}^{2}\right)\,\mathrm{s.t.}\,{{\mathrm{Neighb}}}_{3}\left(k,i,l,j\right)\right. \\=1,\,1\le k < i < l\le {n}_{m},\,1\le j\le C\Big\}\end{array}$$

We next group ${{\mathscr{H}}}_{3}$ based on the length of haplotype as

$$\begin{array}{l}{\boldsymbol{\mathscr{H}}}_{3}^{d}\\=\left\{\left({c}_{{kj}}^{1}\left|{c}_{{kj}}^{2},\,{c}_{{ij}}^{1}\right|{c}_{{ij}}^{2},\,{c}_{{lj}}^{1}{\rm{|}}{c}_{{lj}}^{2}\right)\,\mathrm{s.t.}\,\left({c}_{{kj}}^{1}\left|{c}_{{kj}}^{2},\,{c}_{{ij}}^{1}\right|{c}_{{ij}}^{2},\,{c}_{{lj}}^{1}{\rm{|}}{c}_{{lj}}^{2}\right)\in {\boldsymbol{\mathscr{H}}}_{3}\,{\mathrm{and}}\,\left|{d}_{k}-{d}_{i}\right|=d\right\}.\end{array}$$

The three-locus haplotype in ${{\mathscr{H}}}_{3}$ with allele cosegregated can be represented as

$$\begin{array}{l}{\boldsymbol{\mathscr{H}}}_{3}^{d({\mathrm{cosegregated}})}\\=\left\{\left({c}_{{kj}}^{1}\left|{c}_{{kj}}^{2},\,{c}_{{ij}}^{1}\right|{c}_{{ij}}^{2},\,{c}_{{lj}}^{1}{\rm{|}}{c}_{{lj}}^{2}\right)\,\mathrm{s.t.}\left({c}_{{kj}}^{1}\left|{c}_{{kj}}^{2},\,{c}_{{ij}}^{1}\right|{c}_{{ij}}^{2},\,{c}_{{lj}}^{1}{\rm{|}}{c}_{{lj}}^{2}\right)\in {\boldsymbol{\mathscr{H}}}_{3}^{d}\,{\mathrm{and}}\,{c}_{{ij}}^{1} > 0\right\}.\end{array}$$

Thus, the three-locus LD refinement score with physical distance being *d* is defined as

6 $$p(\boldsymbol{\mathcal{H}}_{3}^{d})=1-\frac{\left|{\boldsymbol{\mathcal{H}}}_{3}^{d\left(\mathrm{cosegregated}\right)}\right|}{\left|{\boldsymbol{\mathcal{H}}}_{3}^{d}\right|}.$$

The two-locus and three-locus LD refinement scores $p({\mathscr{H}}_{2}^{d}),\,p({\mathscr{H}}_{3}^{d})$ can largely represent the colocalization for neighboring SNVs on a DNA haplotype or RNA transcript at the cell population level. In real data analysis, the physical distance *d* is grouped into 13 bins with <100 bp, (100 bp, 250 bp), (250 bp, 500 bp), (500 bp, 1 kb), (1 kb, 2.5 kb), (2.5 kb, 5 kb), (5 kb, 10 kb), (10 kb, 25 kb), (25 kb, 50 kb), (50 kb, 100 kb), (100 kb, 250 kb), (250 kb, 500 kb) and >500 kb.

##### Phase de novo SNVs

We next phase the de novo SNVs based on germline SNVs. Assume the genotype of de novo SNV *s* is ${A}_{s}^{1}/{A}_{s}^{2}$ and its adjacent germline SNV profile for cell *j* as follows:

$${{\boldsymbol{S}}}_{j}=\left[\begin{array}{c}\begin{array}{c}\vdots \\ {c}_{{kj}}^{1}{\rm{|}}{c}_{{kj}}^{2}\\ \vdots \end{array}\\ \begin{array}{c}{c}_{{sj}}^{1}/{c}_{{sj}}^{2}\\ \vdots \end{array}\\ \begin{array}{c}{c}_{{lj}}^{1}{\rm{|}}{c}_{{lj}}^{2}\\ \vdots \end{array}\end{array}\right]$$

where ${{\mathrm{Neighb}}}_{2}\left(k,s,j\right)=1$ and ${{\mathrm{Neighb}}}_{2}\left(s,l,j\right)=1$. ${c}_{{sj}}^{1}$ and ${c}_{{sj}}^{2}$ are the number of reads supporting allele ${A}_{s}^{1}$ and ${A}_{s}^{2}$, respectively. Due to the single-cell sparsity, it is difficult to detect allele ${A}_{s}^{1}$ and ${A}_{s}^{2}$ simultaneously in each cell.

Without loss of generality, we set $\left|{d}_{k}-{d}_{s}\right| < \left|{d}_{s}-{d}_{l}\right|$.The probability of phased genotype ${A}_{s}^{1}|{A}_{s}^{2}$ under two-locus model is

7 $${P}_{j}\left({A}_{s}^{1}|{A}_{s}^{2}\right)=\left\{\begin{array}{l}\left(\boldsymbol{\mathscr{H}}_{2}^{\left|{d}_{k}-{d}_{s}\right|}\right),\,{if}\,{c}_{{sj}}^{1}{c}_{{kj}}^{1} > 0\,{or}\,{c}_{{sj}}^{2}{c}_{{kj}}^{2} > 0\\ 1-p\left({\boldsymbol{\mathscr{H}}}_{2}^{\left|{d}_{k}-{d}_{s}\right|}\right),\,{\mathrm{others}}\end{array}.\right.$$

To derive the probability of haplotype ${A}_{s}^{1}|{A}_{s}^{2}$ under three-locus model, we need to search germline SNV *k* and *l* satisfying ${{\mathrm{Neighb}}}_{3}\left(k,s,l,j\right)=1$. Then, we have

8 $${Q}_{j}\left({A}_{s}^{1}|{A}_{s}^{2}\right)=\left\{\begin{array}{c}p\left({\boldsymbol{\mathscr{H}}}_{3}^{\left|{d}_{k}-{d}_{s}\right|}\right),\,{\mathrm{if}}\,{c}_{{sj}}^{1} > 0\\ 1-p\left({\boldsymbol{\mathscr{H}}}_{3}^{\left|{d}_{k}-{d}_{s}\right|}\right),\,{\mathrm{others}}\end{array}.\right.$$

The probability of phased genotype ${A}_{s}^{1}|{A}_{s}^{2}$ by combining two models is

9 $${p}_{j}\left({A}_{s}^{1}|{A}_{s}^{2}\right)=0.5\left({P}_{j}\left({A}_{s}^{1}|{A}_{s}^{2}\right)+{Q}_{j}\left({A}_{s}^{1}|{A}_{s}^{2}\right)\right).$$

Thus, the probability of phased genotype ${A}_{s}^{1}|{A}_{s}^{2}$ for de novo SNV *s* across the cell population is

10 $$p\left({A}_{s}^{1}|{A}_{s}^{2}\right)=\mathop{\sum }\limits_{j=1}^{C}{p}_{j}\left({A}_{s}^{1}|{A}_{s}^{2}\right)/C$$

Similarly, the probability of phased genotype ${A}_{s}^{2}|{A}_{s}^{1}$ for de novo SNV *s* across the cell population is

11 $$p\left({A}_{s}^{2}|{A}_{s}^{1}\right)=\mathop{\sum }\limits_{j=1}^{C}{p}_{j}\left({A}_{s}^{2}|{A}_{s}^{1}\right)/C$$

Based on the above definition, we have $p\left({A}_{s}^{1}|{A}_{s}^{2}\right)+p\left({A}_{s}^{2}|{A}_{s}^{1}\right)=1$. The genotype of *s* is set ${A}_{s}^{1}|{A}_{s}^{2}$ if $p\left({A}_{s}^{1}|{A}_{s}^{2}\right) > p\left({A}_{s}^{2}|{A}_{s}^{1}\right)$ and ${A}_{s}^{2}|{A}_{s}^{1}$ otherwise. The LD refinement score *p*_*s*_ is defined as ${p}_{s}={{\min }}\left\{p\left({A}_{s}^{1}|{A}_{s}^{2}\right),p\left({A}_{s}^{2}|{A}_{s}^{1}\right)\right\}$. The LD refinement score *p*_*s*_ ranges from 0 to 0.5. It is closer to 0 for a germline SNV as it has strong LD with the adjacent germline SNVs, that is, sharing the same two haplotypes in all the cells. The score is greater than 0 for a somatic SNV as the recently gained somatic allele cosegregates with germline alleles in only a subpopulation of cells. SNVs with a larger LD refinement score are classified as putative somatic SNVs (default value 0.25).

##### Cell type/cluster-level genotyping using Monovar

Monovar^18^ is then used to perform SNV genotyping on putative somatic SNVs at cluster or cell type level. Briefly, cell cluster identification can be obtained either by clustering on single-cell profiles or using reference-based cell type annotation^19^. To reduce the computational time, only reads covering these candidate loci are extracted and then split into different bam files based on their cluster identities. Monovar can be run on these bam files (each is one cluster or cell type) with default parameter settings.

### Genotyping calling evaluation

Seven single-cell samples in our study have matched WGS data that were treated as the gold standard. For each sample, only bi-allelic loci having at least one alternative allele (that is, genotype is 0/1 or 1/1) were extracted from the two call sets, denoting as *N* (Monopogen-called) and *W* (WGS-called). The sensitivity (recall) was defined as ${|N}\cap {W|}/{|W|}$ and specificity (precision) as $\frac{{|N}\cap {W|}}{{|N|}}$. The genotyping accuracy was defined as the fraction of identical genotypes in the $\left|N\cap W\right|$ overlapping SNVs. The overall accuracy was defined as the specificity multiplied by the genotype accuracy.

The genotype concordance of the Monopogen-called genotype data versus the AIDA Illumina GSAv3 genotype data was computed by first counting the number of matching alleles between the Monopogen and the Illumina GSAv3 results for loci found in both sets. The minimum possible concordance score per Monopogen calls (accounting for some match always being possible in the case of heterozygous genotypes) was subtracted, and the resulting scores were then normalized against the number of loci evaluated.

### Global and local ancestry analysis

#### PCA-projection analysis

To identify the global ancestry of single-cell sequencing samples, we downloaded genotypes from Human Genotyping Diversity Panel (HGDP), which includes 938 individuals (covering 53 populations worldwide) and 632,958 SNVs with MAF > 1%. Denote ${{\boldsymbol{R}}}_{n\times L}$ as genotypes of the HGDP samples (*n* = 938, *L* number of SNVs), and ${{\boldsymbol{g}}}_{1\times L}$ as the Monopogen-called genotype vectors from the single-cell sequencing samples (converting from GRCh38 to GRCh37 using Picard tool). Denote $${\widetilde{{\boldsymbol{R}}}}_{(n+1)\times K}=\left[\begin{array}{c}{{\boldsymbol{R}}}_{n\times L}\\ {{\boldsymbol{g}}}_{1\times L}\end{array}\right]$$. The LASER (Trace module)^48^ was used to project each sample to the HGDP. Briefly, two PCA coordinates were calculated as ${{\boldsymbol{Y}}}_{n\times K}$ and $$\left[\begin{array}{c}{\widetilde{{\boldsymbol{Y}}}}_{n\times {K}^{{\prime} }}\\ {\widetilde{{\boldsymbol{y}}}}_{1\times {K}^{{\prime} }}\end{array}\right]\,({K}^{{\prime}}\ge K)$$ by applying eigenvalue decomposition on the genetic relationship matrix (GRM) ${\boldsymbol{R}}{{\boldsymbol{R}}}^{T}$ and $\widetilde{{\boldsymbol{R}}}{\widetilde{{\boldsymbol{R}}}}^{T}$, respectively. Projection procrustes analysis was used to find an orthonormal projection matrix ${{\boldsymbol{A}}}_{{K}^{{\prime} }\times K}$ and an isotropic calling factor *ρ* such that ${{\Big|\Big|}\rho \widetilde{{\boldsymbol{Y}}}{\boldsymbol{A}}-{\boldsymbol{Y}}{\Big|\Big|}}_{F}^{2}$ is minimized, where ${{||}.{||}}_{F}^{2}$ represents the square of Frobenius norm. Once ${{\boldsymbol{A}}}_{{K}^{{\prime} }\times K}$ and *ρ* were solved, the sample-specific PCA-projection coordinates on HGDP panel can be calculated as ${\boldsymbol{y}}=\rho \widetilde{{\boldsymbol{Y}}}{\boldsymbol{A}}$. The PC coordinates of $$\left[\begin{array}{c}{{\boldsymbol{Y}}}_{n\times K}\\ {{\boldsymbol{y}}}_{1\times K}\end{array}\right]$$ were used for PCA-projection visualization.

#### Fine-scale ancestry inference

The local ancestry components of single-cell sequencing samples were calculated using RFMix tool^21^ with the phased haplotypes from the 1,000 Genomes 3 as a reference source. Monopogen-called genotypes were input to the PopPhased module with the following flags: -w 0.2, -e 1, -n 5, --use-reference-panels-in-EM, --forward-backward EM. The RFMix output was collapsed into haploid bed files, and ‘UNK’ or unknown ancestry was assigned where the posterior probability of a given ancestry was <0.90. These collapsed haploid tracts were used for local ancestry component visualization (segment size was set as 1 cM). The RFMix tool was also run on WGS genotypes from matched samples. For each segment, the ancestry component percentage for each source population was recorded. The local ancestry consistency index was calculated as the correlation of the ancestry component vector between the two call sets.

### GWAS on cellular quantitative traits

#### Variant calling on human heart left ventricle samples

There are 54 donors sequenced with snRNA-seq and 65 with snATAC-seq, among which 54 are paired. For the downstream association study, SNV calling of 54 snRNA-seq and 65 snATAC-seq samples were performed separately using Monopogen, followed by removing MAF < 10%. Variant calls were further merged for samples of paired modalities (Supplementary Table 4).

#### Cell type annotation on snRNA-seq profiles

We also downloaded the matched snRNA-seq gene expression profiles and performed a series of filtering to remove cells expressing lower than 200 and higher than 10,000 genes, and with mitochondrial gene percentages higher than 15%, using Seurat V4 (ref. ^19^).

Cell type annotation was performed by uploading all the cells of each sample to the online Azimuth heart database in Seurat V4 (ref. ^19^). Cells with predicted cell type probability scores lower than 0.9 were removed. Only cells annotated as cardiomyocytes were extracted for the downstream association study.

#### Cell type annotation on snATAC-seq profiles

Starting from the fragment files of snATAC-seq samples, we used Signac pipeline^49^ to recall peaks in each sample and combine them into a unified set after removing peaks of width <20 bp and >10 kb, leading to a total of 488,652 peaks. The gene-level chromatin accessibility was derived using GeneActivity module by aggregating peaks in gene promoters plus upstream 2 kb. The cell type annotation was also performed using the online Azimuth heart database under the same quality control criteria as in the snRNA-seq analysis.

#### Calculation of cellular quantitative traits

We used pathway expression level as a proxy for ATP metabolism level. We downloaded 216 genes from GO_ATP_METBOLIC pathway. We derived cardiac ATP metabolism level at single-cell resolution by aggregating the expression levels of 197 genes (197/216) detected in the snRNA-seq data. The calculation was performed using AddModuleScore module in Seurat. In snATAC-seq, TF *GATA4* motif-based activity was calculated for each cell using ChromVAR^50^.

#### Association study

GCTA^22^ was used to calculate a GRM among single-cell sequencing samples. The association studies on ATP metabolism level and GATA4 activity level were performed using its fastGWA-mlm option with the input of GRM and covariates as the top five ancestry PCs. Only variants with MAF > 10% were considered for association studies. The inflation factor of Quantile–Quantile plots was calculated using the R package qqman to examine whether there is population stratification in our genome-wide scan. Manhattan plot was used to show the *P* value across the whole genome with *P* = 10^−5^ as potential significant associations with cellular traits. The significant loci were further grouped into bins based on their closest genes. The nearest genes to significant loci were annotated.

### Comparison with other SNV callers

For a fair comparison with Monopogen, Samtools^15^ GATK^51^, FreeBayes^52^, Strelka2 (ref. ^53^), cellSNP^54^ and scAllele^55^ were run on bam files after the same filtering with Monopogen. For Samtools, the mpileup option was used to transform base calling and alignment information into the GL, followed by variant calling using Bcftools. The GATK was run using the HaplotypeCaller mode with default settings.

### Putative somatic SNV detection in single-cell sequencing

The 1,534 single-cell DNA bam files of sample TN28 were from breast cancer study^33^. The genotypes of the matched bulk sample were called, including ~3.5 M germline SNVs from GATK and 19,766 somatic SNVs from Mutec2 (ref. ^56^). When running Monopogen, any de novo SNVs with a predicted probability of the positive label lower than 0.5 were considered as sequencing errors. We set the physical distance threshold as 100 bp and 10 kb for two-locus mode and three-locus mode, respectively. At the evolution stage, for de novo SNVs that were not detected in bulk samples, we rechecked read alignments from bulk samples. They were not considered as sequencing errors if there was at least one read supporting the mutation. The putative somatic SNVs were annotated using OpenCravat (2.3.0)^57^, and predicted classifications on oncogenic status were obtained CScape (1.0.1)^58^ (significance level of 0.5).

The fastq file of the bone-marrow study including 10,113 cells was downloaded from MAESTER technology study^34^. When running Monopogen, any de novo SNVs with predicted probability of the positive label lower than 0.5 were considered as sequencing errors. We set the physical distance threshold as 1 kb and 50 kb for two-locus mode and three-locus mode, respectively. The variable 875 somatic SNVs (detected in at least two cells) were considered for downstream evaluation. The single-cell multi-omics profile includes mtDNA variants and TCR variable region in the same cell. To compare putative somatic SNVs with mtDNA variants, we detected whether somatic SNVs showed enrichments in specific mtDNA clone using FindMarker function (Wilcox test) in Seurat V4 (ref. ^19^). The *P* value lower than 0.01 was reported as enriched in the specific mtDNA clone. The putative somatic SNVs were grouped based on whether they were enriched in the same mtDNA clone. We then calculated the cellular concordance of each mtDNA clone as the number of cells detected in both the mtDNA clone and its matched somatic SNV group, divided by the total number of cells in the mtDNA clone. The overall concordance was the mean across all the mtDNA clones. The same scheme was used to compare somatic SNVs against TRB/A regions.

### Reporting summary

Further information on research design is available in the Nature Portfolio Reporting Summary linked to this article.

## Online content

Any methods, additional references, Nature Portfolio reporting summaries, source data, extended data, supplementary information, acknowledgements, peer review information; details of author contributions and competing interests; and statements of data and code availability are available at 10.1038/s41587-023-01873-x.

### Supplementary information

Supplementary Information Supplementary Figs. 1–15, Supplementary Tables 1–8 and Supplementary Note.

Reporting Summary

## Data Availability

The sci-ATAC profiles from the two transverse colon samples were downloaded from ENCODE database at https://www.encodeproject.org/files/ENCFF354SCV/ and https://www.encodeproject.org/files/ENCFF491HQL/. The dataset is partly from ENCODE study^59^. The matched VCF files for WGS genotypes were from accession https://www.encodeproject.org/files/ENCFF944WLM/ and https://www.encodeproject.org/files/ENCFF907ASL/. The snRNA-seq and snATAC-seq profiles from the human heart left ventricle tissues of 65 donors were downloaded from ENCODE study^60^ at https://www.encodeproject.org/matrix/?type=Experiment&assay_title=snATAC-seq&assay_title=scRNA-seq&biosample_ontology.term_name=heart+left+ventricle. The 12 scRNA-seq samples with matched WGS genotypes were downloaded from GTEx database^60^ with https://anvil.terra.bio/#workspaces/anvil-datastorage/AnVIL_GTEx_V9_hg38. The 1KG3 genotypes were from 1000 genome project^61^ and downloaded from https://ftp.1000genomes.ebi.ac.uk/vol1/ftp/data_collections/1000G_2504_high_coverage/working/20201028_3202_phased/. The HGDP panel^62^ genotypes were downloaded from http://csg.sph.umich.edu/chaolong/LASER/HGDP-938-632958.tar.gz. The scDNA-seq from the TNBC sample was downloaded from breast cancer study^33^. The single-cell RNA of bone-marrow sample used for somatic calling evolution was from MAESTER technology^34^. The fastq files were downloaded from the SRA database with SRR15598778, SRR15598779, SRR15598780, SRR15598781 and SRR15598782. The integrated single-cell multi-omics profiles including gene expressions, mtDNA variant calls and TCR profiles were downloaded from https://vangalenlab.bwh.harvard.edu/resources/maester-2021/ The single-cell profiles of 20 HBCA samples, 20 AIDA samples, and four retina samples were generated as part of the cell atlas and genetic ancestry networks organized by the Chan Zuckerberg Initiative. The 20 AIDA single-cell samples could be downloaded from https://data.humancellatlas.org/explore/projects/f0f89c14-7460-4bab-9d42-22228a91f185. The four retina single-cell samples could be downloaded from https://data.humancellatlas.org/explore/projects/f0f89c14-7460-4bab-9d42-22228a91f185. The 20 HBCA single-cell samples could be accessed through GSE195665 (https://navinlabcode.github.io/HumanBreastCellAtlas.github.io/dataAccess.html).

## Code availability

Monopogen is available in open source at https://github.com/KChen-lab/Monopogen. Scripts for reproducing key analysis results are also available at https://github.com/KChen-lab/Monopogen.
