# Supplementary material for: Single-nucleotide variant calling in single-cell sequencing data with Monopogen
Source: Nat Biotechnol. 2023 Aug 17;42(5):803–12. doi: 10.1038/s41587-023-01873-x (PMC11098741; doi:10.1038/s41587-023-01873-x)
Supplement: Supplementary file 1 — Supplementary Figs. 1–15, Supplementary Tables 1–8 and Supplementary Note. [file 41587_2023_1873_MOESM1_ESM.pdf]

nature biotechnology

Article https://doi.org/10.1038/s41587-023-01873-x

Single-nucleotide variant calling in single-cell sequencing data with Monopogen

In the format provided by the authors and unedited

Supplementary Figures

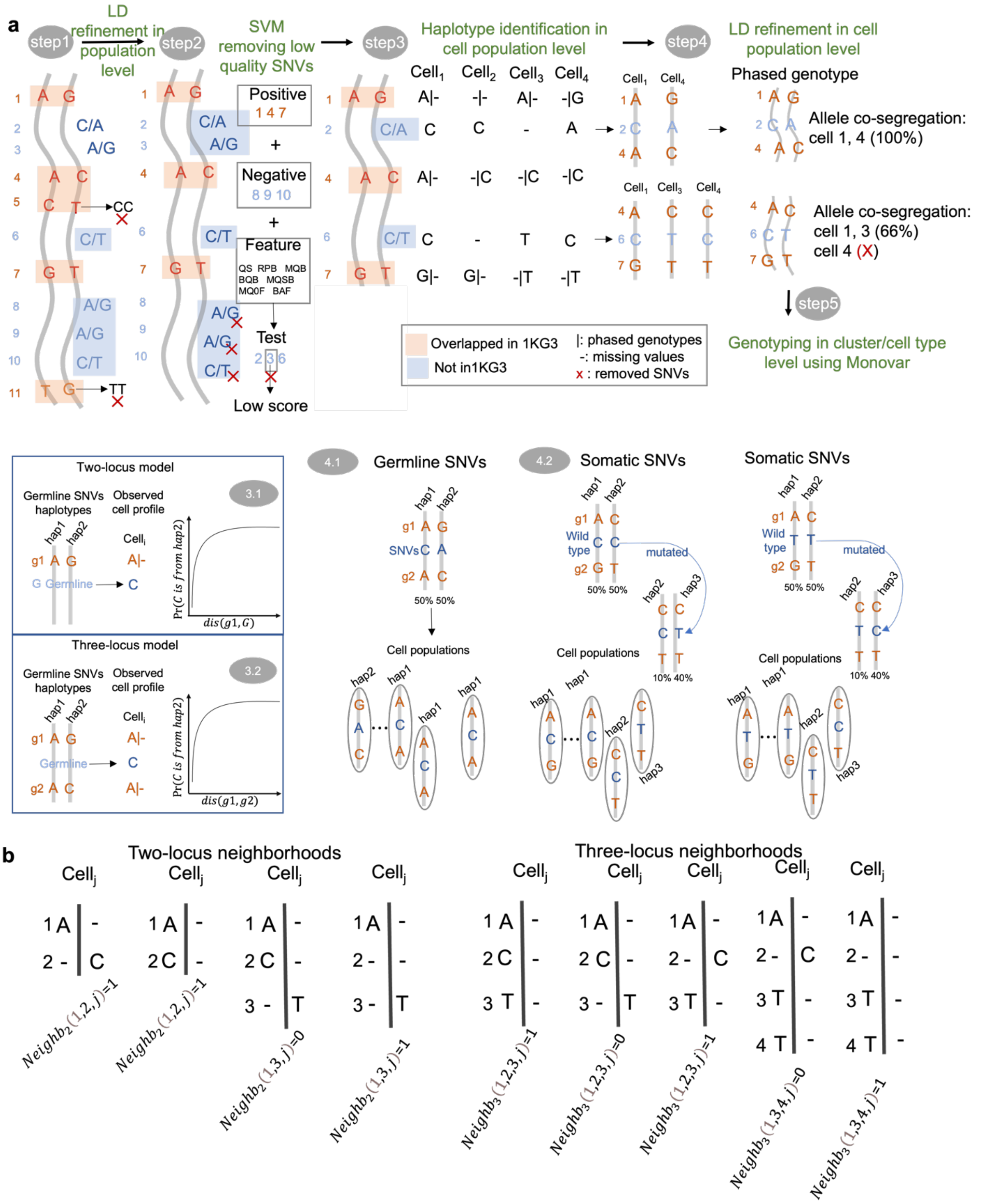

Fig.S1 Putative somatic SNV detection in cell population level. a, Workflow of putative somatic SNV detection**. Step1**: SNVs are classified into two categories based on whether they are overlapped with 1KG3 or not. SNVs 1, 4, 5, 7, 11 with orange color (phased genotype available) are germline and SNVs 2, 3, 6, 8, 9, 10 with blue color are *de novo* SNVs. Although there are SNVs observed in position 5 and 11 from single cell sequencing data, their genotypes are identified as homozygous after LD refinement using population data. Thus, SNVs 5 and 11 are removed. **Step2:** The remaining germline SNVs 1, 4, 7 are selected as the positive labels. There is a *de novo* SNVs chunk (e.g., 8, 9, 10) and no germline SNVs inside. SNVs 8,9,10 are set as negative labels. The remaining SNVs 2,3,6 are set as test set. Seven variant calling metrics provided by *Bcftools*, including quality score for calling (QS), variant distance bias for filtering splice-site artefacts (VDB), Mann-Whitney U test of read position bias (RPB), Mann-Whitney U test of base quality bias (BQB), Mann-Whitney U test of ratio of mapping quality and strand bias (MQSB), segregation-based metric (SGB) and B-allele frequency (BAF), are set as the features. The SNVs with prediction probability of positive label lower than 0.5 are removed (i.e., position 3). **Step3:** To phase SNVs 2, 6 that are not detected in 1KG3, we first search their germline neighborhoods (**see Methods**). The neighborhood searching process also be implemented on germline SNVs to estimate the empirical relationship between LD refinement score and haplotype length (as shown in **3.1** and **3.2**). For SNV 2, haplotype A-C-A in cell 1 and G-A-C in cell 4 are detected. For SNV 6, haplotype A-C-G in cell 1, C-T-T in cell 3 and C-C-T in cell 4 are detected. **Step4:** LD refinement at cell population level. The phased genotype for SNV 2 is C|A which all alleles show co-segregation at cell population level. The phased

genotype for SNV 6 is C|T which only alleles in cell 1 and 3 show co-segregations. This pattern can be used to distinguish putative somatic SNVs from germline SNVs. For germline SNVs, there are only two haplotypes available in the cell population since all cells share identical genotypes and only one haplotype is randomly selected due to the drop-out issue (**4.1**). For somatic SNVs, a proportion of wildtype allele is mutated (for example hap2 changes to hap3), thus resulting in 3 haplotypes available in the cell population. Due to allele C in hap1 (A-C-G) and hap 2 (C-C-T) in 4.2, the LD refinement score is 0.33. **b,** Examples of two-locus neighborhoods and three-locus neighborhoods used for LD refinement models**.**

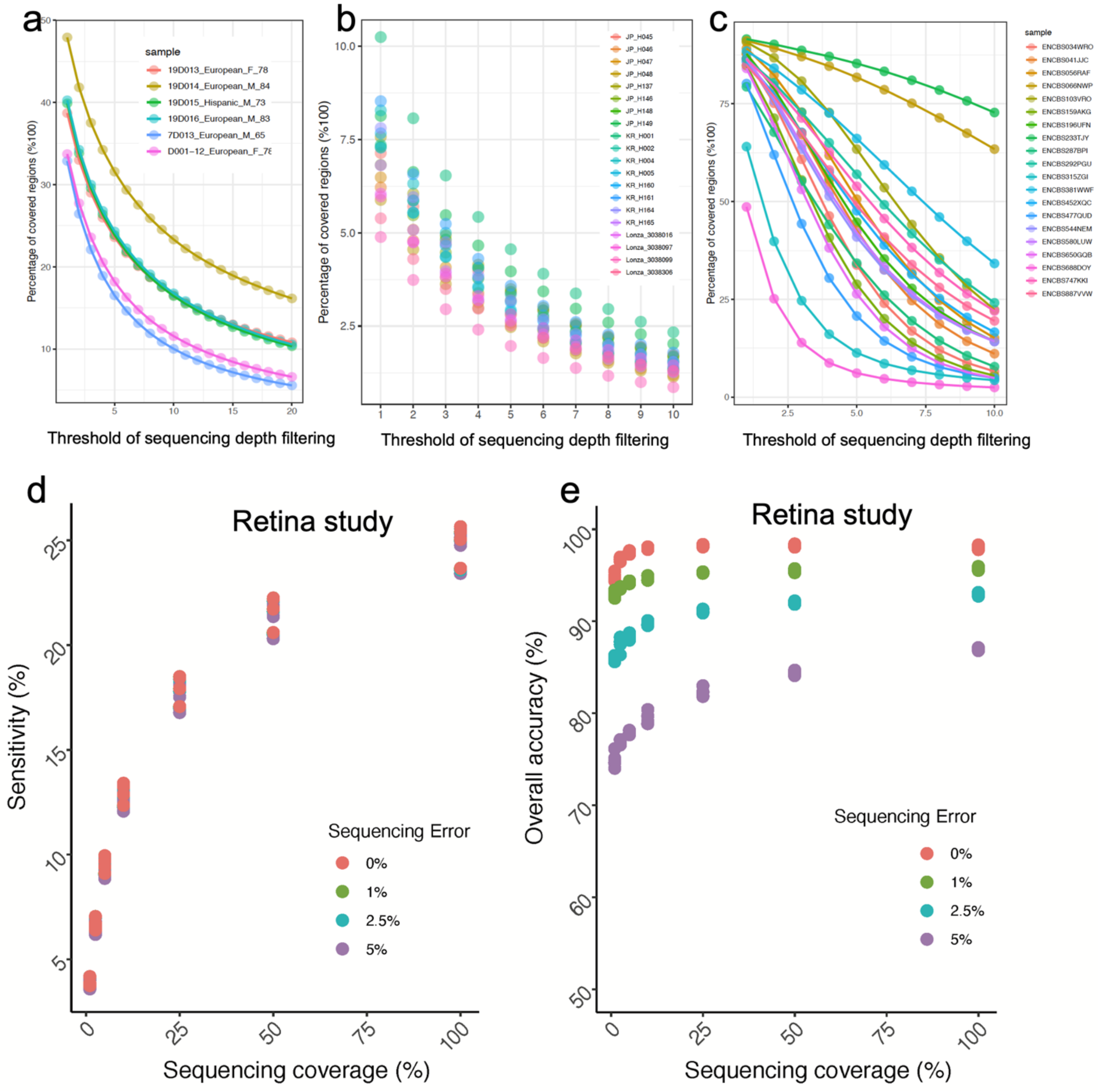
a
Percentage of covered regions (%100)
Threshold of sequencing depth filtering
b
Percentage of covered regions (%100)
Threshold of sequencing depth filtering
c
Percentage of covered regions (%100)
Threshold of sequencing depth filtering
d
Retina study
Sensitivity (%)
Sequencing coverage (%)
Sequencing Error
0%
1%
2.5%
5%
e
Retina study
Overall accuracy (%)
Sequencing coverage (%)
Sequencing Error
0%
1%
2.5%
5%

Fig.S2 Effects of sequencing coverage and sequencing error on SNV detection. a-c: Sequencing depth survey on single cell sequencing datasets. **a**, Human retina snRNA-seq data (6 samples). **b,** AIDA scRNA-seq data (20 samples), **c.** heart left ventricle snATAC-seq data (randomly select 20 samples from 65 donors), respectively. Each curve denotes results for one sample. The X-axis denotes the sequencing coverage threshold and Y-axis denotes the percentage of genome region covered by more than the specified depth threshold. **d-e:** Effects of sequencing coverage and sequencing error rate on Monopogen performance in retina snRNA-seq data. To mimic different sequencing coverage, we randomly select a fraction of reads from the whole bam file, followed by SNV calling using Monopogen. To mimic various levels of sequencing error, the artifacts are introduced into original sequencing reads with level 0%~5%. For example, 5% denotes that 5 mismatches are introduced in the sequence of 100 base pairs.

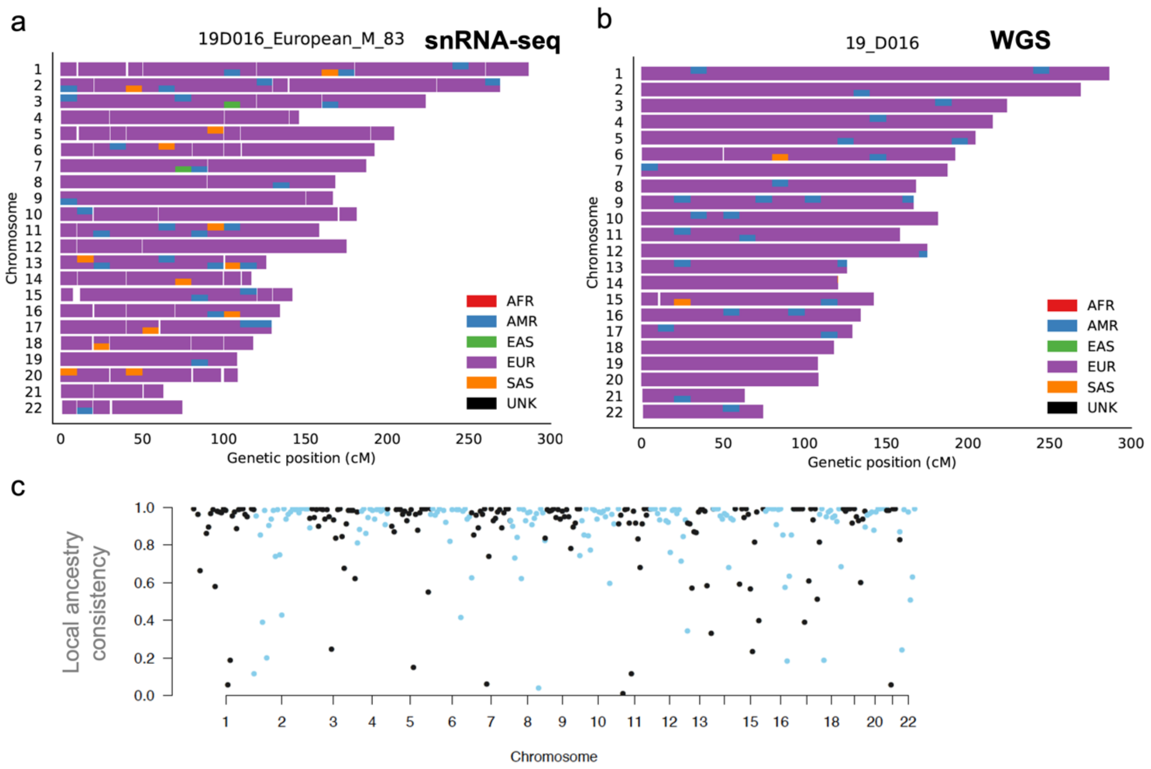

Fig S3. Local ancestry inference on 19D016 from the retina study. a-b. Local ancestry inference of a European sample 19D016 using genotypes from the snRNA-seq (**a**) and the WGS (**b**) data, respectively. The phased genotypes from 1KG3 are used as the reference for local ancestry inference. Colors in each chromosome denote the inferred source ancestry with a bin size of 1cM.

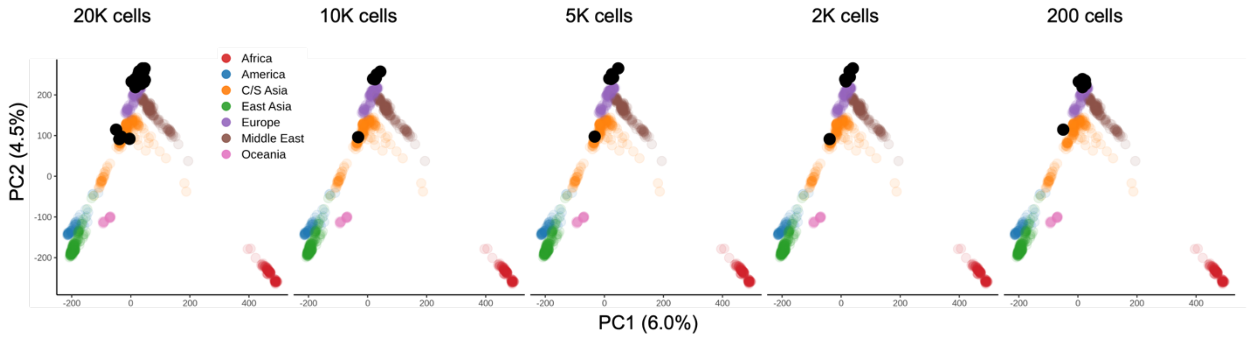

Fig. S4 Genetic ancestry of the 4 retina samples using Monopogen genotypes derived from downsampled snRNA-seq data. The down-sampling scheme is the same as the ones in **Fig. 2e-f**. Colored dots are the 938 reference individuals in the HGDP and black dots the retina samples. The explained variance of PC1 and PC2 are shown.

a

SampleID	No. of SNVs in snRNA	No. of SNVs in snATAC	No. of SNVs overlapped	01->01	01->11	11->11	11->01	Genotyping concordance
ENCDO038QFX	61767	219873	55505	28181	770	26281	269	0.981
ENCDO061UFT	65782	188923	51342	23556	518	27076	185	0.986
ENCDO068KYD	75279	143851	47721	23073	610	23825	211	0.983
ENCDO098ZMW	53898	159297	37638	17845	499	19172	116	0.984
ENCDO107KXS	81400	171295	52049	28854	546	22411	232	0.985
ENCDO110LFV	63921	152404	42933	19445	562	22742	179	0.983
ENCDO123GXG	61846	143544	43350	19680	706	22790	166	0.980
ENCDO166SII	61297	143913	40553	18786	569	21015	178	0.982
ENCDO182VFD	79736	206834	67813	33554	698	33288	269	0.986
ENCDO184BIH	64175	215130	58364	26705	745	30554	357	0.981
ENCDO187SDN	76811	205017	62442	28829	1247	32074	286	0.975
ENCDO189VVY	58598	173068	43040	20049	733	22082	171	0.979
ENCDO208BPU	58850	156928	40801	19057	511	21070	157	0.984
ENCDO212WIV	58884	224015	53345	25027	834	27264	215	0.980
ENCDO216BMM	65770	211712	55190	27463	883	26640	202	0.980
ENCDO229CTV	89106	219897	79217	39773	1191	37914	337	0.981
ENCDO239INK	67631	142027	42016	18652	527	22650	184	0.983
ENCDO240MDH	57946	81850	22469	9502	295	12506	163	0.980
ENCDO266KDE	88269	176674	68663	33153	720	34522	262	0.986
ENCDO269PFV	98215	227957	83730	45012	1510	36922	279	0.979
ENCDO308XYT	64473	246119	61736	29395	1063	31023	254	0.979
ENCDO411EVD	68500	211140	58562	29072	900	28378	203	0.981
ENCDO423HZU	82065	142341	52663	22561	656	29145	298	0.982
ENCDO435KUZ	74700	182876	59834	30125	697	28821	188	0.985
ENCDO446FXE	59777	171857	43413	20427	510	22299	173	0.984
ENCDO522PCG	63072	234226	58236	29284	766	27915	267	0.982
ENCDO528BHB	73137	208972	62160	31406	669	29849	226	0.986
ENCDO529CYH	61550	217791	53501	27150	737	25413	199	0.983
ENCDO545EJY	73119	203658	60786	21649	1988	36818	329	0.962
ENCDO575WHY	71832	226543	64474	32779	891	30583	215	0.983
ENCDO580GZH	95601	283112	89719	54356	1233	33810	314	0.983
ENCDO593PZY	94765	280204	87675	49971	1256	36044	399	0.981
ENCDO602PYC	70400	239096	66512	34436	1357	30442	270	0.976
ENCDO607DMC	60832	221842	55081	27385	617	26829	248	0.984
ENCDO639HZG	62752	221886	48676	26793	850	20860	169	0.979
ENCDO650FUH	78673	133331	48222	22188	499	25315	217	0.985
ENCDO695LFW	63466	193774	49882	24198	633	24825	218	0.983
ENCDO737TEG	79273	226480	71745	34846	1179	35437	280	0.980
ENCDO738SLM	49788	223247	44601	21633	640	22098	228	0.981
ENCDO751PQQ	85848	228343	76987	39273	1104	36265	341	0.981
ENCDO768WUT	62716	138199	37372	15588	616	21000	164	0.979
ENCDO817MDD	64853	136165	39552	19334	449	19594	168	0.984
ENCDO856ZOJ	74844	201224	62935	30583	873	31257	221	0.983
ENCDO862XAV	65561	217839	57316	28231	713	28161	203	0.984
ENCDO865TFF	72092	298313	69609	41300	883	27080	338	0.982
ENCDO874YWU	57725	205183	47113	21996	766	24166	181	0.980
ENCDO894OKI	73062	143848	45911	21565	756	23431	154	0.980
ENCDO934DRK	82939	152098	54277	25285	793	27947	246	0.981
ENCDO971VRE	76757	197174	64795	32331	1149	31047	263	0.978

b c

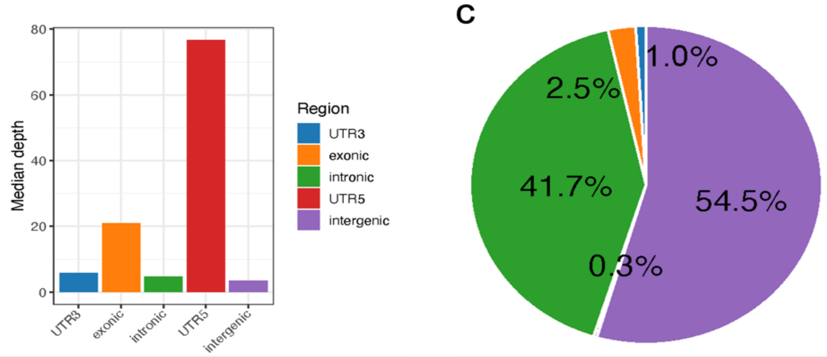

Fig. S5 Genotyping concordance between the snRNA-seq and the snATAC-seq data from the same sample. a, Genotype concordance between scRNA-seq and scATAC-seq on 54 heart left ventricle donors in the ENCODE project. Each sample has paired snRNA-seq and snATAC-seq measured. **b-c,** Distribution of germline variants

from Monopogen with median depth (**b**) and the percentage of variants (**c**) in each gene annotation category.

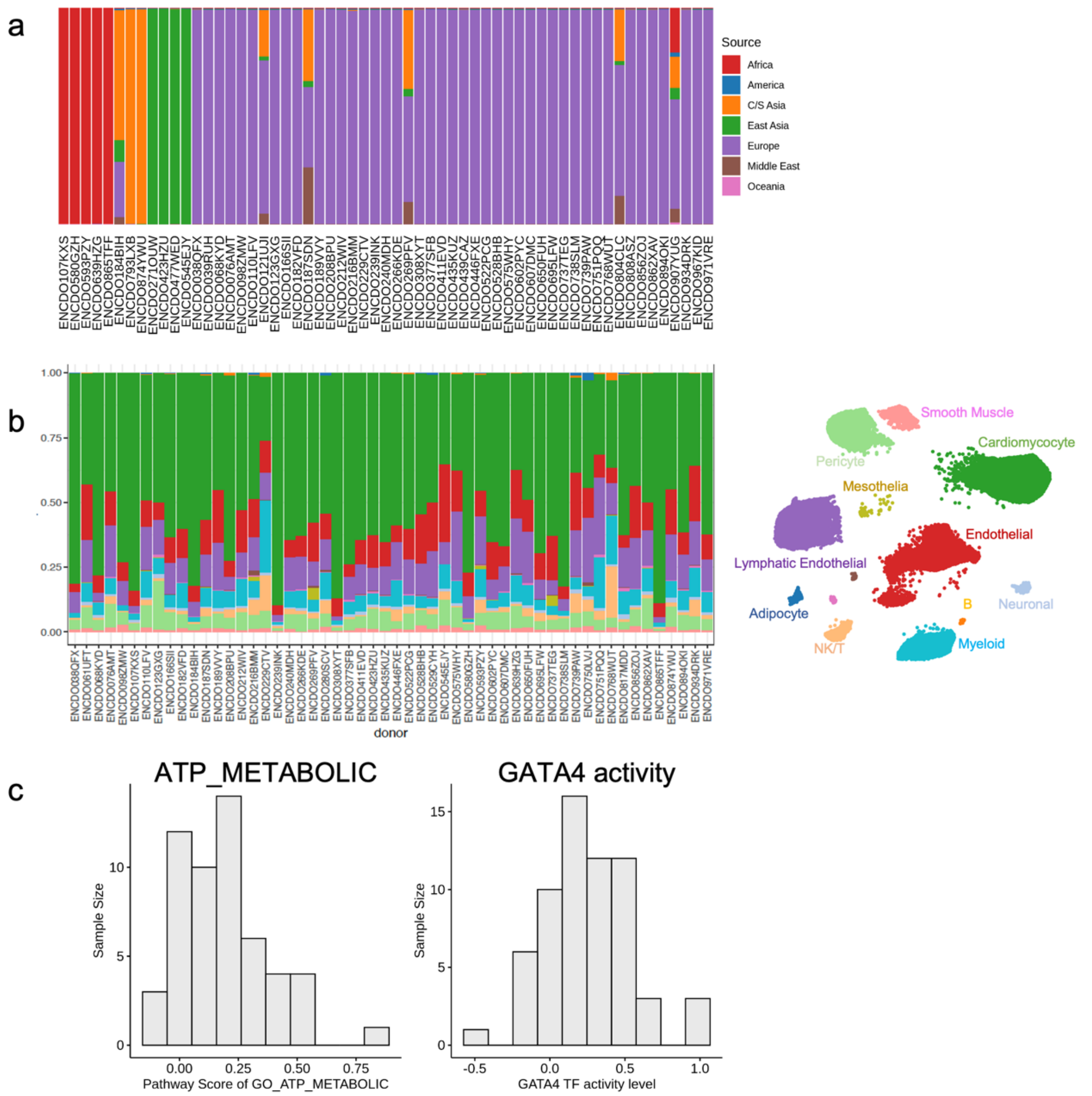

Fig.S6 Sample information on 65 heart left ventricle donors in the ENCODE project. a, Inferred cell type composition by mapping cells to Azimuth heart database. **b,** Inferred ancestry components based on genotypes from Monopogen using ADMIXTURE. **c.** Histogram distribution of calculated pathway score GO_ATP_METABOLIC. **d.** Histogram distribution of calculated *GATA4* TF activity level from chromatin accessibility level.

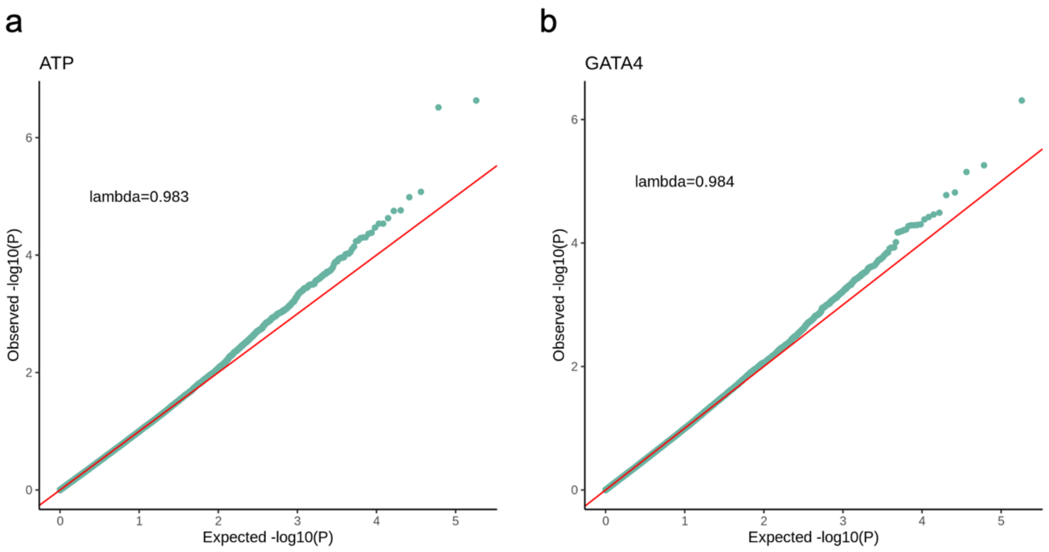

Fig.S7 Quantile-Quantile plot for the association study of GO_ATP_METABOLIC (a) and GATA4 TF activity level (b). The inflation factor value is labeled on left top corner.

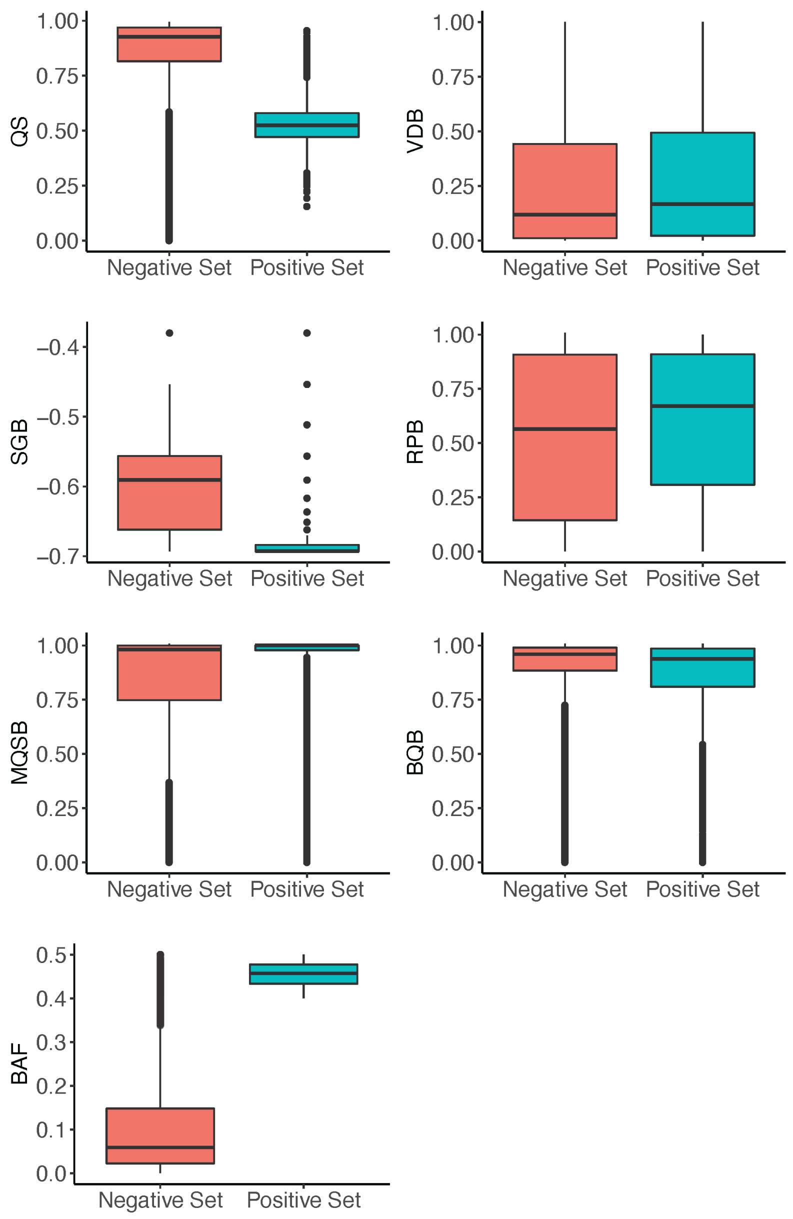

Fig. S8 Feature distribution on positive and negative labels used in the SVM module for single-cell DNA data (N=1,019,134). For each box, the centerline defines the median, the height of the box is given by the interquartile range (IQR), the whiskers are given by 1.5 × IQR and outliers are given as points beyond the minimum or maximum whisker.

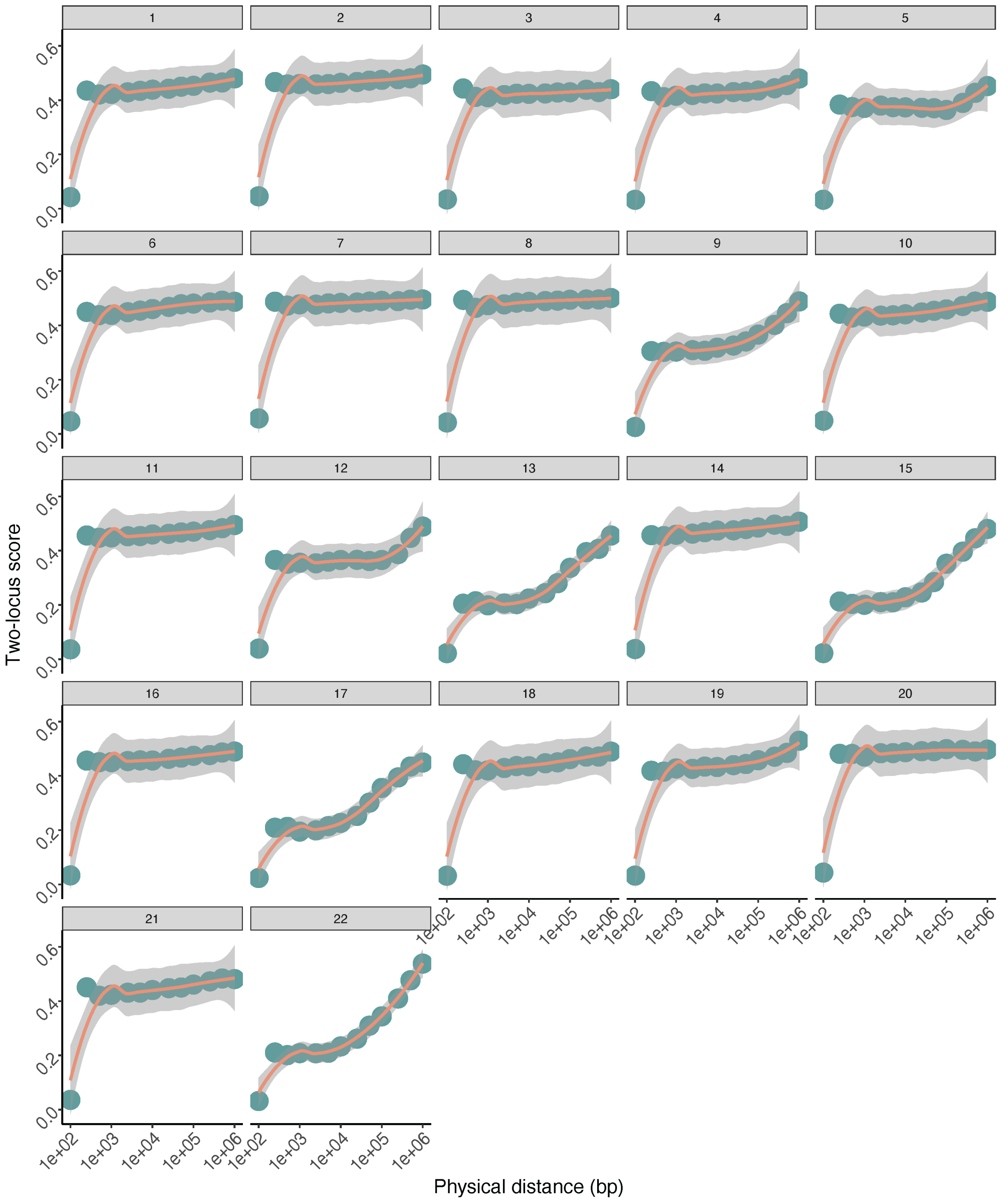

Fig S9: Two-locus LD refinement score on germline SNVs from the TNBC sample measured in single-cell DNA. Haplotypes are grouped based on their distance into 13

bins with <100bp, (100bp, 250bp), (250bp, 500bp), (500bp, 1kb), (1kb, 2.5kb), (2.5kb, 5kb), (5kb, 10kb), (10kb, 25kb), (25kb, 50kb), (50kb, 100kb), (100kb, 250kb), (250kb, 500kb) and >500kb. The X-axis is in logarithmic scale. Y-axis shows the mean value of score for all pairs within each bin together with the 95% confidence interval. Each panel shows the result from one chromosome.

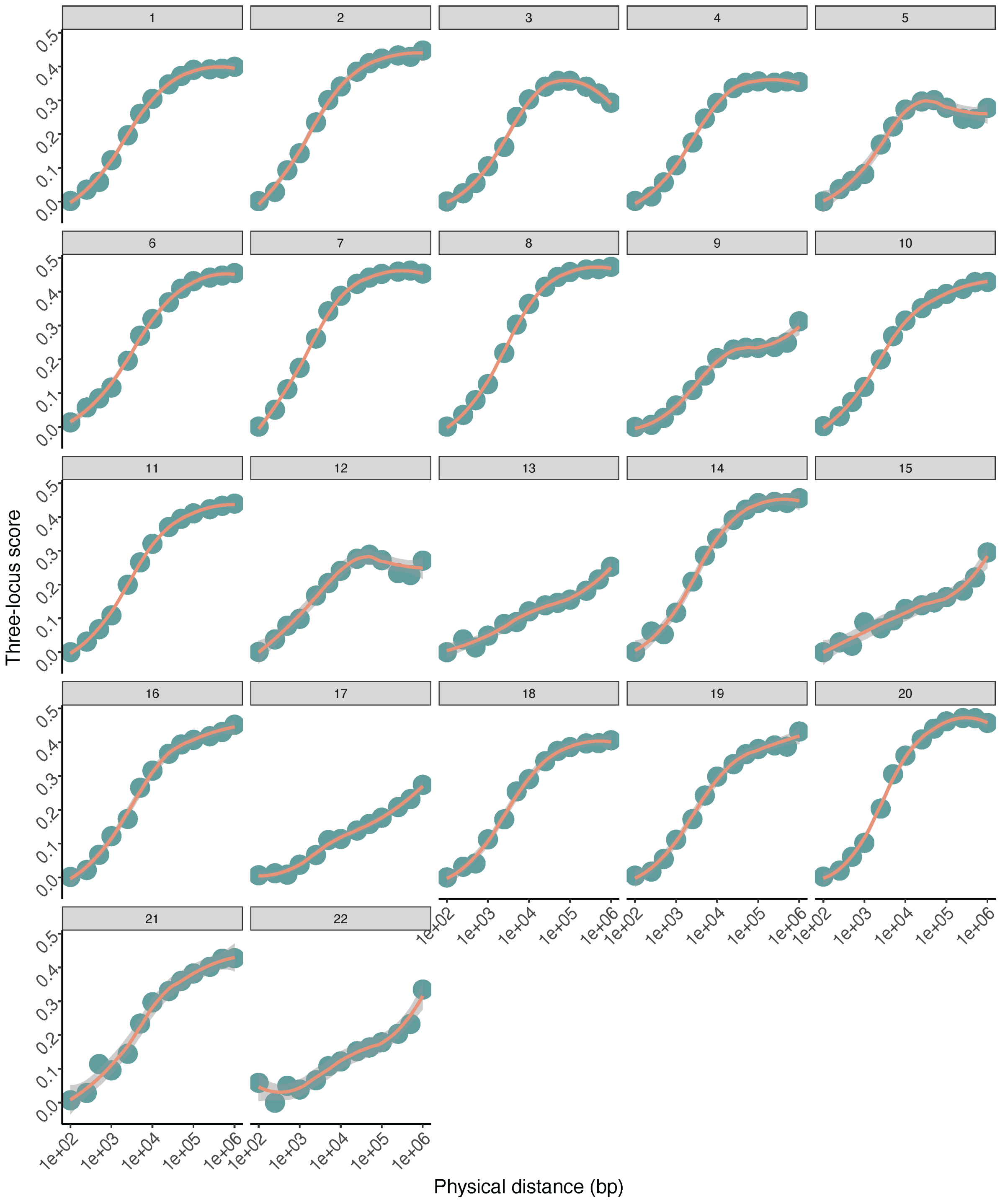

Fig. S10 Three-locus LD refinement score on germline SNVs from the TNBC sample measured in single-cell DNA. Haplotypes are grouped based on their distance into 13 bins with <100bp, (100bp, 250bp), (250bp, 500bp), (500bp, 1kb), (1kb,

2.5kb), (2.5kb, 5kb), (5kb, 10kb), (10kb, 25kb), (25kb, 50kb), (50kb, 100kb), (100kb, 250kb), (250kb, 500kb) and >500kb. The X-axis is in logarithmic scale. Y-axis shows the mean value of scores for all pairs within each bin together with the 95% confidence interval. Each panel shows the result from one chromosome.

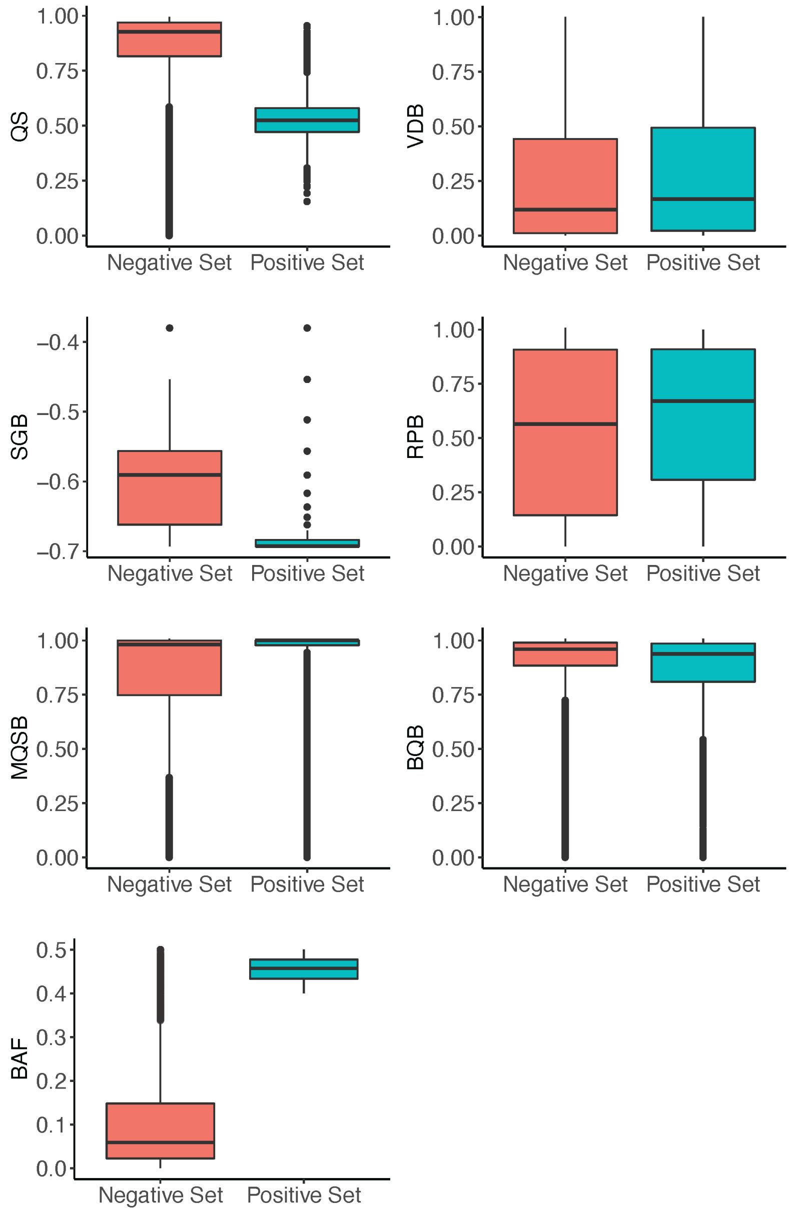

Fig. S11 Feature distribution on positive and negative labels used in the SVM module for single-cell RNA data (N=274,568**).** For each box in (**e**, **f**), the centerline defines the median, the height of the box is given by the interquartile range (IQR), the whiskers are given by 1.5 × IQR and outliers are given as points beyond the minimum or maximum whisker.

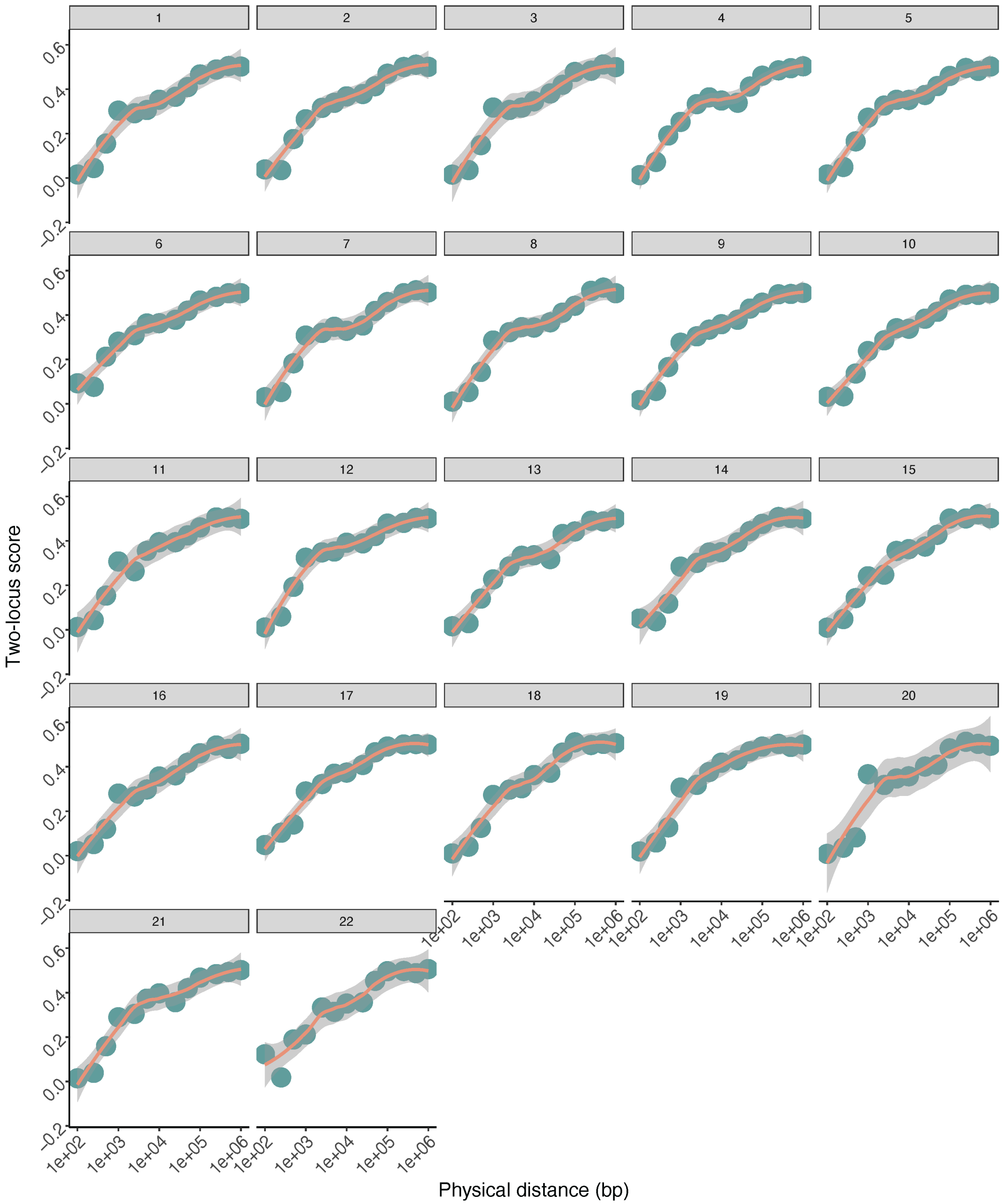

Fig. S12 Two-locus LD refinement score on germline SNVs from the TNBC sample measured in single-cell RNA data. Haplotypes are grouped based on their distance into 13 bins with <100bp, (100bp, 250bp), (250bp, 500bp), (500bp, 1kb), (1kb, 2.5kb),

(2.5kb, 5kb), (5kb, 10kb), (10kb, 25kb), (25kb, 50kb), (50kb, 100kb), (100kb, 250kb), (250kb, 500kb) and >500kb. The X-axis is in logarithmic scale. Y-axis shows the mean value of score for all pairs within each bin together with the 95% confidence interval. Each panel shows the result from one chromosome.

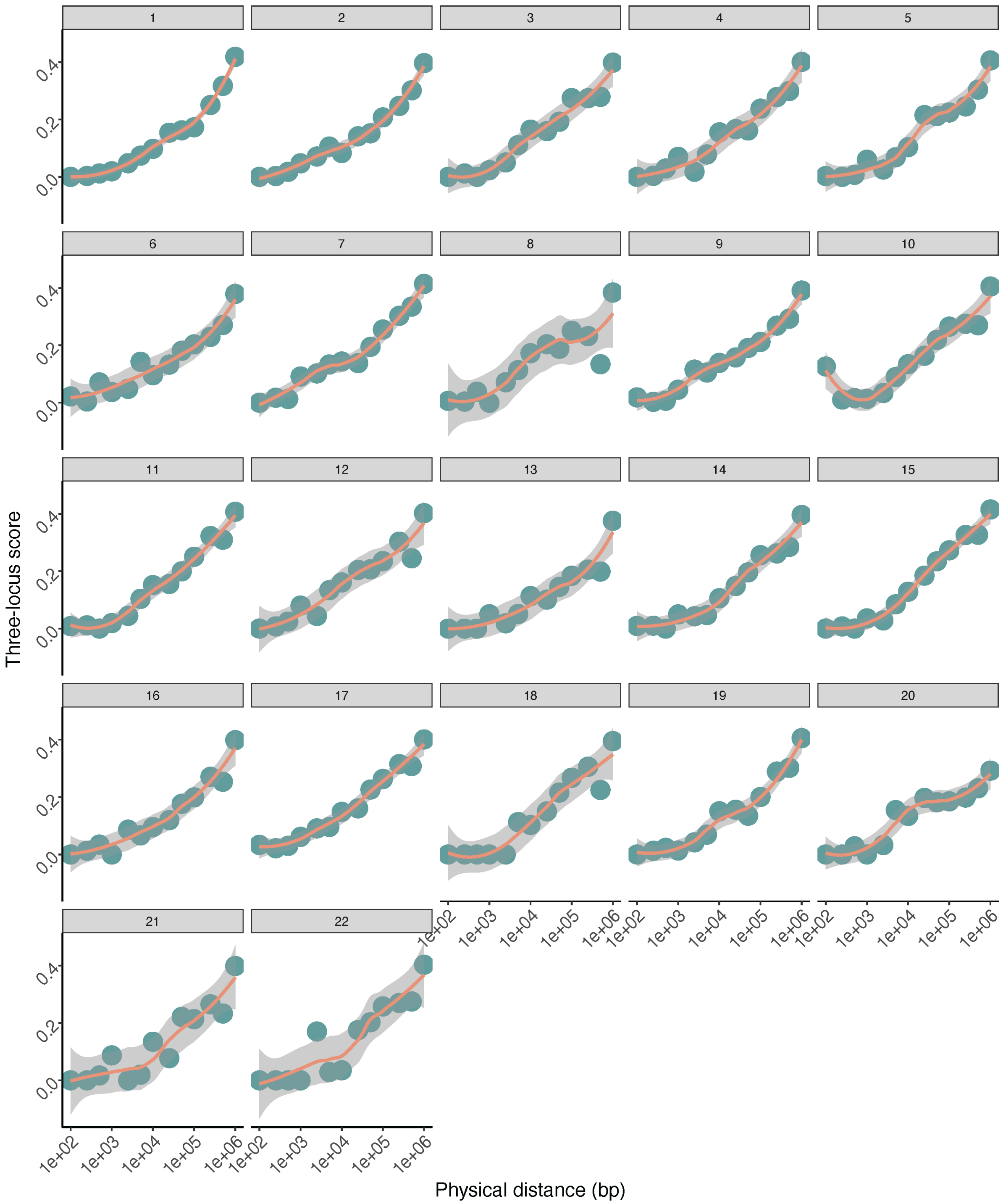

Fig. S13 Three-locus LD refinement score on germline SNVs from the TNBC sample measured in single-cell RNA data. Haplotypes are grouped based on their distance into 13 bins with <100bp, (100bp, 250bp), (250bp, 500bp), (500bp, 1kb), (1kb,

2.5kb), (2.5kb, 5kb), (5kb, 10kb), (10kb, 25kb), (25kb, 50kb), (50kb, 100kb), (100kb, 250kb), (250kb, 500kb) and >500kb. The X-axis is in logarithmic scale. Y-axis shows the mean value of score for all pairs within each bin together with the 95% confidence interval. Each panel shows the result from one chromosome.

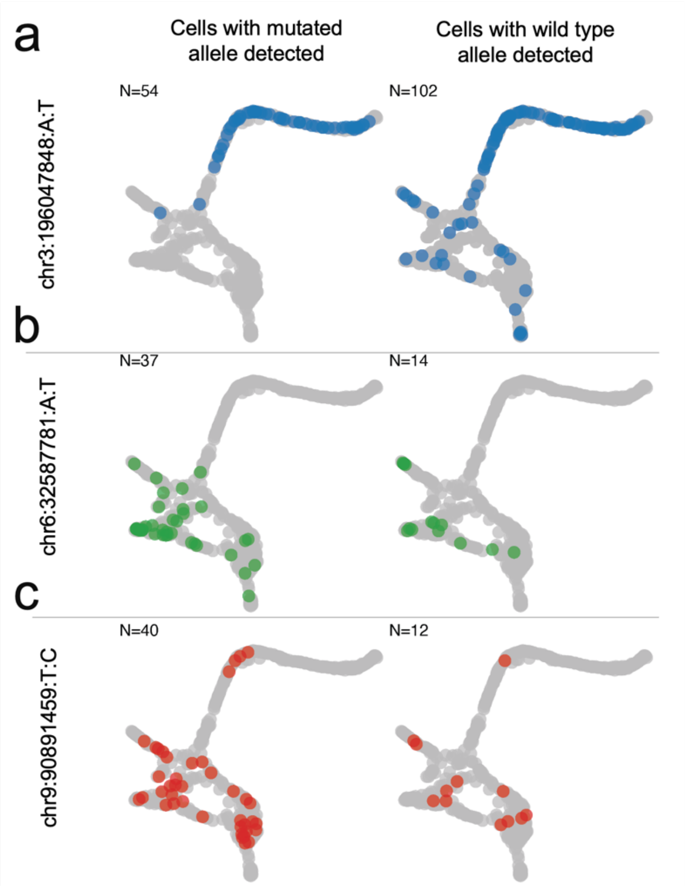

Fig. S14 UMAP displaying distribution of cells with mutated reads, wild type reads for selected somatic SNVs at cell population level (related to Fig. 5m). From top to bottom is chr3:196047848A>T (**a**), chr6:32587781A>T (**b**), and chr9:90891459T>C (**c**). The number of cells with reads detected are labeled on the top left corner in each figure.

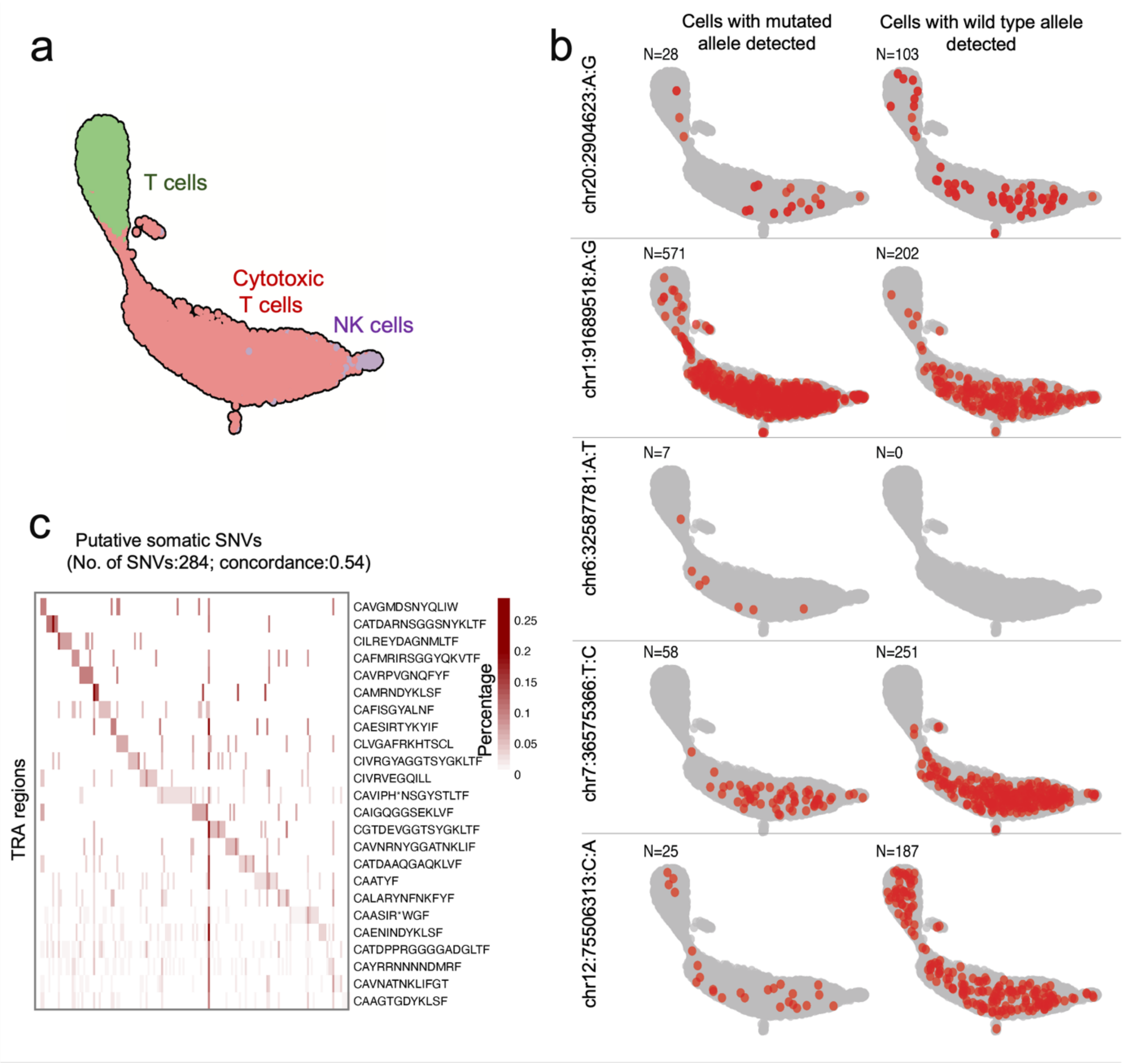

Fig. S15 Comparison between putative somatic SNVs and TCR clones from scRNA-seq data (Related to Fig. 5n-s). a. UMAPs displaying cell types annotated in T/NK cell lineages. **b,** UMAPs displaying the distribution of cells with mutated reads, wild type reads detected for selected putative somatic SNVs. The number of cells with reads detected are labeled on the top left corner in each figure. **c**, Heatmap displaying the detected percentage of putative somatic SNVs in each TRA clone. The sum of each row is 1. For TRA region evaluation, we evaluate 569 cells with both somatic SNVs and TRB region detected. There are 126 somatic SNVs (among 239 somatic SNVs detected

in at least two cells) showing TRA region enrichment (**Methods**). The cellular concordance between somatic SNV and TRA clones is 0.54.

Supplementary Tables

	Study	SampleID	No.of cells	No. of reads	No. of reads per cell	Read length	No. of SNVs in WGS (W)	No. of SNVs in single cell sequencing (N	No. of SNVs overlapping (TP)	0/1->1/1	1/1->0/1	Recall (TP/W)	Precision (TP/N)	Genotyping accuracy	Overall accuracy
snRNA-seq	Retina tissue	19D013	21016[a]	722847191	33133	150bp	4043921	830799	823045	2479	15114	0.203	0.991	0.979	0.970
		19D014	20127	717949095	35670	150bp	4069978	827731	820017	2476	15585	0.201	0.991	0.979	0.970
		19D015	26395	619591103	23473	150bp	4147482	885969	878586	2746	13108	0.212	0.992	0.982	0.974
		19D016	25340	594626499	25340	150bp	3925722	905395	892993	3276	12540	0.227	0.986	0.983	0.970
sciATAC-seq	Transverse colon	ENCDO845WKR	12122[b]	67690360	5584	50bp	3900140	1133545	1126660	4068	16289	0.289	0.994	0.982	0.976
		ENCDO793IXB	12611	61044400	4840	50bp	3728381	755565	752150	4038	28779	0.202	0.995	0.957	0.952
scDNA	Triple-Negative Breast Cancer	TN28	1534	2780180418		50bp	3709485	3229120	3105841	1924	2405	0.837	0.962	0.999	0.960

Table S1. Summary of SNV calling for three benchmarking datasets for Monopogen. [a]: cells with at least 500 genes expressed. [b]: cells with at least 500 peaks detected.

	Platform	Study	SampleID	No. of SNVs in WGS (W)	No. of SNVs in single cells (N)	No. of SNVs overlapping (TP)	0/1->1/1	1/1->0/1	Recall (TP/W)	Precision (TP/N)	Genotyping accuracy	Overall accuracy
Samtools	snRNA-seq	Retina tissue	19D013	4043921	1061214	768328	90076	3027	0.190	0.724	0.879	0.636
			19D014	4069978	1120514	828363	96285	3061	0.204	0.739	0.880	0.651
			19D015	4147482	1055049	840055	83022	2926	0.203	0.796	0.898	0.715
			19D016	3925722	1075721	826081	81474	3666	0.210	0.768	0.897	0.689
	snATAC-seq	Transverse colon	ENCDO 845WKR	3900140	188322	185654	64750	270	0.048	0.986	0.650	0.641
			ENCDO 793IXB	3728381	331891	329371	93192	40	0.088	0.992	0.717	0.711
	scDNA-seq	Triple-Negative Breast Cancer	TN28	3709485	3241101	2992186	2261	3079	0.807	0.923	0.998	0.922
GATK	snRNA-seq	Retina tissue	19D013	4043921	1068479	423234	3177	2724	0.105	0.396	0.986	0.391
			19D014	4069978	1114374	463466	3888	3016	0.114	0.416	0.985	0.410
			19D015	4147482	891334	455319	2395	2583	0.110	0.511	0.989	0.505
			19D016	3925722	926002	444972	2216	3238	0.113	0.481	0.988	0.475
	snATAC-seq	Transverse colon	ENCDO 845WKR	3900140	507967	464775	95537	627	0.119	0.915	0.793	0.726
			ENCDO 793IXB	3728381	292631	256275	53060	98	0.069	0.876	0.793	0.694
	scDNA-seq	Triple-Negative Breast Cancer	TN28	3709485	3471636	3134327	6303	5100	0.845	0.903	0.996	0.900
FreeBayes	snRNA-seq	Retina tissue	19D013	4043921	2221007	706721	70406	4395	0.175	0.318	0.894	0.285
			19D014	4069978	2448445	761853	74415	4854	0.187	0.311	0.896	0.279
			19D015	4147482	2153647	774074	64445	4056	0.187	0.359	0.912	0.328
			19D016	3925722	2195792	728609	62735	5250	0.186	0.332	0.907	0.301
	snATAC-seq	Transverse colon	ENCDO 845WKR	3900140	451171	357967	80322	196	0.096	0.793	0.775	0.615
			ENCDO 793IXB	3728381	533244	447602	107657	614	0.115	0.839	0.758	0.636
	scDNA-seq	Triple-Negative Breast Cancer	TN28	3709485	4611154	3178583	8530	5118	0.857	0.689	0.996	0.686
Strelka2	snRNA-seq	Retina tissue	19D013	4043921	3338123	950903	78358	64277	0.235	0.285	0.850	0.242
			19D014	4069978	3649163	1009174	80679	67359	0.248	0.277	0.853	0.236
			19D015	4147482	3528363	1044988	70069	66023	0.252	0.296	0.870	0.258
			19D016	3925722	3486356	973739	67219	70158	0.248	0.279	0.859	0.240
	snATAC-seq	Transverse colon	ENCDO 845WKR	3900140	1520434	904901	73670	245371	0.243	0.595	0.647	0.385
			ENCDO 793IXB	3728381	2175921	1120091	97536	296947	0.287	0.515	0.648	0.333
	scDNA-seq	Triple-Negative Breast Cancer	TN28	3709485	4802952	3217751	6736	18092	0.867	0.670	0.992	0.665
cellSNP	snRNA-seq	Retina tissue	19D013	4043921	167260	158406	1856	1249	0.039	0.947	0.980	0.928
			19D014	4069978	180773	169881	1890	2269	0.042	0.940	0.976	0.917
			19D015	4147482	171554	161715	961	1072	0.039	0.943	0.987	0.931
			19D016	3925722	172493	161512	2337	1520	0.041	0.936	0.976	0.914
	snATAC-seq	Transverse colon	ENCDO 845WKR	3900140	6768	6454	0	0	0.002	0.953	1.000	0.953
			ENCDO 793IXB	3728381	7735	7408	163	81	0.002	0.958	0.967	0.926
	scDNA-seq	Triple-Negative Breast Cancer	TN28	3709485	857082	837738	384	1202	0.226	0.977	0.998	0.976
scAllele	snRNA-seq	Retina tissue	19D013	4043921	8558369	649747	14878	208903	0.161	0.076	0.656	0.050
			19D014	4069978	9714966	690091	13924	256002	0.170	0.071	0.609	0.043
			19D015	4147482	9018650	681558	13174	226193	0.164	0.076	0.649	0.049
			19D016	3925722	8805378	630511	10106	217430	0.161	0.072	0.639	0.046
	snATAC-seq	Transverse colon	ENCDO 845WKR	3900140	424312	86578	78	31089	0.023	0.204	0.640	0.131
			ENCDO 793IXB	3728381	581288	68312	163	28528	0.018	0.118	0.580	0.068

Table S2. Summary of SNV calling for three benchmarking datasets for Samtools, GATK, FreeBayes, strelka, cellSNP and scAllele.

Fraction of clusters with SNV	SampleID	No. of SNVs called (N)	No. of SNVs overlapping in WGS (TP)	0/1->0/1	0/1->1/1	1/1->0/1	1/1->1/1	Precision (TP/N)	Genotyping accuracy	Overall accuracy
>0	19D013	129942	37674	37545	45	79	5	0.29	0.997	0.289
>0.1	19D013	124875	37616	37497	36	79	4	0.301	0.997	0.300
>0.2	19D013	106891	37228	37138	15	74	1	0.348	0.998	0.347
>0.3	19D013	83565	36334	36261	4	69	0	0.435	0.998	0.434
>0.4	19D013	64935	34919	34855	3	61	0	0.538	0.998	0.537
>0.5	19D013	51398	32558	32499	2	57	0	0.633	0.998	0.632
>0.6	19D013	41769	29500	29454	1	45	0	0.706	0.998	0.705
>0.7	19D013	33213	25027	24985	1	41	0	0.754	0.998	0.752
>0.8	19D013	24199	19079	19051	0	28	0	0.788	0.999	0.787
>0.9	19D013	12247	10031	10020	0	11	0	0.819	0.999	0.818
>0	19D014	118783	36182	35979	72	126	5	0.305	0.995	0.303
>0.1	19D014	114956	36134	35941	62	126	5	0.314	0.995	0.312
>0.2	19D014	99169	35821	35670	27	123	1	0.361	0.996	0.360
>0.3	19D014	77232	35067	34950	3	114	0	0.454	0.997	0.453
>0.4	19D014	59934	33806	33702	1	103	0	0.564	0.997	0.562
>0.5	19D014	47528	31686	31593	0	93	0	0.667	0.997	0.665
>0.6	19D014	38688	28707	28630	0	77	0	0.742	0.997	0.740
>0.7	19D014	30583	24332	24265	0	67	0	0.796	0.997	0.794
>0.8	19D014	22171	18406	18359	0	47	0	0.83	0.997	0.828
>0.9	19D014	11150	9463	9432	0	31	0	0.849	0.997	0.846
>0	19D015	69548	29467	29370	34	59	4	0.424	0.997	0.423
>0.1	19D015	67710	29441	29351	27	59	4	0.435	0.997	0.434
>0.2	19D015	59270	29201	29136	8	55	2	0.493	0.998	0.492
>0.3	19D015	48496	28717	28663	1	52	1	0.592	0.998	0.591
>0.4	19D015	40188	27902	27851	1	50	0	0.694	0.998	0.693
>0.5	19D015	34524	26581	26537	0	44	0	0.77	0.998	0.768
>0.6	19D015	30417	24877	24836	0	41	0	0.818	0.998	0.816
>0.7	19D015	26685	22655	22622	0	33	0	0.849	0.999	0.848
>0.8	19D015	20575	18207	18181	0	26	0	0.885	0.999	0.884
>0.9	19D015	12066	10952	10939	0	13	0	0.908	0.999	0.907
>0	19D016	79931	30432	30258	47	123	4	0.381	0.994	0.379
>0.1	19D016	77746	30394	30230	37	123	4	0.391	0.995	0.389
>0.2	19D016	67693	30187	30050	13	120	4	0.446	0.996	0.444
>0.3	19D016	54813	29641	29520	3	117	1	0.541	0.996	0.539
>0.4	19D016	44836	28825	28712	0	112	1	0.643	0.996	0.640
>0.5	19D016	38119	27556	27449	0	106	1	0.723	0.996	0.720
>0.6	19D016	33143	25756	25663	0	92	1	0.777	0.996	0.774
>0.7	19D016	28721	23278	23196	0	82	0	0.81	0.996	0.807
>0.8	19D016	22224	18922	18859	0	63	0	0.851	0.997	0.848
>0.9	19D016	12548	10972	10942	0	30	0	0.874	0.997	0.871

Table S3. Summary of novel SNVs detected from Monopogen in 4 retina snRNA-seq samples.

	SampleID	No. of cells	No. of reads	No. of reads per cell	Read length	No. of germline SNVs
Asia Immune Diversity Study	JP_H045	1569	59239054	37755	150bp	121313
	JP_H046	1189	39486450	33209	150bp	99964
	JP_H047	1221	41068349	33635	150bp	110858
	JP_H048	1142	37410391	32758	150bp	93531
	JP_H137	1197	45362685	37896	150bp	124220
	JP_H146	1117	44239330	39605	150bp	112796
	JP_H148	1112	44318390	39854	150bp	120655
	JP_H149	1411	57020159	40411	150bp	132721
	KR_H001	1264	57476150	45471	150bp	163256
	KR_H002	840	35435379	42184	150bp	118850
	KR_H004	821	34368945	41862	150bp	128936
	KR_H005	918	35884600	39089	150bp	114778
	KR_H160	1279	47380301	37044	150bp	120229
	KR_H161	1573	57235627	36386	150bp	131808
	KR_H164	1573	57235627	36386	150bp	120423
	KR_H165	1602	52719939	32908	150bp	103942
	Lonza_3038016	1300	40815193	31396	150bp	102320
	Lonza_3038097	999	44136732	44180	150bp	98936
	Lonza_3038099	1049	48096207	45849	150bp	77076
	Lonza_3038306	761	23257052	30561	150bp	82056
Human Breast Cell Atlas	HBCA02C	1709	324492736	189872	125bp	256088
	HBCA02i	2232	331161319	148369	125bp	369781
	HBCA03C	1865	356381426	191089	125bp	471241
	HBCA03Ccryo	1525	348639606	228616	125bp	310490
	HBCA03i	1470	356132757	242267	125bp	442943
	HBCA04C	1810	357352385	197432	125bp	380657
	HBCA04i	759	349784479	460849	125bp	390222
	HBCA04T	1608	348689072	216846	125bp	352607
	HBCA05C	464	362981567	782287	125bp	441287
	HBCA05Ccryo	1145	349034669	304833	125bp	293419
	HBCA05i	1663	348892182	209796	125bp	430660
	HBCA06C	1045	330866587	316618	125bp	390307
	HBCA07C	1405	320441257	228072	125bp	379910
	HBCA07i	2451	319876418	130508	125bp	429340
	HBCA07T	2226	314283146	141187	125bp	454028
	HBCA09C	1231	319872861	259847	125bp	385241
	HBCA09i	2027	323445690	159568	125bp	314550
	HBCA10C	1466	362133748	247021	125bp	267024
	HBCA10Ccryo	1297	348249946	268504	125bp	300322
	HBCA10i	891	345961110	388284	125bp	237928
GETx	GTEX-13N11	5226	119605363	22887	100bp	300538
	GTEX-144GM	5289	89603310	16941	100bp	181382
	GTEX-15CHR	3508	115170743	32830	100bp	155046
	GTEX-15RIE	5001	114957894	22987	100bp	205432
	GTEX-15SB6	4819	128624204	26691	100bp	304507
	GTEX-16BQI	5269	116701973	22148	100bp	211307
	GTEX-1ICG6	4148	109050028	26289	100bp	216682

Table S4. Summary of SNV calling for AIDA, HBCA, and GTEx cohorts.

SampleID	No. of SNVs in snRNA-seq	No. of SNVs in snATAC-seq	SampleID	No. of SNVs in snRNA-seq	No. of SNVs in snATAC-seq
ENCDO738SLM	537076	2602120	ENCDO271OUW	N.A	2468503
ENCDO575WHY	788194	2599335	ENCDO793LXB	N.A	2590288
ENCDO377SFB	646028	2595870	ENCDO189VVY	698813	2601870
ENCDO411EVD	790506	2592981	ENCDO123GXG	726431	2594985
ENCDO650FUH	949924	2609254	ENCDO266KDE	1031286	2591638
ENCDO229CTV	998609	2591653	ENCDO182VFD	942648	2601761
ENCDO934DRK	984814	2603980	ENCDO308XYT	704328	2600948
ENCDO269PFV	1176624	2593567	ENCDO239INK	802663	2591464
ENCDO107KXS	954463	2460631	ENCDO971VRE	939773	2587254
ENCDO435KUZ	903239	2595532	ENCDO423HZU	892430	2459095
ENCDO184BIH	760618	2530747	ENCDO593PZY	1061712	2453326
ENCDO602PYC	752144	2594180	ENCDO862XAV	719859	2610909
ENCDO038QFX	676060	2596614	ENCDO212WIV	642434	2579584
ENCDO216BMM	707303	2619222	ENCDO280SCV	671894	2599335
ENCDO528BHB	780800	2596634	ENCDO817MDD	693944	2623213
ENCDO737TEG	881202	2608524	ENCDO607DMC	685762	2587715
ENCDO061UFT	759082	2617048	ENCDO446FXE	690928	2597200
ENCDO751PQQ	1063673	2608314	ENCDO110LFV	710207	2599868
ENCDO874YWU	632607	2587876	ENCDO477WED	N.A	2481147
ENCDO639HZG	706263	2462721	ENCDO768WUT	680723	2599976
ENCDO545EJY	804425	2446069	ENCDO208BPU	649945	2589330
ENCDO739PAW	592706	2602392	ENCDO098ZMW	634326	2589542
ENCDO750LVJ	1038750	2589178	ENCDO695LFW	702332	2618752
ENCDO580GZH	1085464	2466424	ENCDO907YUG	N.A	2964332
ENCDO894OKI	881239	2588923	ENCDO967KID	N.A	2596910
ENCDO187SDN	908590	2603997	ENCDO804CLC	N.A	2585524
ENCDO865TFF	790664	2459535	ENCDO926KEV	N.A	2693594
ENCDO076AMT	418685	2601072	ENCDO808ASZ	N.A	2627648
ENCDO068KYD	886965	2599461	ENCDO439CAZ	N.A	2605541
ENCDO522PCG	706117	2613923	ENCDO039RUH	N.A	2607601
ENCDO529CYH	669832	2615742	ENCDO121UJI	N.A	2633926
ENCDO856ZOJ	888770	2600768	ENCDO240MDH	639683	2597854
			ENCDO166SII	658079	2587348

Table S5. Summary of SNV calling from heart left ventricle samples. N.A: not available.

rsID	CHR	POS	A1	A2	AF1	BETA	SE	P	distancetoFeature	symbol
rs12748496	1	59020879	A	G	0.265	0.168	0.038	8.08E-06	87235	LINC01358
rs12124171	1	80188832	G	A	0.204	-0.206	0.042	8.70E-07	-346924	LINC01781
rs10159130	1	80192436	C	T	0.204	-0.206	0.042	8.70E-07	-343320	LINC01781
rs11162948	1	80192975	C	T	0.204	-0.206	0.042	8.70E-07	-342781	LINC01781
rs17104484	1	80193168	C	A	0.204	-0.206	0.042	8.70E-07	-342588	LINC01781
rs11162949	1	80195006	G	A	0.204	-0.206	0.042	8.70E-07	-340750	LINC01781
rs10782722	1	80197213	G	A	0.204	-0.206	0.042	8.70E-07	-338543	LINC01781
rs10782723	1	80197498	C	G	0.204	-0.206	0.042	8.70E-07	-338258	LINC01781
rs1529999	2	118476740	T	C	0.500	-0.128	0.028	5.87E-06	370939	EN1
rs4849701	2	118478920	C	T	0.510	-0.139	0.028	6.15E-07	368759	EN1
rs6542466	2	118481948	A	G	0.520	-0.133	0.028	1.72E-06	365731	EN1
rs4848510	2	118482581	A	C	0.449	-0.129	0.028	4.12E-06	365098	EN1
rs1344896	2	118492854	T	C	0.449	-0.129	0.028	4.12E-06	354825	EN1
rs6752749	2	118499583	G	C	0.449	-0.129	0.028	4.12E-06	348096	EN1
rs13009652	2	138959515	C	T	0.265	0.179	0.040	7.79E-06	-179166	NXPH2
rs1710827	2	235760076	A	G	0.296	0.160	0.030	6.52E-08	254324	AGAP1-IT1
rs2696393	2	235760409	G	A	0.296	0.160	0.030	6.52E-08	254657	AGAP1-IT1
rs2696394	2	235760459	C	G	0.296	0.160	0.030	6.52E-08	254707	AGAP1-IT1
rs2696395	2	235760542	A	G	0.296	0.160	0.030	6.52E-08	254790	AGAP1-IT1
rs2696396	2	235760568	G	A	0.296	0.160	0.030	6.52E-08	254816	AGAP1-IT1
rs2675142	2	235760894	G	A	0.296	0.160	0.030	6.52E-08	255142	AGAP1-IT1
rs2696397	2	235761153	T	C	0.296	0.160	0.030	6.52E-08	255401	AGAP1-IT1
rs72975265	2	235761194	G	A	0.296	0.160	0.030	6.52E-08	255442	AGAP1-IT1
rs12469618	2	235761287	G	A	0.296	0.160	0.030	6.52E-08	255535	AGAP1-IT1
rs12469659	2	235761387	G	A	0.296	0.160	0.030	6.52E-08	255635	AGAP1-IT1
rs6714660	2	235761752	C	T	0.296	0.160	0.030	6.52E-08	256000	AGAP1-IT1
rs6715112	2	235762292	G	A	0.296	0.160	0.030	6.52E-08	256540	AGAP1-IT1
rs6718263	2	235762347	G	T	0.296	0.160	0.030	6.52E-08	256595	AGAP1-IT1
rs2696403	2	235762546	A	G	0.296	0.160	0.030	6.52E-08	256794	AGAP1-IT1
rs2696402	2	235762632	T	C	0.296	0.160	0.030	6.52E-08	256880	AGAP1-IT1
rs2696401	2	235762650	T	C	0.286	0.166	0.031	8.09E-08	256898	AGAP1-IT1
rs2675141	2	235762859	C	T	0.296	0.160	0.030	6.52E-08	257107	AGAP1-IT1
rs3896943	2	235762882	A	G	0.296	0.160	0.030	6.52E-08	257130	AGAP1-IT1
rs938492	2	235762995	C	G	0.306	0.157	0.030	2.33E-07	257243	AGAP1-IT1
rs938491	2	235763052	G	A	0.316	0.153	0.031	7.22E-07	257300	AGAP1-IT1
rs2696405	2	235763462	C	T	0.306	0.157	0.030	2.33E-07	257710	AGAP1-IT1
rs2696406	2	235763593	G	T	0.306	0.157	0.030	2.33E-07	257841	AGAP1-IT1
rs2696407	2	235763618	C	T	0.306	0.157	0.030	2.33E-07	257866	AGAP1-IT1
rs552845949	2	235764030	G	T	0.286	0.157	0.032	8.88E-07	258278	AGAP1-IT1
rs2675139	2	235764071	G	A	0.306	0.157	0.030	2.33E-07	258319	AGAP1-IT1
rs2696409	2	235764087	C	T	0.316	0.156	0.031	3.03E-07	258335	AGAP1-IT1
rs2696410	2	235764169	A	G	0.306	0.157	0.030	2.33E-07	258417	AGAP1-IT1
rs2675138	2	235764317	T	C	0.306	0.157	0.030	2.33E-07	258565	AGAP1-IT1
rs2675136	2	235764447	T	C	0.306	0.157	0.030	2.33E-07	258695	AGAP1-IT1
rs2675135	2	235764536	G	C	0.296	0.161	0.032	3.06E-07	258784	AGAP1-IT1
rs6712556	2	235765406	T	C	0.306	0.157	0.030	2.33E-07	259654	AGAP1-IT1
rs6712558	2	235765411	T	C	0.306	0.157	0.030	2.33E-07	259659	AGAP1-IT1
rs2675134	2	235765760	C	T	0.306	0.157	0.030	2.33E-07	260008	AGAP1-IT1
rs2675133	2	235766294	T	C	0.296	0.161	0.032	3.06E-07	260542	AGAP1-IT1
rs2696386	2	235766387	A	G	0.296	0.161	0.032	3.06E-07	260635	AGAP1-IT1
rs2696387	2	235766438	T	G	0.306	0.157	0.030	2.33E-07	260686	AGAP1-IT1
rs2675132	2	235767018	G	A	0.296	0.161	0.032	3.06E-07	261266	AGAP1-IT1
rs2675131	2	235767377	T	C	0.296	0.161	0.032	3.06E-07	261625	AGAP1-IT1
rs2696388	2	235767450	C	T	0.296	0.161	0.032	3.06E-07	261698	AGAP1-IT1
rs2675130	2	235767464	C	T	0.296	0.161	0.032	3.06E-07	261712	AGAP1-IT1
rs2696389	2	235767675	G	C	0.296	0.161	0.032	3.06E-07	261923	AGAP1-IT1

rs2675129	2	235767934	G	A	0.296	0.161	0.032	3.06E-07	262182	AGAP1-IT1
rs2696391	2	235768052	T	G	0.296	0.161	0.032	3.06E-07	262300	AGAP1-IT1
rs2696392	2	235768704	G	C	0.296	0.161	0.032	3.06E-07	262952	AGAP1-IT1
rs2675128	2	235769187	G	A	0.296	0.161	0.032	3.06E-07	263435	AGAP1-IT1
rs1728290	2	235770056	G	A	0.296	0.161	0.032	3.06E-07	264304	AGAP1-IT1
rs55907328	2	235770216	C	T	0.296	0.161	0.032	3.06E-07	264464	AGAP1-IT1
rs55749012	2	235770415	A	G	0.296	0.161	0.032	3.06E-07	264663	AGAP1-IT1
rs55797253	2	235770425	G	A	0.296	0.161	0.032	3.06E-07	264673	AGAP1-IT1
rs3108498	2	235770557	C	T	0.306	0.157	0.030	2.33E-07	264805	AGAP1-IT1
rs1710823	2	235770800	T	C	0.296	0.161	0.032	3.06E-07	265048	AGAP1-IT1
rs1710822	2	235770942	A	C	0.306	0.157	0.030	2.33E-07	265190	AGAP1-IT1
rs2675127	2	235771440	C	G	0.296	0.161	0.032	3.06E-07	265688	AGAP1-IT1
rs2675126	2	235771482	C	T	0.296	0.161	0.032	3.06E-07	265730	AGAP1-IT1
rs2675125	2	235771605	G	A	0.296	0.161	0.032	3.06E-07	265853	AGAP1-IT1
rs55926594	2	235771838	T	C	0.296	0.161	0.032	3.06E-07	266086	AGAP1-IT1
rs56071605	2	235771872	T	C	0.296	0.161	0.032	3.06E-07	266120	AGAP1-IT1
rs117993231	2	235771999	T	C	0.286	0.162	0.033	1.36E-06	266247	AGAP1-IT1
rs115957313	2	235772047	G	A	0.296	0.161	0.032	3.06E-07	266295	AGAP1-IT1
rs3108499	2	235772298	G	A	0.296	0.161	0.032	3.06E-07	266546	AGAP1-IT1
rs3106791	2	235772312	T	C	0.296	0.161	0.032	3.06E-07	266560	AGAP1-IT1
rs3106792	2	235772645	T	C	0.296	0.161	0.032	3.06E-07	266893	AGAP1-IT1
rs3108501	2	235772820	A	G	0.296	0.161	0.032	3.06E-07	267068	AGAP1-IT1
rs72977127	2	235773027	C	T	0.296	0.161	0.032	3.06E-07	267275	AGAP1-IT1
rs2696413	2	235773216	T	G	0.296	0.161	0.032	3.06E-07	267464	AGAP1-IT1
rs2696412	2	235774212	C	T	0.296	0.161	0.032	3.06E-07	268460	AGAP1-IT1
rs2696411	2	235774270	C	T	0.296	0.161	0.032	3.06E-07	268518	AGAP1-IT1
rs2249999	2	235774743	A	C	0.296	0.161	0.032	3.06E-07	268991	AGAP1-IT1
rs1356223	2	235774940	G	A	0.296	0.161	0.032	3.06E-07	269188	AGAP1-IT1
rs1400147	2	235775059	G	A	0.296	0.161	0.032	3.06E-07	269307	AGAP1-IT1
rs2292704	2	235775848	C	T	0.296	0.161	0.032	3.06E-07	270096	AGAP1-IT1
rs2292705	2	235775902	C	T	0.296	0.161	0.032	3.06E-07	270150	AGAP1-IT1
rs12465436	2	235776372	C	T	0.306	0.161	0.032	4.11E-07	270620	AGAP1-IT1
rs12468185	2	235776373	C	G	0.306	0.161	0.032	4.11E-07	270621	AGAP1-IT1
rs12468221	2	235776470	C	G	0.296	0.161	0.032	3.06E-07	270718	AGAP1-IT1
rs72981174	2	235776777	A	C	0.296	0.161	0.032	3.06E-07	271025	AGAP1-IT1
rs72981176	2	235776888	A	G	0.296	0.161	0.032	3.06E-07	271136	AGAP1-IT1
rs113739109	2	235777087	C	G	0.296	0.161	0.032	3.06E-07	271335	AGAP1-IT1
rs138493201	2	235777118	G	A	0.306	0.161	0.032	4.11E-07	271366	AGAP1-IT1
rs12151454	2	235777185	C	T	0.296	0.161	0.032	3.06E-07	271433	AGAP1-IT1
rs72981179	2	235777749	T	C	0.296	0.161	0.032	3.06E-07	271997	AGAP1-IT1
rs55961214	2	235778520	A	G	0.265	0.168	0.033	2.45E-07	272768	AGAP1-IT1
rs61402262	2	235778549	T	C	0.296	0.161	0.032	3.06E-07	272797	AGAP1-IT1
rs12470717	2	235778877	A	G	0.296	0.161	0.032	3.06E-07	273125	AGAP1-IT1
rs12463796	2	235779111	T	C	0.296	0.161	0.032	3.06E-07	273359	AGAP1-IT1
rs13400202	2	235780146	G	A	0.296	0.161	0.032	3.06E-07	274394	AGAP1-IT1
rs12472838	2	235780401	A	G	0.296	0.161	0.032	3.06E-07	274649	AGAP1-IT1
rs12470048	2	235780439	G	T	0.296	0.161	0.032	3.06E-07	274687	AGAP1-IT1
rs60591163	2	235781589	C	T	0.296	0.161	0.032	3.06E-07	275837	AGAP1-IT1
rs72981189	2	235781867	A	G	0.296	0.161	0.032	3.06E-07	276115	AGAP1-IT1
rs963846	2	235783052	G	C	0.684	-0.150	0.031	1.69E-06	277300	AGAP1-IT1
rs373964138	2	235783214	A	T	0.296	0.161	0.032	3.06E-07	277462	AGAP1-IT1
rs1878154	2	235783338	G	A	0.296	0.161	0.032	3.06E-07	277586	AGAP1-IT1
rs12466588	2	235783594	G	A	0.296	0.161	0.032	3.06E-07	277842	AGAP1-IT1
rs72981192	2	235783821	T	C	0.296	0.161	0.032	3.06E-07	278069	AGAP1-IT1
rs59832814	2	235785072	G	A	0.296	0.161	0.032	3.06E-07	279320	AGAP1-IT1
rs56009264	2	235785796	G	T	0.296	0.161	0.032	3.06E-07	280044	AGAP1-IT1
rs12469834	2	235786022	G	A	0.296	0.161	0.032	3.06E-07	280270	AGAP1-IT1

rs57957542	2	235786605	A	G	0.296	0.161	0.032	3.06E-07	280853	AGAP1-IT1
rs61499079	2	235786797	G	C	0.296	0.161	0.032	3.06E-07	281045	AGAP1-IT1
rs1878155	2	235787192	T	G	0.296	0.161	0.032	3.06E-07	281440	AGAP1-IT1
rs12472982	2	235788018	C	T	0.296	0.161	0.032	3.06E-07	282266	AGAP1-IT1
rs12472150	2	235788047	G	A	0.296	0.161	0.032	3.06E-07	282295	AGAP1-IT1
rs56329604	2	235788220	T	C	0.296	0.161	0.032	3.06E-07	282468	AGAP1-IT1
rs72983003	2	235788415	T	G	0.296	0.161	0.032	3.06E-07	282663	AGAP1-IT1
rs72983005	2	235788471	C	T	0.296	0.161	0.032	3.06E-07	282719	AGAP1-IT1
rs72983007	2	235788621	G	T	0.296	0.161	0.032	3.06E-07	282869	AGAP1-IT1
rs72983009	2	235788684	G	A	0.296	0.161	0.032	3.06E-07	282932	AGAP1-IT1
rs72983012	2	235788851	T	C	0.296	0.161	0.032	3.06E-07	283099	AGAP1-IT1
rs12469161	2	235789283	G	C	0.296	0.161	0.032	3.06E-07	283531	AGAP1-IT1
rs12469185	2	235789305	T	C	0.296	0.161	0.032	3.06E-07	283553	AGAP1-IT1
rs12474276	2	235789568	A	T	0.296	0.161	0.032	3.06E-07	283816	AGAP1-IT1
rs56062711	2	235789772	T	G	0.296	0.161	0.032	3.06E-07	284020	AGAP1-IT1
rs55936237	2	235789838	T	C	0.296	0.161	0.032	3.06E-07	284086	AGAP1-IT1
rs72983019	2	235790229	C	T	0.296	0.161	0.032	3.06E-07	284477	AGAP1-IT1
rs12475524	2	235790749	G	T	0.296	0.161	0.032	3.06E-07	284997	AGAP1-IT1
rs12474606	2	235790763	G	A	0.296	0.161	0.032	3.06E-07	285011	AGAP1-IT1
rs12470496	2	235790785	T	C	0.296	0.161	0.032	3.06E-07	285033	AGAP1-IT1
rs12474630	2	235790852	G	A	0.306	0.161	0.032	4.11E-07	285100	AGAP1-IT1
rs55881123	2	235790935	C	T	0.296	0.161	0.032	3.06E-07	285183	AGAP1-IT1
rs72983030	2	235791262	G	A	0.296	0.161	0.032	3.06E-07	285510	AGAP1-IT1
rs72983032	2	235791271	C	T	0.296	0.161	0.032	3.06E-07	285519	AGAP1-IT1
rs6845244	4	14785233	C	A	0.806	-0.177	0.038	2.74E-06	102937	LINC00504
rs4698291	4	14788174	G	C	0.806	-0.177	0.038	2.74E-06	99996	LINC00504
rs13151601	4	27958744	T	A	0.724	0.170	0.038	6.30E-06	741264	LINC02261
rs7665009	4	27960206	T	C	0.724	0.170	0.038	6.30E-06	742726	LINC02261
rs7438137	4	75831883	T	C	0.439	0.145	0.032	5.02E-06	70689	PPEF2
rs62326190	4	155956020	C	T	0.194	0.176	0.038	3.24E-06	197027	GUCY1B1
rs4306914	4	156020957	G	C	0.194	0.176	0.038	3.24E-06	261964	GUCY1B1
rs371103786	4	181401805	T	C	0.286	0.183	0.040	4.71E-06	-242655	LINC00290
rs4113603	4	184286837	C	T	0.347	-0.170	0.037	4.85E-06	-65606	ENPP6
rs1217971	4	184288728	T	C	0.622	0.180	0.038	2.63E-06	65250	LINC02363
rs1217972	4	184288886	G	T	0.622	0.180	0.038	2.63E-06	65092	LINC02363
rs6453850	6	75937483	A	G	0.306	0.170	0.036	1.84E-06	135196	IMPG1
rs9341546	6	75937859	T	C	0.653	-0.157	0.033	2.49E-06	134820	IMPG1
rs9350607	6	75937890	C	T	0.653	-0.157	0.033	2.49E-06	134789	IMPG1
rs9360962	6	75938923	G	T	0.663	-0.165	0.035	1.85E-06	133756	IMPG1
rs9343338	6	75939209	T	A	0.663	-0.165	0.035	1.85E-06	133470	IMPG1
rs13207399	6	75939355	G	C	0.306	0.170	0.036	1.84E-06	133324	IMPG1
rs2313752	6	75940342	C	T	0.663	-0.165	0.035	1.85E-06	132337	IMPG1
rs2313753	6	75942546	G	C	0.663	-0.165	0.035	1.85E-06	130133	IMPG1
rs2313755	6	75943204	T	C	0.673	-0.173	0.033	2.03E-07	129475	IMPG1
rs1610336	6	75943378	A	G	0.306	0.170	0.036	1.84E-06	129301	IMPG1
rs76014128	6	78481469	C	T	0.163	0.209	0.044	2.46E-06	-386004	IRAK1BP1
rs76364903	6	78482432	A	G	0.184	0.199	0.044	5.69E-06	-385041	IRAK1BP1
rs11968517	6	78486543	C	G	0.163	0.209	0.044	2.46E-06	-380930	IRAK1BP1
rs997648	6	78488462	A	G	0.163	0.209	0.044	2.46E-06	-379011	IRAK1BP1
rs78808189	6	78489069	A	T	0.163	0.209	0.044	2.46E-06	-378404	IRAK1BP1
rs77972979	6	78489165	T	C	0.163	0.209	0.044	2.46E-06	-378308	IRAK1BP1
rs79700878	6	78489720	A	G	0.163	0.209	0.044	2.46E-06	-377753	IRAK1BP1
rs1407103	6	78491257	A	C	0.163	0.209	0.044	2.46E-06	-376216	IRAK1BP1
rs7773443	6	78494501	A	G	0.163	0.209	0.044	2.46E-06	-372972	IRAK1BP1
rs76167616	6	78494757	T	C	0.163	0.209	0.044	2.46E-06	-372716	IRAK1BP1
rs76312719	6	78495068	C	T	0.163	0.209	0.044	2.46E-06	-372405	IRAK1BP1
rs77974320	6	78495334	C	T	0.163	0.209	0.044	2.46E-06	-372139	IRAK1BP1

rs148823079	6	78495409	C	T	0.163	0.209	0.044	2.46E-06	-372064	IRAK1BP1
rs114251753	6	78495612	A	G	0.163	0.209	0.044	2.46E-06	-371861	IRAK1BP1
rs80099930	6	78495713	C	T	0.163	0.209	0.044	2.46E-06	-371760	IRAK1BP1
rs115355509	6	78495767	G	A	0.163	0.209	0.044	2.46E-06	-371706	IRAK1BP1
rs200194409	6	78495969	A	C	0.163	0.209	0.044	2.46E-06	-371504	IRAK1BP1
rs11961883	6	78496288	A	G	0.163	0.209	0.044	2.46E-06	-371185	IRAK1BP1
rs7751932	6	78497915	A	C	0.163	0.209	0.044	2.46E-06	-369558	IRAK1BP1
rs79949695	6	78499776	C	G	0.163	0.209	0.044	2.46E-06	-367697	IRAK1BP1
rs7744184	6	78501326	A	G	0.163	0.209	0.044	2.46E-06	-366147	IRAK1BP1
rs79313931	6	78504256	A	G	0.163	0.209	0.044	2.46E-06	-363217	IRAK1BP1
rs6900179	6	78507704	C	G	0.163	0.209	0.044	2.46E-06	-359769	IRAK1BP1
rs6922709	6	78507743	C	T	0.163	0.209	0.044	2.46E-06	-359730	IRAK1BP1
rs1884966	6	78508721	A	G	0.163	0.209	0.044	2.46E-06	-358752	IRAK1BP1
rs60520348	6	78510501	G	T	0.163	0.209	0.044	2.46E-06	-356972	IRAK1BP1
rs11967020	6	78514356	T	C	0.163	0.209	0.044	2.46E-06	-353117	IRAK1BP1
rs1358817	6	78518080	A	C	0.163	0.209	0.044	2.46E-06	-349393	IRAK1BP1
rs723437	6	78521437	G	A	0.163	0.209	0.044	2.46E-06	-346036	IRAK1BP1
rs9359328	6	78524372	A	G	0.163	0.209	0.044	2.46E-06	-343101	IRAK1BP1
rs9343777	6	78525400	G	T	0.163	0.209	0.044	2.46E-06	-342073	IRAK1BP1
rs1321597	6	78527929	T	C	0.163	0.209	0.044	2.46E-06	-339544	IRAK1BP1
rs76641149	6	78541449	A	C	0.143	0.237	0.043	4.60E-08	-326024	IRAK1BP1
rs79747327	6	78556991	G	A	0.133	0.221	0.046	1.88E-06	-310482	IRAK1BP1
rs76889818	6	78566330	A	G	0.133	0.221	0.046	1.88E-06	-301143	IRAK1BP1
rs77413528	6	78581094	G	A	0.133	0.221	0.046	1.88E-06	-286379	IRAK1BP1
rs9359652	6	85344324	A	G	0.418	0.151	0.034	8.55E-06	45863	LINC02535
rs12201128	6	85353689	G	T	0.418	0.151	0.034	8.55E-06	36498	LINC02535
rs11771342	7	46204239	T	G	0.163	0.216	0.044	7.61E-07	-282364	IGFBP3
rs9691259	7	46206982	G	A	0.163	0.216	0.044	7.61E-07	-285107	IGFBP3
rs2633359	7	138122716	C	T	0.888	-0.250	0.055	4.64E-06	-1043	MIR4468
rs2440798	7	138127010	T	C	0.888	-0.250	0.055	4.64E-06	3251	MIR4468
rs4368897	7	146986807	T	C	0.153	0.205	0.046	6.51E-06	110803	CNTNAP2-AS1
rs12682543	8	29222041	A	G	0.643	-0.153	0.034	8.36E-06	41084	KIF13B
rs13263888	8	29223321	C	G	0.643	-0.153	0.034	8.36E-06	39804	KIF13B
rs4585791	9	614530	A	T	0.235	-0.197	0.042	2.68E-06	144238	KANK1
rs11812737	10	62669559	A	G	0.173	0.200	0.040	5.15E-07	-135299	ADO
rs4746518	10	62669835	G	A	0.214	0.171	0.038	6.35E-06	-135023	ADO
rs729738	10	62670505	A	C	0.214	0.171	0.038	6.35E-06	-134353	ADO
rs729739	10	62670542	A	G	0.214	0.171	0.038	6.35E-06	-134316	ADO
rs7394165	10	62677258	T	C	0.173	0.200	0.040	5.15E-07	-127600	ADO
rs7393302	10	62677294	G	T	0.214	0.171	0.038	6.35E-06	-127564	ADO
rs4746524	10	62678037	T	C	0.173	0.200	0.040	5.15E-07	-126821	ADO
rs224121	10	62687592	C	A	0.827	-0.216	0.043	3.96E-07	-117266	ADO
rs2960666	10	115819198	G	A	0.673	-0.156	0.035	8.81E-06	454270	GFRA1
rs372086626	11	39730859	C	T	0.173	0.208	0.044	1.73E-06	1082000	LINC01493
rs11063601	12	562434	G	T	0.112	0.248	0.055	6.25E-06	24053	LOC105369595
rs3217810	12	4279105	T	C	0.153	0.205	0.046	6.45E-06	-2920	CCND2-AS1
rs2075267	12	14962167	G	T	0.612	-0.150	0.032	2.84E-06	-438	ARHGDIB
rs11056258	12	14964465	T	G	0.592	-0.156	0.033	1.70E-06	-2736	ARHGDIB
rs7311989	12	14964603	A	C	0.592	-0.156	0.033	1.70E-06	-2874	ARHGDIB
rs7300467	12	14964663	G	T	0.602	-0.148	0.033	6.58E-06	-2934	ARHGDIB
rs10846097	12	14970137	T	C	0.643	-0.161	0.036	7.28E-06	-2886	PDE6H
rs7978415	12	14970534	A	G	0.592	-0.156	0.033	1.70E-06	-2489	PDE6H
rs3983696	12	14970938	T	C	0.592	-0.156	0.033	1.70E-06	-2085	PDE6H
rs7313555	12	14988129	C	T	0.663	-0.158	0.035	8.00E-06	-13705	LINC01489
rs11058862	12	122592803	T	C	0.398	0.141	0.032	7.77E-06	65556	KNTC1
rs11611668	12	122609181	T	C	0.398	0.143	0.031	4.87E-06	81934	KNTC1
rs61947369	13	25920538	A	G	0.163	0.186	0.042	9.46E-06	130494	SHISA2

rs9548368	13	38482144	T	C	0.367	0.151	0.033	4.62E-06	63156	LINC00437
rs9532237	13	38482716	A	G	0.367	0.151	0.033	4.62E-06	62584	LINC00437
rs6571996	14	40299024	A	C	0.745	-0.184	0.036	3.61E-07	-655875	LINC02315
rs7160617	14	40326534	G	A	0.806	-0.217	0.047	3.19E-06	-628365	LINC02315
rs6494413	15	63564443	A	G	0.755	-0.161	0.036	6.55E-06	-32911	FBXL22
rs72625754	15	63564562	C	T	0.173	0.207	0.039	1.20E-07	-32792	FBXL22
rs6494414	15	63566507	G	A	0.755	-0.161	0.036	6.55E-06	-30847	FBXL22
rs6494415	15	63570130	T	G	0.755	-0.161	0.036	6.55E-06	-27224	FBXL22
rs8028681	15	63572006	T	C	0.755	-0.161	0.036	6.55E-06	-25348	FBXL22
rs8029310	15	63572221	T	G	0.755	-0.161	0.036	6.55E-06	-25133	FBXL22
rs7183892	15	63586733	T	C	0.755	-0.161	0.036	6.55E-06	-10621	FBXL22
rs7168622	15	63668675	A	C	0.122	0.230	0.047	9.81E-07	-67085	USP3-AS1
rs7182375	15	63813431	G	A	0.776	-0.163	0.036	4.85E-06	20512	HERC1
rs7170689	15	63831046	T	C	0.776	-0.159	0.036	9.59E-06	2897	HERC1
rs8042418	15	63832686	A	G	0.776	-0.159	0.036	9.59E-06	1257	HERC1
rs7170433	15	63837452	C	T	0.776	-0.159	0.036	9.59E-06	-3509	HERC1
rs214239	16	303625	A	G	0.214	0.182	0.040	5.50E-06	20472	PDIA2
rs1558346	16	6514333	T	C	0.143	0.208	0.046	7.22E-06	881865	MIR8065
rs72804582	16	86720970	G	A	0.112	0.246	0.055	8.44E-06	985	LINC02189
rs11659939	18	33845019	A	G	0.214	0.179	0.040	9.37E-06	266403	ASXL3
rs1051651	19	7116272	C	G	0.245	0.166	0.035	2.23E-06	46827	ZNF557
rs11670340	19	7436914	A	G	0.408	0.138	0.031	7.47E-06	54079	ARHGEF18
rs6139617	20	5063227	G	A	0.469	-0.143	0.031	2.93E-06	49877	TMEM230

Table S6. Annotation of variants associated with GO_ATP_METABOLIC level with p-val$<10^{-5}$.

rsID	CHR	POS	A1	A2	AF1	BETA	SE	P	distancetoFeature	symbol
rs4649064	1	25094616	G	A	0.456	0.232	0.045	2.07E-07	71112	RUNX1
rs6657249	1	25095126	C	A	0.395	0.250	0.044	1.65E-08	71622	RUNX1
rs11588172	1	25095499	C	T	0.395	0.250	0.044	1.65E-08	71995	RUNX1
rs56365408	1	25096844	G	A	0.395	0.250	0.044	1.65E-08	73340	RUNX1
rs56348607	1	25097067	G	A	0.412	0.258	0.045	1.23E-08	73563	RUNX1
rs58693652	1	25097185	A	G	0.395	0.250	0.044	1.65E-08	73681	RUNX1
rs2536363	1	83382963	C	T	0.728	-0.254	0.056	6.46E-06	-396754	LINC01361
rs2536361	1	83383171	T	G	0.728	-0.254	0.056	6.46E-06	-396962	LINC01361
N.A	1	121043250	C	G	0.728	-0.201	0.041	1.25E-06	-44096	FCGR1B
rs12471349	2	231630391	A	C	0.456	-0.203	0.043	2.12E-06	37489	TEX44
rs11686124	2	231631051	A	G	0.447	-0.200	0.042	2.28E-06	38149	TEX44
rs12619551	2	231632587	T	C	0.447	-0.200	0.042	2.28E-06	39685	TEX44
rs12621259	2	231633982	T	C	0.447	-0.200	0.042	2.28E-06	41080	TEX44
rs10204481	2	231635421	T	C	0.456	-0.203	0.043	2.12E-06	42519	TEX44
rs10204963	2	231635773	A	G	0.456	-0.203	0.043	2.12E-06	42871	TEX44
rs11685588	2	231635997	A	C	0.456	-0.203	0.043	2.12E-06	43095	TEX44
rs931390	3	4736178	T	C	0.746	-0.269	0.051	1.26E-07	15413	EGOT
rs4690284	4	73616	C	T	0.105	0.315	0.071	9.38E-06	20330	ZNF595
rs11735004	4	8519493	A	G	0.105	0.359	0.077	3.52E-06	-58833	CPZ
rs28520722	4	168372678	C	G	0.158	0.261	0.055	2.56E-06	-53870	DDX60
rs10474554	5	78503335	T	C	0.816	-0.256	0.054	2.01E-06	142751	SCAMP1
rs10474555	5	78503370	T	C	0.816	-0.256	0.054	2.01E-06	142786	SCAMP1
rs12522528	5	172824478	C	A	0.272	0.247	0.049	5.68E-07	-9798	ERGIC1
rs6891132	5	172824828	G	A	0.711	-0.224	0.049	4.38E-06	-9448	ERGIC1
rs4867689	5	172825091	C	T	0.711	-0.224	0.049	4.38E-06	-9185	ERGIC1
rs4867690	5	172825370	G	A	0.711	-0.224	0.049	4.38E-06	-8906	ERGIC1
rs4868216	5	172825449	A	G	0.711	-0.224	0.049	4.38E-06	-8827	ERGIC1
rs4867691	5	172825540	G	C	0.711	-0.224	0.049	4.38E-06	-8736	ERGIC1
rs7717831	5	172825774	C	T	0.711	-0.224	0.049	4.38E-06	-8502	ERGIC1
rs7717986	5	172825824	A	T	0.711	-0.224	0.049	4.38E-06	-8452	ERGIC1
rs7736149	5	172825871	A	G	0.711	-0.224	0.049	4.38E-06	-8405	ERGIC1
rs7736270	5	172825917	A	G	0.711	-0.224	0.049	4.38E-06	-8359	ERGIC1
rs792994	5	172826179	T	C	0.272	0.247	0.049	5.68E-07	-8097	ERGIC1
rs11748400	5	172826590	G	T	0.263	0.255	0.049	1.53E-07	-7686	ERGIC1
rs4868217	5	172826780	C	T	0.711	-0.224	0.049	4.38E-06	-7496	ERGIC1
rs12519115	5	172827357	T	C	0.254	0.259	0.048	7.76E-08	-6919	ERGIC1
rs7707991	5	172827470	G	A	0.711	-0.224	0.049	4.38E-06	-6806	ERGIC1
rs6556047	5	172828846	A	T	0.702	-0.211	0.047	8.90E-06	-5430	ERGIC1
rs1564259	5	172829021	C	T	0.702	-0.211	0.047	8.90E-06	-5255	ERGIC1
rs1006721	5	172829068	A	G	0.702	-0.211	0.047	8.90E-06	-5208	ERGIC1
rs10057822	5	172829748	G	A	0.702	-0.211	0.047	8.90E-06	-4528	ERGIC1
rs2339652	5	172830696	G	A	0.702	-0.211	0.047	8.90E-06	-3580	ERGIC1
rs4868218	5	172830968	A	C	0.702	-0.211	0.047	8.90E-06	-3308	ERGIC1
rs35566639	5	172834459	A	G	0.254	0.250	0.049	3.70E-07	183	ERGIC1
rs35768025	5	172834486	A	C	0.254	0.250	0.049	3.70E-07	210	ERGIC1
rs78078909	5	172834535	A	C	0.254	0.250	0.049	3.70E-07	259	ERGIC1
rs793010	5	172835670	T	C	0.263	0.241	0.050	1.31E-06	1394	ERGIC1
rs6895746	5	172838337	G	A	0.254	0.263	0.048	3.69E-08	4061	ERGIC1
rs11134762	5	172838397	A	G	0.237	0.267	0.047	1.87E-08	4121	ERGIC1

rs11949146	5	172840207	A	G	0.219	0.282	0.050	1.54E-08	5931	ERGIC1
rs6877332	5	172841627	A	G	0.246	0.267	0.051	1.61E-07	7351	ERGIC1
rs7722970	5	172842868	T	G	0.219	0.258	0.052	8.18E-07	8592	ERGIC1
rs10945708	6	161017963	T	C	0.298	0.227	0.049	3.32E-06	26235	MAP3K4
rs12195182	6	161108526	C	G	0.465	0.195	0.042	4.49E-06	53457	AGPAT4-IT1
rs2314157	6	161190126	C	G	0.439	0.194	0.042	3.99E-06	-28143	AGPAT4-IT1
rs3735487	7	45065547	G	A	0.114	0.274	0.060	4.18E-06	23368	NACAD
rs2140953	7	106848675	C	A	0.105	0.354	0.078	5.32E-06	-16604	PIK3CG
rs7791674	7	106849681	T	C	0.105	0.354	0.078	5.32E-06	-15598	PIK3CG
rs35031873	7	142758607	C	A	0.079	0.385	0.074	2.17E-07	-373471	PIP
rs1567924	8	19201161	G	C	0.237	0.244	0.053	4.21E-06	58309	LOC100128993
rs62524263	8	102126785	G	C	0.649	0.214	0.047	5.51E-06	1352	MIR5680
rs6982041	8	102127205	C	A	0.649	0.214	0.047	5.51E-06	1772	MIR5680
rs7819114	8	102128424	T	C	0.649	0.214	0.047	5.51E-06	2991	MIR5680
rs4734053	8	102129163	A	G	0.649	0.214	0.047	5.51E-06	3730	MIR5680
rs4734616	8	102129293	T	C	0.658	0.215	0.046	3.56E-06	3860	MIR5680
rs7826028	8	102131357	C	T	0.649	0.214	0.047	5.51E-06	5924	MIR5680
rs7000087	8	102131841	G	T	0.640	0.218	0.047	3.59E-06	6408	MIR5680
rs9942801	8	102132950	G	A	0.649	0.214	0.047	5.51E-06	7517	MIR5680
rs4734617	8	102138783	C	A	0.649	0.214	0.047	5.51E-06	13350	MIR5680
rs13248776	8	102145359	T	C	0.649	0.214	0.047	5.51E-06	19926	MIR5680
rs13250882	8	102145366	C	T	0.649	0.214	0.047	5.51E-06	19933	MIR5680
rs6988832	8	102146011	G	A	0.649	0.214	0.047	5.51E-06	20578	MIR5680
rs2105616	8	102146140	C	A	0.649	0.214	0.047	5.51E-06	20707	MIR5680
rs372431986	8	102146141	C	A	0.649	0.214	0.047	5.51E-06	20708	MIR5680
rs1892965	8	102146620	A	C	0.649	0.214	0.047	5.51E-06	21187	MIR5680
rs13268773	8	102147224	A	G	0.649	0.214	0.047	5.51E-06	21791	MIR5680
rs6468817	8	102148609	A	T	0.649	0.214	0.047	5.51E-06	23176	MIR5680
rs12681037	8	102149097	A	T	0.649	0.214	0.047	5.51E-06	23664	MIR5680
rs6468818	8	102149937	G	C	0.649	0.214	0.047	5.51E-06	24504	MIR5680
rs4291239	8	102150410	A	G	0.649	0.214	0.047	5.51E-06	24977	MIR5680
rs10093024	8	102151656	C	T	0.649	0.214	0.047	5.51E-06	26223	MIR5680
rs6992948	8	102152160	A	C	0.596	0.220	0.045	1.27E-06	26727	MIR5680
rs1111911	8	102152888	T	A	0.649	0.214	0.047	5.51E-06	27455	MIR5680
rs6980613	8	102153223	G	T	0.649	0.214	0.047	5.51E-06	27790	MIR5680
rs1573311	8	102154787	T	C	0.649	0.214	0.047	5.51E-06	29354	MIR5680
rs2387095	8	102155415	G	T	0.649	0.214	0.047	5.51E-06	29982	MIR5680
rs1547370	8	102155949	T	C	0.649	0.214	0.047	5.51E-06	30516	MIR5680
rs2186682	8	102157973	G	A	0.649	0.214	0.047	5.51E-06	32540	MIR5680
rs1573309	8	102160658	G	A	0.605	0.234	0.049	1.48E-06	35225	MIR5680
rs1148514	8	102168709	C	G	0.605	0.234	0.049	1.48E-06	43276	MIR5680
rs1148515	8	102168828	G	C	0.605	0.234	0.049	1.48E-06	43395	MIR5680
rs1148516	8	102168999	G	A	0.605	0.234	0.049	1.48E-06	43566	MIR5680
rs78848775	8	133734878	T	A	0.105	0.319	0.071	6.46E-06	42475	LOC101927798
rs77131790	8	133735142	G	A	0.105	0.319	0.071	6.46E-06	42211	LOC101927798
rs75149129	8	133735347	G	T	0.105	0.319	0.071	6.46E-06	42006	LOC101927798
rs76106243	8	133737227	C	T	0.105	0.319	0.071	6.46E-06	40126	LOC101927798
rs17820827	9	13863723	G	C	0.167	0.286	0.062	4.62E-06	-64249	LINC00583
rs7858214	9	13865261	T	C	0.105	0.327	0.070	3.09E-06	-62711	LINC00583
rs11259468	10	15081387	C	G	0.114	0.308	0.069	9.07E-06	7390	ACBD7

rs10901547	10	126174101	T	C	0.281	0.236	0.048	7.53E-07	-200974	FANK1-AS1
rs72826371	10	126177069	T	C	0.237	0.256	0.049	1.45E-07	-203942	FANK1-AS1
rs12356272	10	126177590	G	A	0.272	0.242	0.047	2.49E-07	-204463	FANK1-AS1
rs12357824	10	126180060	C	T	0.272	0.242	0.047	2.49E-07	-206933	FANK1-AS1
rs12766940	10	126180562	C	T	0.272	0.242	0.047	2.49E-07	-207435	FANK1-AS1
rs11244871	10	126183597	A	G	0.263	0.249	0.046	6.57E-08	204859	ADAM12
rs1459709	10	126184259	A	G	0.263	0.249	0.046	6.57E-08	204197	ADAM12
rs17683203	10	126186844	A	G	0.263	0.249	0.046	6.57E-08	201612	ADAM12
rs17745507	10	126187170	T	A	0.228	0.265	0.048	2.93E-08	201286	ADAM12
rs10829685	10	130058387	T	G	0.228	0.259	0.052	6.16E-07	52431	C10orf143
rs7082237	10	130091533	T	C	0.140	0.272	0.061	8.76E-06	19285	C10orf143
rs1945331	11	21242296	T	C	0.123	0.304	0.068	7.66E-06	572744	NELL1
rs4755920	11	44947205	A	G	0.360	-0.214	0.048	6.66E-06	4102	TP53I11
rs141434045	12	115147278	G	A	0.123	0.352	0.072	1.08E-06	-463113	TBX5-TBX3
rs10082870	12	115148523	C	G	0.123	0.352	0.072	1.08E-06	-464358	TBX5-TBX3
rs2270458	12	115151267	T	C	0.114	0.364	0.074	8.36E-07	-467102	TBX5-TBX3
rs12310526	12	115151871	A	G	0.114	0.364	0.074	8.36E-07	-467706	TBX5-TBX3
rs112088312	12	115154065	T	C	0.114	0.364	0.074	8.36E-07	-469900	TBX5-TBX3
rs11067442	12	115154219	T	C	0.114	0.364	0.074	8.36E-07	-470054	TBX5-TBX3
rs74846266	12	115154979	T	C	0.114	0.364	0.074	8.36E-07	-470814	TBX5-TBX3
rs7970502	12	115155723	T	C	0.114	0.364	0.074	8.36E-07	-471558	TBX5-TBX3
rs7145901	14	52220916	T	C	0.228	0.251	0.057	9.57E-06	-46798	PTGDR
rs71474496	15	42625943	G	A	0.123	0.318	0.067	1.90E-06	50283	STARD9
rs11638835	15	42626143	G	A	0.868	-0.301	0.066	5.41E-06	50483	STARD9
rs12911585	15	42642347	C	T	0.132	0.306	0.066	3.31E-06	66687	STARD9
rs61122145	15	42699689	A	G	0.149	0.306	0.063	1.12E-06	37438	CDAN1
rs12902830	15	42705184	T	C	0.140	0.320	0.063	3.93E-07	31943	CDAN1
rs35392023	15	42709462	A	G	0.149	0.311	0.062	6.02E-07	27665	CDAN1
rs1058846	15	42719826	T	C	0.149	0.311	0.062	6.02E-07	17301	CDAN1
rs17774047	15	42721028	T	C	0.149	0.311	0.062	6.02E-07	16099	CDAN1
rs35088691	15	42772565	C	T	0.140	0.328	0.062	1.39E-07	-35438	CDAN1
rs12913618	15	42779131	T	C	0.140	0.328	0.062	1.39E-07	-42004	CDAN1
rs12915116	15	42782482	T	C	0.140	0.328	0.062	1.39E-07	-45355	CDAN1
rs35298309	15	42784803	C	T	0.149	0.337	0.060	1.86E-08	-47676	CDAN1
rs12908349	15	42794029	T	C	0.123	0.315	0.067	2.59E-06	-56902	CDAN1
rs66932813	15	42796424	G	T	0.123	0.315	0.067	2.59E-06	-59297	CDAN1
rs36092914	15	42808425	A	C	0.123	0.315	0.067	2.59E-06	-71298	CDAN1
rs964776412	15	42817281	A	T	0.123	0.315	0.067	2.59E-06	-80154	CDAN1
rs72713784	15	42823738	G	A	0.123	0.315	0.067	2.59E-06	-86611	CDAN1
rs34745458	15	42845003	C	T	0.123	0.315	0.067	2.59E-06	75807	TTBK2
rs72713795	15	42849430	A	T	0.123	0.315	0.067	2.59E-06	71380	TTBK2
rs35202249	15	42855844	T	C	0.123	0.315	0.067	2.59E-06	64966	TTBK2
rs2683240	15	81120333	T	C	0.675	-0.209	0.046	5.84E-06	-62268	IL16
rs2460852	15	81123862	T	G	0.675	-0.209	0.046	5.84E-06	-58739	IL16
rs2683249	15	81129249	G	A	0.675	-0.209	0.046	5.84E-06	-53352	IL16
rs2683255	15	81134198	C	G	0.675	-0.209	0.046	5.84E-06	-48403	IL16
rs2683256	15	81135816	A	G	0.675	-0.209	0.046	5.84E-06	-46785	IL16
rs4404021	15	96060577	T	C	0.228	0.227	0.048	2.70E-06	-265362	NR2F2
rs7166802	15	96062205	T	G	0.237	0.219	0.049	7.62E-06	-263734	NR2F2
rs7166314	15	96062218	G	A	0.237	0.219	0.049	7.62E-06	-263721	NR2F2

rs79651776	16	11650396	G	A	0.140	0.273	0.061	7.50E-06	-14014	LITAF
rs8056041	16	83121479	A	G	0.263	0.240	0.053	6.68E-06	-131180	LOC101928417
rs8082569	17	62828928	G	A	0.491	0.198	0.045	9.81E-06	-20583	MARCHF10
rs12948372	17	62831387	A	G	0.412	0.211	0.047	6.96E-06	-23042	MARCHF10
rs35594211	17	62831620	G	A	0.412	0.211	0.047	6.96E-06	-23275	MARCHF10
rs11868614	17	62831835	A	G	0.412	0.211	0.047	6.96E-06	-23490	MARCHF10
rs35354702	17	62832130	G	A	0.412	0.211	0.047	6.96E-06	-23785	MARCHF10
rs35596881	17	62834618	G	A	0.412	0.211	0.047	6.96E-06	-26273	MARCHF10
rs8182298	17	62837616	C	T	0.439	0.208	0.043	1.08E-06	-29271	MARCHF10
rs8182316	17	62837746	T	C	0.456	0.207	0.042	1.13E-06	-29401	MARCHF10
rs8076190	17	62837908	G	A	0.456	0.207	0.042	1.13E-06	-29563	MARCHF10
rs8077260	17	62838375	C	G	0.456	0.207	0.042	1.13E-06	-30030	MARCHF10
rs8182324	17	62838599	G	A	0.456	0.207	0.042	1.13E-06	-30254	MARCHF10
rs6504130	17	62838901	T	C	0.421	0.215	0.043	4.92E-07	-30556	MARCHF10
rs8064710	17	62839334	C	T	0.439	0.208	0.043	1.08E-06	-30989	MARCHF10
rs8064402	17	62839705	A	G	0.439	0.208	0.043	1.08E-06	-31360	MARCHF10
rs8067960	17	62839798	T	C	0.439	0.208	0.043	1.08E-06	-31453	MARCHF10
rs7226368	17	62840771	C	G	0.421	0.215	0.043	4.92E-07	-32426	MARCHF10
rs12943672	17	62841241	A	T	0.439	0.208	0.043	1.08E-06	-32896	MARCHF10
rs1476811	17	62841947	T	C	0.421	0.215	0.043	4.92E-07	-33602	MARCHF10
rs1476812	17	62842115	T	C	0.421	0.215	0.043	4.92E-07	-33770	MARCHF10
rs1987647	17	62842528	C	T	0.377	0.216	0.046	2.90E-06	-34183	MARCHF10
rs12943559	17	62843136	T	C	0.421	0.215	0.043	4.92E-07	-34791	MARCHF10
rs9903891	17	62843788	A	T	0.447	0.213	0.043	8.16E-07	-35443	MARCHF10
rs12936567	17	62844192	A	G	0.447	0.213	0.043	8.16E-07	-35847	MARCHF10
rs11871628	17	62844788	C	T	0.447	0.213	0.043	8.16E-07	-36443	MARCHF10
rs35395730	17	62845085	C	A	0.447	0.213	0.043	8.16E-07	-36740	MARCHF10
rs55895685	17	62846360	C	G	0.439	0.214	0.044	1.50E-06	-38015	MARCHF10
rs55854611	17	62846443	G	T	0.439	0.214	0.044	1.50E-06	-38098	MARCHF10
rs55909719	17	62846445	A	G	0.404	0.210	0.044	1.85E-06	-38100	MARCHF10
rs8072675	17	62846458	G	A	0.439	0.214	0.044	1.50E-06	-38113	MARCHF10
rs34350332	17	62847043	C	T	0.439	0.214	0.044	1.50E-06	-38698	MARCHF10
rs34826284	17	62847771	T	C	0.404	0.230	0.044	2.15E-07	-39426	MARCHF10
rs35290531	17	62849438	A	C	0.439	0.214	0.044	1.50E-06	-41093	MARCHF10
rs35572014	17	62849602	C	T	0.447	0.213	0.045	2.75E-06	-41257	MARCHF10
rs12051594	17	62850242	A	G	0.456	0.212	0.044	1.49E-06	-41897	MARCHF10
rs9915826	17	62850877	T	C	0.421	0.215	0.045	1.71E-06	-42532	MARCHF10
rs9303464	17	62850993	C	T	0.447	0.213	0.045	2.75E-06	-42648	MARCHF10
rs9890310	17	62851050	G	T	0.447	0.213	0.045	2.75E-06	-42705	MARCHF10
rs9890570	17	62851197	C	T	0.421	0.215	0.045	1.71E-06	-42852	MARCHF10
rs8076646	17	62852550	T	C	0.386	0.209	0.045	3.44E-06	-44205	MARCHF10
rs8081770	17	62852855	C	T	0.465	0.217	0.047	3.98E-06	-44510	MARCHF10
rs61638683	17	82849592	T	G	0.535	-0.210	0.045	3.38E-06	-9013	ZNF750
rs59300995	17	82849594	C	G	0.553	-0.206	0.044	2.47E-06	-9015	ZNF750
rs7216324	17	82851589	C	A	0.535	-0.210	0.045	3.38E-06	-11010	ZNF750
rs4986118	17	82851689	C	G	0.447	0.206	0.044	2.47E-06	-11110	ZNF750
rs7215939	17	82851732	G	C	0.535	-0.210	0.045	3.38E-06	-11153	ZNF750
rs7214952	17	82851853	T	G	0.535	-0.210	0.045	3.38E-06	-11274	ZNF750
rs11077948	17	82852057	T	C	0.553	-0.206	0.044	2.47E-06	-11478	ZNF750
rs9913679	17	82855022	G	A	0.535	-0.210	0.045	3.38E-06	-14443	ZNF750

rs12600507	17	82856293	A	G	0.553	-0.206	0.044	2.47E-06	-15714	ZNF750
rs9303013	17	82857421	C	T	0.544	-0.212	0.046	4.73E-06	-16842	ZNF750
rs7220732	17	82857901	C	A	0.544	-0.212	0.046	4.73E-06	-17322	ZNF750
rs7209378	17	82859192	A	G	0.561	-0.208	0.045	3.36E-06	-18613	ZNF750
rs9890196	17	82860297	C	T	0.553	-0.213	0.045	2.58E-06	-19718	ZNF750
rs6502007	17	82861401	G	A	0.544	-0.196	0.043	6.08E-06	-20822	ZNF750
rs8082007	17	82862130	A	G	0.526	-0.210	0.046	5.71E-06	-21551	ZNF750
rs8065192	17	82862158	G	A	0.553	-0.206	0.044	2.47E-06	-21579	ZNF750
rs9303014	17	82866493	T	G	0.526	-0.210	0.046	5.71E-06	-25914	ZNF750
rs4986120	17	82866778	G	T	0.535	-0.204	0.046	7.70E-06	-26199	ZNF750
rs7224427	17	82867331	A	G	0.526	-0.210	0.046	5.71E-06	-26752	ZNF750
rs56353584	17	82869089	G	T	0.553	-0.207	0.046	6.21E-06	-28510	ZNF750
rs56322462	17	82869216	T	C	0.544	-0.212	0.046	4.73E-06	-28637	ZNF750
rs66893622	17	82869369	T	A	0.535	-0.212	0.047	8.08E-06	-28790	ZNF750
rs9892064	17	82870027	T	C	0.544	-0.212	0.046	4.73E-06	-29448	ZNF750
rs3744161	17	82870181	A	G	0.535	-0.212	0.047	8.08E-06	-29602	ZNF750
rs6502008	17	82872389	A	G	0.544	-0.212	0.046	4.73E-06	-31810	ZNF750
rs6502009	17	82872499	T	C	0.544	-0.212	0.046	4.73E-06	-31920	ZNF750
rs11655504	17	82873448	C	T	0.535	-0.212	0.047	8.08E-06	-32869	ZNF750
rs898093	17	82874591	T	C	0.535	-0.212	0.047	8.08E-06	-34012	ZNF750
rs4986123	17	82876370	T	C	0.544	-0.205	0.045	4.52E-06	-35791	ZNF750
rs8075190	17	82878558	T	C	0.544	-0.201	0.045	8.17E-06	-37979	ZNF750
rs9747095	17	82882712	G	A	0.474	0.204	0.044	4.30E-06	-42133	ZNF750
rs7221830	17	82890938	G	T	0.474	0.204	0.044	4.30E-06	-50359	ZNF750
rs59103510	17	82891072	T	G	0.518	-0.204	0.045	7.08E-06	-50493	ZNF750
rs12948880	17	82893224	C	T	0.474	0.210	0.046	5.71E-06	-52645	ZNF750
rs4986130	17	82895802	C	T	0.482	0.204	0.045	7.08E-06	-55223	ZNF750
rs4986131	17	82895920	G	A	0.482	0.204	0.045	7.08E-06	-55341	ZNF750
rs12951357	17	82896533	A	G	0.474	0.210	0.046	5.71E-06	-55954	ZNF750
rs1809252	17	82897473	A	G	0.518	-0.204	0.045	7.08E-06	-56894	ZNF750
rs939262	17	82897656	G	C	0.518	-0.204	0.045	7.08E-06	-57077	ZNF750
rs9891862	17	82900793	A	G	0.518	-0.204	0.045	7.08E-06	-60214	ZNF750
rs4986134	17	82901278	A	G	0.518	-0.204	0.045	7.08E-06	-60699	ZNF750
rs2292971	17	82905981	C	T	0.482	0.204	0.045	7.08E-06	-65402	ZNF750
rs8074371	17	82908853	C	G	0.482	0.204	0.045	7.08E-06	-68274	ZNF750
rs12947897	17	82913585	G	A	0.474	0.204	0.044	4.16E-06	-73006	ZNF750
rs8064687	17	82915798	C	G	0.482	0.204	0.045	7.08E-06	-75219	ZNF750
rs8069773	17	82916667	T	G	0.474	0.204	0.044	4.16E-06	-76088	ZNF750
rs73007122	19	10004217	T	C	0.105	0.338	0.069	1.08E-06	6255	COL5A3
rs35143140	19	10005321	A	G	0.289	0.218	0.047	2.95E-06	5151	COL5A3
rs11085530	19	10006027	G	A	0.289	0.218	0.047	2.95E-06	4445	COL5A3
rs35678764	19	10006948	A	C	0.289	0.218	0.047	2.95E-06	3524	COL5A3
rs9797822	19	10007058	A	C	0.289	0.218	0.047	2.95E-06	3414	COL5A3
rs9797907	19	10007075	G	T	0.289	0.218	0.047	2.95E-06	3397	COL5A3
rs11672609	19	58495557	T	C	0.140	0.258	0.057	6.77E-06	16509	SLC27A5
rs11672614	19	58495617	T	C	0.140	0.258	0.057	6.77E-06	16449	SLC27A5
rs11672730	19	58495836	A	G	0.140	0.258	0.057	6.77E-06	16230	SLC27A5
rs55652736	19	58496199	T	G	0.140	0.258	0.057	6.77E-06	15867	SLC27A5
rs73066226	19	58496846	C	T	0.149	0.256	0.057	6.21E-06	15220	SLC27A5
rs11671092	19	58497742	C	T	0.149	0.256	0.057	6.21E-06	14324	SLC27A5

rs55928441	19	58498307	C	T	0.140	0.258	0.057	6.77E-06	13759	SLC27A5
rs73066228	19	58501829	G	A	0.140	0.258	0.057	7.22E-06	10237	SLC27A5
rs4810126	20	58244817	T	C	0.167	0.298	0.061	1.23E-06	-16163	ANKRD60
rs76912023	20	58257517	T	C	0.158	0.300	0.062	1.39E-06	-28863	ANKRD60
rs4811976	20	58259149	A	G	0.158	0.300	0.062	1.39E-06	-30495	ANKRD60
rs563489	20	58355773	G	A	0.649	-0.237	0.051	3.08E-06	-33350	VAPB
rs71315381	22	17510895	G	C	0.140	0.274	0.061	6.67E-06	-52545	SLC25A18
rs2142664	22	46559201	G	A	0.237	0.227	0.051	8.47E-06	-16812	GRAMD4

Table S7. Annotation of variants associated with *GATA4* activity level with p-val<10^{-5}.

Tags	Chrom	Position	Ref_Base	Alt_Base	strand	Coding	Gene	Sequence_Ontology	cDNA_change	Protein	Driver	CScape_pred
chr1:g.3681352G>T	chr1	3681352	G	T	+		TP73	intron_variant	c.-33-981G>T			driver
chr1:g.14161090C>T	chr1	14161090	C	T	+		KAZN	intron_variant	c.92-19345C>T			passenger
chr1:g.15541637A>C	chr1	15541637	A	C	+		DNAJC16	intron_variant	c.575-2762A>C			passenger
chr1:g.30722679C>T	chr1	30722679	C	T	+		MATN1	intron_variant	c.94+779G>A			driver
chr1:g.35306972T>A	chr1	35306972	T	A	+		ZMYM4	intron_variant	c.40-18388T>A			driver
chr1:g.44495436G>A	chr1	44495436	G	A	+		RNF220	intron_variant	c.625+82714G>A			driver
chr1:g.45958363G>T	chr1	45958363	G	T	+		MAST2	intron_variant	c.501-1023G>T			driver
chr1:g.51680266A>T	chr1	51680266	A	T	+		OSBPL9	intron_variant	c.241+10754A>T			driver
chr1:g.66551873T>C	chr1	66551873	T	C	+		SGIP1	intron_variant	c.10+17505T>C			passenger
chr1:g.76475386T>G	chr1	76475386	T	G	+		ST6GALNAC3	intron_variant	c.623+62969T>G			driver
chr1:g.76934820G>A	chr1	76934820	G	A	+		ST6GALNAC5	intron_variant	c.261+66078G>A			driver
chr1:g.81962684G>T	chr1	81962684	G	T	+		ADGRL2	intron_variant	c.2006-3374G>T			driver
chr1:g.100489433C>T	chr1	100489433	C	T	+		CDC14A	intron_variant	c.1137+4982C>T			driver
chr1:g.109273131C>G	chr1	109273131	C	G	+		CELSR2	intron_variant	c.8339-35C>G			driver
chr1:g.115979836T>A	chr1	115979836	T	A	+		SLC22A15	intron_variant	c.87+3122T>A			driver
chr1:g.150375962C>G	chr1	150375962	C	G	+		RPRD2	intron_variant	c.205+11043C>G			driver
chr1:g.154250525G>T	chr1	154250525	G	T	+		UBAP2L	intron_variant	c.1214-516G>T			driver
chr1:g.155183439G>T	chr1	155183439	G	T	+		TRIM46	intron_variant	c.1887-358G>T			driver
chr1:g.155278125G>T	chr1	155278125	G	T	+		HCN3	intron_variant	c.278+257G>T			driver
chr1:g.157694861C>G	chr1	157694861	C	G	+		FCRL3	intron_variant	c.1411+468G>C			passenger
chr1:g.161161206G>A	chr1	161161206	G	A	+	Yes	USP21	missense_variant	c.566G>A	p.Arg189Gln		driver
chr1:g.162640635A>G	chr1	162640635	A	G	+		DDR2	intron_variant	c.-192+8004A>G		Oncogene	passenger
chr1:g.169711411G>A	chr1	169711411	G	A	+		SELL	intron_variant	c.3+85C>T			driver
chr1:g.176766253C>A	chr1	176766253	C	A	+		PAPPA2	intron_variant	c.4323+416C>A			driver
chr1:g.178211277T>C	chr1	178211277	T	C	+		RASAL2	intron_variant	c.203-72287T>C			passenger
chr1:g.178473177C>G	chr1	178473177	C	G	+	Yes	RASAL2	missense_variant	c.3781C>G	p.Pro1261Ala		Damaging
chr1:g.180074023T>G	chr1	180074023	T	G	+		CEP350	intron_variant	c.5568-999T>G			driver
chr1:g.185941653A>G	chr1	185941653	A	G	+		HMCN1	intron_variant	c.1828+7829A>G			passenger
chr1:g.186075049C>G	chr1	186075049	C	G	+		HMCN1	intron_variant	c.8290+158C>G			driver
chr1:g.186935924A>C	chr1	186935924	A	C	+		PLA2G4A	intron_variant	c.695+3025A>C			driver
chr1:g.188041799A>T	chr1	188041799	A	T	+		AL136372.2	lnc_RNA				driver
chr1:g.201578348C>G	chr1	201578348	C	G	+							driver
chr1:g.209758085C>T	chr1	209758085	C	T	+		TRAF3IP3	intron_variant	c.-159-949C>T			driver
chr1:g.210010348C>G	chr1	210010348	C	G	+		SYT14	intron_variant	c.-485-3285C>G			driver
chr1:g.220576354C>A	chr1	220576354	C	A	+		MARK1	intron_variant	c.52-3000C>A			driver
chr1:g.228017313T>A	chr1	228017313	T	A	+		WNT3A	intron_variant	c.72-5354T>A			driver
chr1:g.237459319T>C	chr1	237459319	T	C	+		RYR2	intron_variant	c.1612+2584T>C			driver
chr1:g.240028568C>A	chr1	240028568	C	A	+							driver
chr1:g.245447455A>G	chr1	245447455	A	G	+		KIF26B	intron_variant	c.1166+27710A>G			passenger
chr2:g.1188021G>A	chr2	1188021	G	A	+		SNTG2	intron_variant	c.591+14838G>A			passenger
chr2:g.9432457C>T	chr2	9432457	C	T	+		CPSF3	intron_variant	c.342-54C>T			driver
chr2:g.16074161T>G	chr2	16074161	T	G	+		GACAT3	lnc_RNA				driver
chr2:g.24702669A>T	chr2	24702669	A	T	+		NCOA1	intron_variant	c.950-2417A>T		fusion	driver
chr2:g.29156465G>A	chr2	29156465	G	A	+		CLIP4	intron_variant	c.1255+22G>A			driver
chr2:g.32215682T>C	chr2	32215682	T	C	+		SLC30A6	intron_variant	c.886-4531T>C			driver
chr2:g.33095778T>G	chr2	33095778	T	G	+		LTBP1	intron_variant	c.864-14804T>G			driver
chr2:g.34329440C>G	chr2	34329440	C	G	+		LINC01320	lnc_RNA				driver
chr2:g.38769064C>T	chr2	38769064	C	T	+		GEMIN6	intron_variant	c.-186+865C>T			passenger
chr2:g.40035110G>T	chr2	40035110	G	T	+		SLC8A1-AS1	lnc_RNA				driver
chr2:g.48681341T>C	chr2	48681341	T	C	+		STON1-GTF2A1L	intron_variant	c.3441+9661T>C			driver
chr2:g.54608766C>G	chr2	54608766	C	G	+		SPTBN1	intron_variant	c.301-3395C>G			driver
chr2:g.58575802T>C	chr2	58575802	T	C	+		LINC01122	lnc_RNA				driver
chr2:g.60897967G>C	chr2	60897967	G	C	+		REL	intron_variant	c.303-3025G>C		Oncogene	driver
chr2:g.70952878T>A	chr2	70952878	T	A	+		ATP6V1B1	intron_variant	c.175-5168T>A			driver
chr2:g.79837899T>A	chr2	79837899	T	A	+		CTNNA2	intron_variant	c.299-20114T>A		Oncogene	driver
chr2:g.100985838A>G	chr2	100985838	A	G	+		NPAS2	intron_variant	c.1630-2241A>G			passenger
chr2:g.112535569C>G	chr2	112535569	C	G	+		TTL	3_prime_UTR_variant	c.*6774C>G			driver
chr2:g.113259117T>C	chr2	113259117	T	C	+		PAX8	intron_variant	c.26-12198A>G		fusion	driver
chr2:g.114846336T>A	chr2	114846336	T	A	+		DPP10	intron_variant	c.60+403498T>A			driver
chr2:g.115109813T>A	chr2	115109813	T	A	+		DPP10	intron_variant	c.61-199426T>A			passenger
chr2:g.115607698A>T	chr2	115607698	A	T	+		DPP10	intron_variant	c.453+81726A>T			driver
chr2:g.115667259G>T	chr2	115667259	G	T	+		DPP10	intron_variant	c.454-22428G>T			driver
chr2:g.124740757C>G	chr2	124740757	C	G	+		CNTNAP5	intron_variant	c.2075-6472C>G			driver
chr2:g.124798190T>G	chr2	124798190	T	G	+	Yes	CNTNAP5	synonymous_variant	c.3084T>G	p.Ala1028=		passenger
chr2:g.132479603A>T	chr2	132479603	A	T	+		GPR39	intron_variant	c.856+61705A>T			driver
chr2:g.137322267T>A	chr2	137322267	T	A	+		THSD7B	intron_variant	c.2500+46241T>A			driver
chr2:g.148334545G>C	chr2	148334545	G	C	+		MBD5	intron_variant	c.-679-7669G>C			passenger
chr2:g.148723699A>T	chr2	148723699	A	T	+		EPC2	intron_variant	c.314-19923A>T			driver
chr2:g.149074274A>G	chr2	149074274	A	G	+		LYPD6B	intron_variant	c.-67+35473A>G			passenger
chr2:g.150275288T>A	chr2	150275288	T	A	+		LINC01818	lnc_RNA				driver

chr2:g.150567904C>T	chr2	150567904	C	T	+		LINC01920	lnc_RNA				driver
chr2:g.157936587A>G	chr2	157936587	A	G	+		UPP2	intron_variant	c.-19-58593A>G			passenger
chr2:g.168552303T>G	chr2	168552303	T	G	+		CERS6	intron_variant	c.276+4602T>G			passenger
chr2:g.190962933G>T	chr2	190962933	G	T	+	Yes	GLS	missense_variant	c.1957G>T	p.Asp653Tyr		Damaging
chr2:g.197861305G>C	chr2	197861305	G	C	+		PLCL1	intron_variant	c.240+55966G>C			driver
chr2:g.198551454C>A	chr2	198551454	C	A	+		AC019330.1	lnc_RNA				passenger
chr2:g.202638873A>T	chr2	202638873	A	T	+		FAM117B	intron_variant	c.601+3085A>T			driver
chr2:g.213671699A>T	chr2	213671699	A	T	+		SPAG16	intron_variant	c.1070+181609A>T			driver
chr2:g.213987362C>A	chr2	213987362	C	A	+		SPAG16	intron_variant	c.1401-26589C>A			driver
chr2:g.214018972A>T	chr2	214018972	A	T	+		SPAG16	intron_variant	c.1527+4895A>T			driver
chr2:g.220120078C>T	chr2	220120078	C	T	+		AC019211.1	lnc_RNA				driver
chr2:g.220508784C>T	chr2	220508784	C	T	+		AC067956.1	lnc_RNA				driver
chr2:g.226990241A>G	chr2	226990241	A	G	+		RHBDD1	intron_variant	c.857-5190A>G			passenger
chr2:g.231394417G>A	chr2	231394417	G	A	+		B3GNT7	2kb_upstream_variant	c.-1387G>A			driver
chr2:g.235953574G>A	chr2	235953574	G	A	+		AGAP1	intron_variant	c.1484-14888G>A			driver
chr2:g.236286454G>T	chr2	236286454	G	T	+		AC019068.1	lnc_RNA				driver
chr3:g.6365657A>T	chr3	6365657	A	T	+		AC026167.1	lnc_RNA				driver
chr3:g.6609337G>A	chr3	6609337	G	A	+		AC069277.1	lnc_RNA				driver
chr3:g.7305152T>G	chr3	7305152	T	G	+		GRM7	intron_variant	c.879-1346T>G			passenger
chr3:g.10839981C>T	chr3	10839981	C	T	+		SLC6A11	intron_variant	c.624-4233C>T			driver
chr3:g.14032170A>C	chr3	14032170	A	C	+							passenger
chr3:g.19948616T>G	chr3	19948616	T	G	+		RAB5A	intron_variant	c.-94+1095T>G			driver
chr3:g.34412954G>T	chr3	34412954	G	T	+		LINC01811	lnc_RNA				driver
chr3:g.44771843A>G	chr3	44771843	A	G	+		KIF15	intron_variant	c.20-2552A>G			passenger
chr3:g.49509162T>A	chr3	49509162	T	A	+		DAG1	intron_variant	c.-116-1257T>A			passenger
chr3:g.54575295G>A	chr3	54575295	G	A	+		CACNA2D3	intron_variant	c.888+5191G>A			driver
chr3:g.81088421C>A	chr3	81088421	C	A	+		LINC02027	lnc_RNA				driver
chr3:g.88444902A>G	chr3	88444902	A	G	+		CSNKA2IP	intron_variant	c.-270-20186A>G			driver
chr3:g.94956521T>C	chr3	94956521	T	C	+		LINC00879	lnc_RNA				driver
chr3:g.97834926G>A	chr3	97834926	G	A	+		CRYBG3	intron_variant	c.150-8269G>A			passenger
chr3:g.99670402C>T	chr3	99670402	C	T	+		COL8A1	intron_variant	c.-129+31738C>T			driver
chr3:g.111174448G>A	chr3	111174448	G	A	+		NECTIN3	intron_variant	c.1222-17903G>A			driver
chr3:g.111625372A>C	chr3	111625372	A	C	+		CD96	intron_variant	c.1321+968A>C			driver
chr3:g.111841793C>A	chr3	111841793	C	A	+		PLCXD2	intron_variant	c.*34-4028C>A			driver
chr3:g.116714869T>A	chr3	116714869	T	A	+		TUSC7	lnc_RNA				driver
chr3:g.124126286T>C	chr3	124126286	T	C	+		KALRN	intron_variant	c.67+31382T>C			driver
chr3:g.127955278C>T	chr3	127955278	C	T	+		KBTBD12	intron_variant	c.1493-7911C>T			driver
chr3:g.128888189C>G	chr3	128888189	C	G	+		ACAD9	intron_variant	c.244+3443C>G			driver
chr3:g.130859697C>A	chr3	130859697	C	A	+		ATP2C1	intron_variant	c.108+8769C>A			driver
chr3:g.133186058G>A	chr3	133186058	G	A	+		TMEM108	intron_variant	c.-46-43208G>A			passenger
chr3:g.134700794G>T	chr3	134700794	G	T	+		EPHB1	processed_transcript				driver
chr3:g.134753318A>C	chr3	134753318	A	C	+		EPHB1	processed_transcript				driver
chr3:g.136881088C>G	chr3	136881088	C	G	+		NCK1	intron_variant	c.-19+18735C>G			driver
chr3:g.139174032G>T	chr3	139174032	G	T	+		MRPS22	intron_variant	c.-142-6258G>T			driver
chr3:g.141521412G>A	chr3	141521412	G	A	+		RASA2	intron_variant	c.355+4981G>A			passenger
chr3:g.142703070G>C	chr3	142703070	G	C	+		PLS1	intron_variant	c.1372-798G>C			driver
chr3:g.142979146A>G	chr3	142979146	A	G	+		PAQR9-AS1	lnc_RNA				passenger
chr3:g.144047347C>G	chr3	144047347	C	G	+		DIPK2A	processed_transcript				driver
chr3:g.148282041A>G	chr3	148282041	A	G	+		LINC02046	lnc_RNA				driver
chr3:g.150430002G>T	chr3	150430002	G	T	+		TSC22D2	intron_variant	c.2030+6882G>T			driver
chr3:g.155089896G>A	chr3	155089896	G	A	+		MME	intron_variant	c.196+4802G>A			driver
chr3:g.158454605A>G	chr3	158454605	A	G	+		RSRC1	intron_variant	c.584-6330A>G			passenger
chr3:g.173997558T>A	chr3	173997558	T	A	+		NLGN1	intron_variant	c.646+189726T>A			passenger
chr3:g.174065536A>C	chr3	174065536	A	C	+		NLGN1	intron_variant	c.647-209779A>C			driver
chr3:g.174695828C>G	chr3	174695828	C	G	+		NAALADL2	processed_transcript				driver
chr3:g.174800812C>T	chr3	174800812	C	T	+							passenger
chr3:g.175149930C>T	chr3	175149930	C	T	+		NAALADL2	intron_variant	c.545+52639C>T			driver
chr3:g.177597781T>A	chr3	177597781	T	A	+		LINC00578	lnc_RNA				driver
chr3:g.189881926A>G	chr3	189881926	A	G	+		TP63	intron_variant	c.1350-4468A>G		Oncogene	NA
chr4:g.1114786C>T	chr4	1114786	C	T	+		RNF212	2kb_upstream_variant	c.-1322G>A			driver
chr4:g.1210659C>T	chr4	1210659	C	T	+		SPON2	2kb_upstream_variant	c.-2322G>A			passenger
chr4:g.3421442G>A	chr4	3421442	G	A	+		RGS12	intron_variant	c.2838+724G>A			driver
chr4:g.3432622A>T	chr4	3432622	A	T	+		RGS12	intron_variant	c.4114+1667A>T			driver
chr4:g.7006997G>T	chr4	7006997	G	T	+		TBC1D14	intron_variant	c.1446+271G>T			driver
chr4:g.13746398T>C	chr4	13746398	T	C	+		LINC01182	lnc_RNA				driver
chr4:g.13781262A>T	chr4	13781262	A	T	+		LINC01182	lnc_RNA				driver
chr4:g.19574266G>T	chr4	19574266	G	T	+		AC024230.1	lnc_RNA				driver
chr4:g.23019814T>G	chr4	23019814	T	G	+		AC097512.1	lnc_RNA				driver
chr4:g.23048783T>C	chr4	23048783	T	C	+		AC097512.1	lnc_RNA				driver
chr4:g.23670561G>C	chr4	23670561	G	C	+		AC093607.1	lnc_RNA				driver
chr4:g.29169963C>G	chr4	29169963	C	G	+		AC109349.1	lnc_RNA				driver
chr4:g.56436046C>G	chr4	56436046	C	G	+		PPAT	2kb_upstream_variant	c.-569G>C			driver

chr4:g.59028181A>T	chr4	59028181	A	T	+		LINC02429	lnc_RNA				driver
chr4:g.61981294T>C	chr4	61981294	T	C	+		ADGRL3	intron_variant	c.3015+1522T>C			passenger
chr4:g.89894038A>G	chr4	89894038	A	G	+		MMRN1	2kb_upstream_variant	c.-934A>G			driver
chr4:g.93163126G>T	chr4	93163126	G	T	+		GRID2	intron_variant	c.736-44278G>T			driver
chr4:g.93166692T>A	chr4	93166692	T	A	+		GRID2	intron_variant	c.736-40712T>A			driver
chr4:g.96505211C>A	chr4	96505211	C	A	+		LINC02267	lnc_RNA				passenger
chr4:g.98380687A>T	chr4	98380687	A	T	+		RAP1GDS1	intron_variant	c.508+1524A>T		Oncogene	NA
chr4:g.101746289A>G	chr4	101746289	A	G	+		BANK1	intron_variant	c.25+24856A>G			passenger
chr4:g.104011805T>A	chr4	104011805	T	A	+		LINC02503	lnc_RNA				driver
chr4:g.108254955G>T	chr4	108254955	G	T	+		LEF1-AS1	lnc_RNA				driver
chr4:g.113196977C>A	chr4	113196977	C	A	+		ANK2	intron_variant	c.285+511C>A			driver
chr4:g.117635919A>G	chr4	117635919	A	G	+		LINC01378	lnc_RNA				passenger
chr4:g.122158422G>T	chr4	122158422	G	T	+		KIAA1109	intron_variant	c.-34+4310G>T			driver
chr4:g.139696519G>C	chr4	139696519	G	C	+		MGST2	intron_variant	c.229+1252G>C			passenger
chr4:g.143695068A>G	chr4	143695068	A	G	+		FREM3	intron_variant	c.5185+423T>C			passenger
chr4:g.149213486C>G	chr4	149213486	C	G	+		LINC02355	lnc_RNA				driver
chr4:g.153203673C>T	chr4	153203673	C	T	+		TRIM2	2kb_upstream_variant	c.-858C>T			driver
chr4:g.153616110G>C	chr4	153616110	G	C	+		TMEM131L	intron_variant	c.3567+3710G>C			driver
chr4:g.163115073T>G	chr4	163115073	T	G	+		NAF1	intron_variant	c.115-4783A>C			driver
chr4:g.172577463A>T	chr4	172577463	A	T	+		GALNTL6	intron_variant	c.553+228774A>T			driver
chr4:g.173243720A>C	chr4	173243720	A	C	+		GALNT7	intron_variant	c.127-4260A>C			driver
chr4:g.182280202A>T	chr4	182280202	A	T	+		TENM3	intron_variant	c.-76+36726A>T			driver
chr4:g.182431205G>T	chr4	182431205	G	T	+		TENM3	intron_variant	c.511+84276G>T			driver
chr4:g.182507374C>A	chr4	182507374	C	A	+		TENM3	intron_variant	c.512-93550C>A			driver
chr4:g.182563868T>A	chr4	182563868	T	A	+		TENM3	intron_variant	c.512-37056T>A			driver
chr5:g.5185948T>A	chr5	5185948	T	A	+		ADAMTS16	intron_variant	c.764-104T>A			driver
chr5:g.7154175C>A	chr5	7154175	C	A	+		LINC02196	lnc_RNA				passenger
chr5:g.7548656T>G	chr5	7548656	T	G	+		ADCY2	intron_variant	c.570+27757T>G			driver
chr5:g.14345341G>C	chr5	14345341	G	C	+		TRIO	intron_variant	c.2046+8614G>C			driver
chr5:g.23968557G>A	chr5	23968557	G	A	+		C5orf17	lnc_RNA				driver
chr5:g.25313695A>G	chr5	25313695	A	G	+		LINC02211	lnc_RNA				passenger
chr5:g.40477185C>G	chr5	40477185	C	G	+		AC114977.2	lnc_RNA				driver
chr5:g.57437457C>A	chr5	57437457	C	A	+		AC025470.2	lnc_RNA				driver
chr5:g.64928228T>C	chr5	64928228	T	C	+		CWC27	intron_variant	c.1042+42682T>C			driver
chr5:g.64956701A>G	chr5	64956701	A	G	+		CWC27	intron_variant	c.1043-15002A>G			passenger
chr5:g.64957486G>T	chr5	64957486	G	T	+		CWC27	intron_variant	c.1043-14217G>T			driver
chr5:g.68191817G>T	chr5	68191817	G	T	+		LINC02219	lnc_RNA				driver
chr5:g.81982435G>A	chr5	81982435	G	A	+		ATG10	intron_variant	c.-12-5124G>A			driver
chr5:g.91044944G>A	chr5	91044944	G	A	+		ADGRV1	intron_variant	c.18153-27503G>A			driver
chr5:g.92436571G>A	chr5	92436571	G	A	+		AC114316.1	lnc_RNA				passenger
chr5:g.97887192T>A	chr5	97887192	T	A	+		LINC02234	lnc_RNA				driver
chr5:g.127421768T>G	chr5	127421768	T	G	+		MEGF10	intron_variant	c.1591-902T>G			passenger
chr5:g.134445724A>T	chr5	134445724	A	T	+		AC109454.3	lnc_RNA				driver
chr5:g.152260358G>T	chr5	152260358	G	T	+		LINC01933	lnc_RNA				driver
chr5:g.153700837G>T	chr5	153700837	G	T	+		GRIA1	intron_variant	c.1452+1764G>T			driver
chr5:g.159434120G>T	chr5	159434120	G	T	+		AC008691.1	lnc_RNA				driver
chr5:g.165506487T>A	chr5	165506487	T	A	+		AC008415.1	lnc_RNA				driver
chr5:g.170081575G>A	chr5	170081575	G	A	+		DOCK2	intron_variant	c.5288-267G>A			driver
chr6:g.12044114T>A	chr6	12044114	T	A	+		HIVEP1	intron_variant	c.40+28446T>A			driver
chr6:g.20883039T>G	chr6	20883039	T	G	+		CDKAL1	intron_variant	c.742+36861T>G			driver
chr6:g.36105324C>A	chr6	36105324	C	A	+		MAPK14	intron_variant	c.842-2131C>A			driver
chr6:g.37321898G>A	chr6	37321898	G	A	+		TBC1D22B	intron_variant	c.1389+4692G>A			driver
chr6:g.39946885C>T	chr6	39946885	C	T	+							driver
chr6:g.68779117T>A	chr6	68779117	T	A	+		ADGRB3	intron_variant	c.757+139685T>A			passenger
chr6:g.68868792G>T	chr6	68868792	G	T	+		ADGRB3	intron_variant	c.758-61767G>T			driver
chr6:g.69339927C>A	chr6	69339927	C	A	+		ADGRB3	intron_variant	c.3459+423C>A			driver
chr6:g.70056631C>A	chr6	70056631	C	A	+		COL19A1	intron_variant	c.1171-11792C>A			driver
chr6:g.70075619A>G	chr6	70075619	A	G	+		COL19A1	intron_variant	c.1224+7143A>G			passenger
chr6:g.70780496G>A	chr6	70780496	G	A	+		SMAP1	intron_variant	c.414+7071G>A			driver
chr6:g.73123940G>T	chr6	73123940	G	T	+		KCNQ5	intron_variant	c.1221-546G>T			driver
chr6:g.73797890A>G	chr6	73797890	A	G	+		CD109	intron_variant	c.2878+5088A>G			passenger
chr6:g.74111617A>T	chr6	74111617	A	T	+		AL357507.1	lnc_RNA				driver
chr6:g.74152850T>C	chr6	74152850	T	C	+		AL357507.1	lnc_RNA				passenger
chr6:g.101286867C>T	chr6	101286867	C	T	+		GRIK2	intron_variant	c.-294+42211C>T			driver
chr6:g.101754464A>G	chr6	101754464	A	G	+		GRIK2	intron_variant	c.952-45184A>G			passenger
chr6:g.116176772G>A	chr6	116176772	G	A	+		NT5DC1	intron_variant	c.530-44282G>A			passenger
chr6:g.116714965G>A	chr6	116714965	G	A	+		KPNA5	intron_variant	c.657-1254G>A			driver
chr6:g.118224818A>G	chr6	118224818	A	G	+		SLC35F1	intron_variant	c.350-10691A>G			passenger
chr6:g.124773947G>A	chr6	124773947	G	A	+		NKAIN2	intron_variant	c.475-17392G>A			driver
chr6:g.131733705G>A	chr6	131733705	G	A	+	Yes	ENPP3	missense_variant	c.2071G>A	p.Gly691Ser		Tolerated
chr6:g.135957066T>C	chr6	135957066	T	C	+		PDE7B	intron_variant	c.82+9542T>C			passenger
chr6:g.146604146G>A	chr6	146604146	G	A	+		ADGB	intron_variant	c.74+5032G>A			passenger
chr6:g.147794379C>T	chr6	147794379	C	T	+		AL033504.1	lnc_RNA				driver
chr6:g.154116315G>C	chr6	154116315	G	C	+		OPRM1	intron_variant	c.1444-2368G>C			driver

chr6:g.157200395G>C	chr6	157200395	G	C	+		ARID1B	intron_variant	c.4231-310G>C		TSG	passenger
chr6:g.159222508C>T	chr6	159222508	C	T	+		FNDC1	intron_variant	c.766+812C>T			driver
chr6:g.162894670G>T	chr6	162894670	G	T	+		PACRG	intron_variant	c.291+80389G>T			driver
chr6:g.162909120C>A	chr6	162909120	C	A	+		PACRG	intron_variant	c.291+94839C>A			driver
chr7:g.855276G>A	chr7	855276	G	A	+		SUN1	intron_variant	c.1350+270G>A			passenger
chr7:g.7825972T>A	chr7	7825972	T	A	+		UMAD1	intron_variant	c.156+24229T>A			driver
chr7:g.8686331C>G	chr7	8686331	C	G	+		NXPH1	intron_variant	c.55-64677C>G			driver
chr7:g.10704508C>A	chr7	10704508	C	A	+		AC004415.1	lnc_RNA				driver
chr7:g.11129473A>T	chr7	11129473	A	T	+		PHF14	intron_variant	c.2772+18006A>T			driver
chr7:g.12514849G>A	chr7	12514849	G	A	+		AC013470.2	intron_variant	c.4-8122G>A			driver
chr7:g.16243030C>A	chr7	16243030	C	A	+		CRPPA	intron_variant	c.1119+15360G>T			driver
chr7:g.18227294T>C	chr7	18227294	T	C	+		HDAC9	intron_variant	c.25+64945T>C			driver
chr7:g.20365348C>G	chr7	20365348	C	G	+		ITGB8	intron_variant	c.213+1626C>G			driver
chr7:g.24617711G>C	chr7	24617711	G	C	+		MPP6	intron_variant	c.-2-5955G>C			driver
chr7:g.30976222C>T	chr7	30976222	C	T	+		GHRHR	intron_variant	c.975-207C>T			driver
chr7:g.34692963T>A	chr7	34692963	T	A	+		NPSR1	intron_variant	c.280+8279T>A			driver
chr7:g.36267210C>T	chr7	36267210	C	T	+		EEPD1	intron_variant	c.931-13905C>T			driver
chr7:g.39836131A>T	chr7	39836131	A	T	+							driver
chr7:g.49795584C>A	chr7	49795584	C	A	+		VWC2	intron_variant	c.697-7127C>A			driver
chr7:g.51551203G>T	chr7	51551203	G	T	+		AC005999.2	lnc_RNA				driver
chr7:g.55050817A>T	chr7	55050817	A	T	+		EGFR	intron_variant	c.88+31452A>T		Oncogene	driver
chr7:g.79785221G>A	chr7	79785221	G	A	+		GNAI1	intron_variant	c.-434+12221G>A			passenger
chr7:g.79888512T>G	chr7	79888512	T	G	+		GNAI1	intron_variant	c.-433-68458T>G			driver
chr7:g.84548649A>T	chr7	84548649	A	T	+		AC003984.1	lnc_RNA				driver
chr7:g.85467638T>G	chr7	85467638	T	G	+		LINC00972	lnc_RNA				driver
chr7:g.87763109C>T	chr7	87763109	C	T	+		RUNDC3B	intron_variant	c.681-7472C>T			driver
chr7:g.87768664G>A	chr7	87768664	G	A	+		RUNDC3B	intron_variant	c.681-1917G>A			driver
chr7:g.88034598T>C	chr7	88034598	T	C	+		ADAM22	intron_variant	c.324-41028T>C			passenger
chr7:g.88064841C>T	chr7	88064841	C	T	+		ADAM22	intron_variant	c.324-10785C>T			passenger
chr7:g.88865329C>G	chr7	88865329	C	G	+		ZNF804B	intron_variant	c.108+105245C>G			driver
chr7:g.88907133T>A	chr7	88907133	T	A	+		ZNF804B	intron_variant	c.108+147049T>A			driver
chr7:g.89032430T>C	chr7	89032430	T	C	+		ZNF804B	intron_variant	c.109-185725T>C			passenger
chr7:g.89319597A>T	chr7	89319597	A	T	+		ZNF804B	intron_variant	c.250-7747A>T			driver
chr7:g.90595208C>T	chr7	90595208	C	T	+		CDK14	2kb_upstream_variant	c.-1420C>T			driver
chr7:g.90617082C>T	chr7	90617082	C	T	+		CDK14	intron_variant	c.123+12833C>T			driver
chr7:g.93628884T>A	chr7	93628884	T	A	+		GNGT1	intron_variant	c.-56+37113T>A			driver
chr7:g.93690647G>A	chr7	93690647	G	A	+		GNGT1	intron_variant	c.-56+98876G>A			driver
chr7:g.93801545G>T	chr7	93801545	G	T	+		GNGT1	intron_variant	c.-55-84961G>T			driver
chr7:g.102161506A>T	chr7	102161506	A	T	+		CUX1	intron_variant	c.723+2898A>T		Oncogene	NA
chr7:g.108611327C>G	chr7	108611327	C	G	+		AC005487.1	lnc_RNA				driver
chr7:g.111123438A>G	chr7	111123438	A	G	+	Yes	LRRN3	missense_variant	c.666A>G	p.Ile222Met		0.16862
chr7:g.114388206A>T	chr7	114388206	A	T	+		FOXP2	intron_variant	c.-10-38296A>T			driver
chr7:g.120370053C>G	chr7	120370053	C	G	+		KCND2	intron_variant	c.1115+94306C>G			driver
chr7:g.120592408C>T	chr7	120592408	C	T	+		KCND2	intron_variant	c.1116-140495C>T			driver
chr7:g.121276952A>G	chr7	121276952	A	G	+		CPED1	intron_variant	c.2868+5522A>G			driver
chr7:g.121972028C>A	chr7	121972028	C	A	+		PTPRZ1	intron_variant	c.305-513C>A			driver
chr7:g.127727139G>A	chr7	127727139	G	A	+		SND1	intron_variant	c.1152+5739G>A		Oncogene	NA
chr7:g.132355690C>T	chr7	132355690	C	T	+		PLXNA4	intron_variant	c.1372-57468G>A			driver
chr7:g.146394260G>A	chr7	146394260	G	A	+		CNTNAP2	intron_variant	c.97+277287G>A		TSG	driver
chr7:g.146402440G>C	chr7	146402440	G	C	+		CNTNAP2	intron_variant	c.97+285467G>C		TSG	driver
chr7:g.147397052G>A	chr7	147397052	G	A	+		CNTNAP2	intron_variant	c.1670+1272G>A		TSG	passenger
chr7:g.147459403G>T	chr7	147459403	G	T	+		CNTNAP2	intron_variant	c.1671-26532G>T		TSG	driver
chr7:g.147899791A>C	chr7	147899791	A	C	+		CNTNAP2	intron_variant	c.2099-3774A>C		TSG	passenger
chr7:g.151238685G>A	chr7	151238685	G	A	+		CHPF2	3_prime_UTR_variant	c.*4G>A			driver
chr7:g.157170398T>A	chr7	157170398	T	A	+	Yes	UBE3C	stop_gained	c.290T>A	p.Leu97Ter		driver
chr8:g.1219074A>T	chr8	1219074	A	T	+		DLGAP2	intron_variant	c.74-39777A>T			driver
chr8:g.10692778G>A	chr8	10692778	G	A	+		C8orf74	intron_variant	c.242-4821G>A			passenger
chr8:g.13704710G>T	chr8	13704710	G	T	+							driver
chr8:g.28641389G>T	chr8	28641389	G	T	+		EXTL3	intron_variant	c.-53+18579G>T			driver
chr8:g.28938416C>T	chr8	28938416	C	T	+		HMBOX1	intron_variant	c.-57-25395C>T			driver
chr8:g.32274889G>T	chr8	32274889	G	T	+		NRG1	intron_variant	c.38-320939G>T		TSG	NA
chr8:g.32399184C>G	chr8	32399184	C	G	+		NRG1	intron_variant	c.38-196644C>G		TSG	NA
chr8:g.33808553G>A	chr8	33808553	G	A	+		AF279873.3	lnc_RNA				driver
chr8:g.38252938C>T	chr8	38252938	C	T	+		DDHD2	intron_variant	c.1721-19C>T			driver
chr8:g.39656200A>T	chr8	39656200	A	T	+		ADAM18	intron_variant	c.1231-7595A>T			driver
chr8:g.40386850G>A	chr8	40386850	G	A	+		AC105999.1	lnc_RNA				passenger
chr8:g.49980167C>G	chr8	49980167	C	G	+		SNTG1	intron_variant	c.-103+67936C>G			driver
chr8:g.50047440G>A	chr8	50047440	G	A	+		SNTG1	intron_variant	c.-102-125121G>A			passenger
chr8:g.50118399T>G	chr8	50118399	T	G	+		SNTG1	intron_variant	c.-102-54162T>G			passenger
chr8:g.55135544G>A	chr8	55135544	G	A	+		XKR4	intron_variant	c.806+32250G>A			driver
chr8:g.55512878G>A	chr8	55512878	G	A	+		XKR4	intron_variant	c.1007-10403G>A			driver
chr8:g.62890803G>A	chr8	62890803	G	A	+		NKAIN3	intron_variant	c.472-27650G>A			driver
chr8:g.66796513G>A	chr8	66796513	G	A	+		SGK3	intron_variant	c.97-2029G>A			passenger
chr8:g.68026250G>A	chr8	68026250	G	A	+		PREX2	intron_variant	c.442-972G>A		Oncogene	passenger
chr8:g.68331716T>C	chr8	68331716	T	C	+		C8orf34	intron_variant	c.327+377T>C			driver
chr8:g.68339273A>C	chr8	68339273	A	C	+		C8orf34	intron_variant	c.327+7934A>C			passenger
chr8:g.68500226G>T	chr8	68500226	G	T	+		C8orf34	intron_variant	c.765+12175G>T			driver
chr8:g.69589176C>T	chr8	69589176	C	T	+		SULF1	intron_variant	c.734+35C>T			driver

chr8:g.70610436A>G	chr8	70610436	A	G	+		LACTB2-AS1	lnc_RNA				passenger
chr8:g.76780312A>T	chr8	76780312	A	T	+		ZFHX4	intron_variant	c.3325+1873A>T			driver
chr8:g.91255129T>C	chr8	91255129	T	C	+		SLC26A7	intron_variant	c.193+5285T>C			passenger
chr8:g.94846060G>T	chr8	94846060	G	T	+		INTS8	intron_variant	c.1261-3402G>T			driver
chr8:g.104726002G>T	chr8	104726002	G	T	+		ZFPM2	processed_transcript				driver
chr8:g.104809718A>C	chr8	104809718	A	C	+		ZFPM2	processed_transcript				driver
chr8:g.105004657T>A	chr8	105004657	T	A	+		ZFPM2	processed_transcript				driver
chr8:g.105475871G>T	chr8	105475871	G	T	+		ZFPM2	intron_variant	c.301+31490G>T			driver
chr8:g.111415309A>T	chr8	111415309	A	T	+		LINC02237	lnc_RNA				driver
chr8:g.117137481C>G	chr8	117137481	C	G	+		SLC30A8	intron_variant	c.71+2083C>G			driver
chr8:g.124871585G>A	chr8	124871585	G	A	+		LINC00964	lnc_RNA				driver
chr8:g.136087370T>C	chr8	136087370	T	C	+		LINC02055	lnc_RNA				passenger
chr8:g.136415183T>G	chr8	136415183	T	G	+		LINC02055	lnc_RNA				driver
chr8:g.136989295G>A	chr8	136989295	G	A	+		LINC02055	lnc_RNA				driver
chr8:g.137045061T>G	chr8	137045061	T	G	+		LINC02055	lnc_RNA				passenger
chr8:g.141164555C>T	chr8	141164555	C	T	+		DENND3	intron_variant	c.1450-631C>T			driver
chr8:g.144210767T>G	chr8	144210767	T	G	+		MROH1	intron_variant	c.1142-9833T>G			passenger
chr8:g.144219642G>T	chr8	144219642	G	T	+		MROH1	intron_variant	c.1142-958G>T			driver
chr9:g.11785284C>G	chr9	11785284	C	G	+		AL353595.1	lnc_RNA				driver
chr9:g.18152549G>T	chr9	18152549	G	T	+							driver
chr9:g.18503491C>T	chr9	18503491	C	T	+		ADAMTSL1	intron_variant	c.64-1338C>T			passenger
chr9:g.21936369T>C	chr9	21936369	T	C	+		MTAP	3_prime_UTR_variant	c.*5170T>C			passenger
chr9:g.72745159C>G	chr9	72745159	C	G	+		TMC1	intron_variant	c.535+2634C>G			driver
chr9:g.74623025A>G	chr9	74623025	A	G	+		RORB	intron_variant	c.8-7257A>G			passenger
chr9:g.75983637A>T	chr9	75983637	A	T	+		PCSK5	intron_variant	c.298-2495A>T			driver
chr9:g.76547409A>C	chr9	76547409	A	C	+		GCNT1	processed_transcript				driver
chr9:g.79631598C>G	chr9	79631598	C	G	+		TLE4	intron_variant	c.390+4150C>G			driver
chr9:g.81748680C>T	chr9	81748680	C	T	+		AL591368.1	lnc_RNA				passenger
chr9:g.82399061A>C	chr9	82399061	A	C	+							driver
chr9:g.92109831G>A	chr9	92109831	G	A	+		SPTLC1	intron_variant	c.166-997C>T			passenger
chr9:g.101475074T>A	chr9	101475074	T	A	+		PGAP4	3_prime_UTR_variant	c.*807A>T			driver
chr9:g.110149588A>G	chr9	110149588	A	G	+		PALM2AKAP2	intron_variant	c.2996-6731A>G			passenger
chr9:g.117707111T>A	chr9	117707111	T	A	+		TLR4	intron_variant	c.94-1452T>A			driver
chr10:g.1000042C>T	chr10	1000042	C	T	+		GTPBP4	intron_variant	c.655-635C>T			driver
chr10:g.3919585A>T	chr10	3919585	A	T	+		LINC02660	lnc_RNA				driver
chr10:g.6201498C>T	chr10	6201498	C	T	+		PFKFB3	2kb_upstream_variant	c.-1763C>T			driver
chr10:g.11079872C>G	chr10	11079872	C	G	+		CELF2	intron_variant	c.74+61709C>G			driver
chr10:g.18397432T>A	chr10	18397432	T	A	+		CACNB2	intron_variant	c.214-4492T>A			driver
chr10:g.24436887G>A	chr10	24436887	G	A	+		KIAA1217	intron_variant	c.753-1499G>A			driver
chr10:g.25482389G>A	chr10	25482389	G	A	+		GPR158	intron_variant	c.1404+15670G>A			driver
chr10:g.32878477T>C	chr10	32878477	T	C	+		CCDC7	processed_transcript				driver
chr10:g.35459032G>A	chr10	35459032	G	A	+		CCNY	intron_variant	c.155-24372G>A			driver
chr10:g.48705322G>A	chr10	48705322	G	A	+		WDFY4	intron_variant	c.-17-4394G>A			driver
chr10:g.51548650T>A	chr10	51548650	T	A	+		PRKG1	intron_variant	c.592+80814T>A			driver
chr10:g.52013842C>A	chr10	52013842	C	A	+		PRKG1	intron_variant	c.763-40642C>A			driver
chr10:g.52277477A>C	chr10	52277477	A	C	+		PRKG1	intron_variant	c.1404-3312A>C			driver
chr10:g.63710333C>T	chr10	63710333	C	T	+		AC013287.1	lnc_RNA				driver
chr10:g.68115512T>C	chr10	68115512	T	C	+		MYPN	intron_variant	c.-2+5789T>C			driver
chr10:g.68133519G>T	chr10	68133519	G	T	+		MYPN	intron_variant	c.903-9421G>T			driver
chr10:g.71054646C>A	chr10	71054646	C	A	+		AC073176.2	lnc_RNA				driver
chr10:g.71302177C>T	chr10	71302177	C	T	+		UNC5B	3_prime_UTR_variant	c.*2900C>T			passenger
chr10:g.76509385A>G	chr10	76509385	A	G	+		LRMDA	intron_variant	c.602-47824A>G			passenger
chr10:g.79110453G>A	chr10	79110453	G	A	+		ZMIZ1	intron_variant	c.-336-8462G>A			driver
chr10:g.80581459A>C	chr10	80581459	A	C	+		SH2D4B	intron_variant	c.496-7171A>C			driver
chr10:g.104764169C>A	chr10	104764169	C	A	+		SORCS3	intron_variant	c.628-78623C>A			driver
chr10:g.110876844G>T	chr10	110876844	G	T	+		PDCD4	intron_variant	c.43+774G>T			driver
chr10:g.115939846C>A	chr10	115939846	C	A	+		ATRNL1	intron_variant	c.4019-4812C>A			driver
chr10:g.130227141C>A	chr10	130227141	C	A	+		LINC02646	lnc_RNA				driver
chr10:g.130302916G>T	chr10	130302916	G	T	+		LINC02646	lnc_RNA				passenger
chr10:g.130443893C>G	chr10	130443893	C	G	+		LINC02646	lnc_RNA				driver
chr11:g.609983G>A	chr11	609983	G	A	+		PHRF1	intron_variant	c.4265-213G>A			passenger
chr11:g.3974408G>T	chr11	3974408	G	T	+		STIM1	intron_variant	c.270+6726G>T			driver
chr11:g.4823143G>T	chr11	4823143	G	T	+		MMP26	intron_variant	c.-145+55802G>T			driver
chr11:g.7253123C>T	chr11	7253123	C	T	+		SYT9	intron_variant	c.145+792C>T			driver
chr11:g.7290111G>A	chr11	7290111	G	A	+		SYT9	intron_variant	c.146-12928G>A			driver
chr11:g.7985546G>C	chr11	7985546	G	C	+		EIF3F	2kb_upstream_variant	c.-1807G>C			driver
chr11:g.12762381G>T	chr11	12762381	G	T	+		TEAD1	intron_variant	c.-54-1798G>T			passenger
chr11:g.21358003C>G	chr11	21358003	C	G	+		NELL1	intron_variant	c.1550-12850C>G			driver
chr11:g.21955521A>T	chr11	21955521	A	T	+		ANO5	processed_transcript				driver
chr11:g.21975909C>T	chr11	21975909	C	T	+		ANO5	processed_transcript				driver
chr11:g.27535728C>A	chr11	27535728	C	A	+		BDNF-AS	lnc_RNA				driver
chr11:g.28210687A>T	chr11	28210687	A	T	+		METTL15	intron_variant	c.271-375A>T			driver
chr11:g.30596321A>C	chr11	30596321	A	C	+		MPPED2-AS1	lnc_RNA				driver
chr11:g.36393809C>A	chr11	36393809	C	A	+		PRR5L	intron_variant	c.-125-7188C>A			driver
chr11:g.43449759C>G	chr11	43449759	C	G	+		TTC17	intron_variant	c.2787-323C>G			passenger
chr11:g.44903620G>T	chr11	44903620	G	T	+		TSPAN18	intron_variant	c.-10-2787G>T			driver
chr11:g.57782705A>G	chr11	57782705	A	G	+		CTNND1	intron_variant	c.-213-6332A>G			driver

chr11:g.90358347A>T	chr11	90358347	A	T	+		DISC1FP1	lnc_RNA				driver
chr11:g.92827494A>G	chr11	92827494	A	G	+		FAT3	intron_variant	c.9482-4128A>G			passenger
chr11:g.92865428C>T	chr11	92865428	C	T	+		FAT3	intron_variant	c.11659-1313C>T			driver
chr11:g.100149607T>A	chr11	100149607	T	A	+		CNTN5	intron_variant	c.1581-41519T>A			passenger
chr11:g.102783769C>T	chr11	102783769	C	T	+							driver
chr11:g.105817906T>C	chr11	105817906	T	C	+		GRIA4	intron_variant	c.488-44118T>C			passenger
chr11:g.106254497A>G	chr11	106254497	A	G	+		AP002001.3	lnc_RNA				driver
chr11:g.112576520C>T	chr11	112576520	C	T	+		LINC02763	lnc_RNA				passenger
chr11:g.112995525T>C	chr11	112995525	T	C	+		NCAM1	intron_variant	c.49-8321T>C			driver
chr11:g.120237700G>T	chr11	120237700	G	T	+		POU2F3	intron_variant	c.34+993G>T			driver
chr11:g.123902842C>A	chr11	123902842	C	A	+		OR8D4	intron_variant	c.-16+585C>A			driver
chr11:g.124803495T>G	chr11	124803495	T	G	+		AP000866.1	lnc_RNA				driver
chr11:g.128810910G>A	chr11	128810910	G	A	+	Yes	FLI1	synonymous_variant	c.1281G>A	p.Thr427=	Oncogene	NA
chr12:g.5016481G>T	chr12	5016481	G	T	+		AC005906.2	lnc_RNA				driver
chr12:g.11220171G>C	chr12	11220171	G	C	+		AC244131.2	lnc_RNA				driver
chr12:g.11579153T>C	chr12	11579153	T	C	+		LINC01252	lnc_RNA				driver
chr12:g.12133183A>G	chr12	12133183	A	G	+		LRP6	intron_variant	c.3734-1126T>C			driver
chr12:g.12141429T>C	chr12	12141429	T	C	+		LRP6	intron_variant	c.3398-2895A>G			passenger
chr12:g.12910491C>T	chr12	12910491	C	T	+		GPRC5A	intron_variant	c.922+1320C>T			driver
chr12:g.16368985A>G	chr12	16368985	A	G	+		MGST1	intron_variant	c.222-7137A>G			passenger
chr12:g.16694332G>T	chr12	16694332	G	T	+		AC007529.2	lnc_RNA				driver
chr12:g.18267544C>A	chr12	18267544	C	A	+		PIK3C2G	intron_variant	c.-79+5967C>A			driver
chr12:g.82826385G>T	chr12	82826385	G	T	+		TMTC2	intron_variant	c.84-30625G>T			driver
chr12:g.83055905C>A	chr12	83055905	C	A	+		TMTC2	intron_variant	c.2267+4887C>A			driver
chr12:g.85235966G>T	chr12	85235966	G	T	+		LRRIQ1	intron_variant	c.5016+3210G>T			driver
chr12:g.93004089G>A	chr12	93004089	G	A	+		LINC02413	lnc_RNA				driver
chr12:g.93686018G>C	chr12	93686018	G	C	+		CRADD	intron_variant	c.298+6946G>C			passenger
chr12:g.93709958T>A	chr12	93709958	T	A	+		CRADD	intron_variant	c.298+30886T>A			driver
chr12:g.101970395C>G	chr12	101970395	C	G	+							driver
chr12:g.106413827C>G	chr12	106413827	C	G	+		POLR3B	intron_variant	c.1101+2867C>G			driver
chr12:g.112921186G>A	chr12	112921186	G	A	+		OAS1	2kb_downstream_variant	c.*1633G>A			driver
chr12:g.114096415G>T	chr12	114096415	G	T	+		AC010183.2	lnc_RNA				driver
chr12:g.125300923T>A	chr12	125300923	T	A	+		TMEM132B	processed_transcript				driver
chr12:g.125315703A>T	chr12	125315703	A	T	+		TMEM132B	processed_transcript				driver
chr12:g.126141556A>G	chr12	126141556	A	G	+		LINC02359	lnc_RNA				driver
chr12:g.133174148C>T	chr12	133174148	C	T	+		AC026786.1	intron_variant	c.444-7798C>T			passenger
chr13:g.42207936C>G	chr13	42207936	C	G	+		DGKH	intron_variant	c.2602-1023C>G			driver
chr13:g.45252592C>G	chr13	45252592	C	G	+		GTF2F2	intron_variant	c.387-279C>G			driver
chr13:g.53358493T>G	chr13	53358493	T	G	+		AL450423.1	lnc_RNA				driver
chr13:g.74255601A>T	chr13	74255601	A	T	+		LINC00402	lnc_RNA				driver
chr13:g.92288921C>T	chr13	92288921	C	T	+		GPC5	intron_variant	c.1561+143932C>T		TSG	driver
chr13:g.93362767T>C	chr13	93362767	T	C	+		GPC6	intron_variant	c.160+135151T>C			driver
chr13:g.93795431G>T	chr13	93795431	G	T	+		GPC6	intron_variant	c.320-34723G>T			driver
chr13:g.93960653C>G	chr13	93960653	C	G	+		GPC6	intron_variant	c.712-67076C>G			driver
chr13:g.96713374C>T	chr13	96713374	C	T	+		HS6ST3	intron_variant	c.708-119116C>T			driver
chr13:g.101019011C>A	chr13	101019011	C	A	+		NALCN-AS1	lnc_RNA				driver
chr13:g.105752123C>T	chr13	105752123	C	T	+		LINC00343	lnc_RNA				driver
chr13:g.109180698T>G	chr13	109180698	T	G	+		MYO16	intron_variant	c.5415+1065T>G			driver
chr14:g.22557134C>T	chr14	22557134	C	T	+		AC243965.1	lnc_RNA				driver
chr14:g.32690286C>T	chr14	32690286	C	T	+		AKAP6	intron_variant	c.2880-5704C>T			driver
chr14:g.35886282A>G	chr14	35886282	A	G	+							driver
chr14:g.52818180T>C	chr14	52818180	T	C	+		AL139317.1	lnc_RNA				passenger
chr14:g.53696637T>C	chr14	53696637	T	C	+		LINC02331	lnc_RNA				driver
chr14:g.53795831C>T	chr14	53795831	C	T	+		LINC02331	lnc_RNA				driver
chr14:g.54470799C>A	chr14	54470799	C	A	+		AL359792.1	lnc_RNA				driver
chr14:g.57070176G>A	chr14	57070176	G	A	+		AL391152.1	lnc_RNA				driver
chr14:g.58790112A>T	chr14	58790112	A	T	+		LINC01500	lnc_RNA				driver
chr14:g.67895229C>T	chr14	67895229	C	T	+		RAD51B	intron_variant	c.756+8025C>T		TSG	NA
chr14:g.68690970A>C	chr14	68690970	A	C	+		RAD51B	intron_variant	c.*12-38838A>C		TSG	NA
chr14:g.74100232T>G	chr14	74100232	T	G	+		LIN52	intron_variant	c.212-923T>G			driver
chr14:g.76154177A>G	chr14	76154177	A	G	+		GPATCH2L	intron_variant	c.-10-177A>G			driver
chr14:g.78690756A>T	chr14	78690756	A	T	+		NRXN3	intron_variant	c.1216-18461A>T			driver
chr14:g.78743489G>T	chr14	78743489	G	T	+		NRXN3	intron_variant	c.2038+28350G>T			driver
chr14:g.78847139A>G	chr14	78847139	A	G	+		NRXN3	intron_variant	c.2242+43316A>G			driver
chr14:g.79288729G>C	chr14	79288729	G	C	+		NRXN3	intron_variant	c.3230-178492G>C			driver
chr14:g.79343061A>T	chr14	79343061	A	T	+		NRXN3	intron_variant	c.3230-124160A>T			driver
chr14:g.79434432C>A	chr14	79434432	C	A	+		NRXN3	intron_variant	c.3230-32789C>A			driver
chr14:g.79528812G>T	chr14	79528812	G	T	+		NRXN3	intron_variant	c.3411+61410G>T			driver
chr14:g.86351943T>C	chr14	86351943	T	C	+		LINC02309	lnc_RNA				passenger
chr14:g.88178632C>T	chr14	88178632	C	T	+		KCNK10	2kb_downstream_variant	c.*6903G>A			passenger
chr14:g.97196646A>G	chr14	97196646	A	G	+		AL158800.1	lnc_RNA				passenger
chr15:g.25995747G>T	chr15	25995747	G	T	+		LINC02346	lnc_RNA				driver
chr15:g.35686572T>C	chr15	35686572	T	C	+		DPH6-DT	lnc_RNA				driver
chr15:g.42681995G>C	chr15	42681995	G	C	+		STARD9	intron_variant	c.2066-109G>C			driver
chr15:g.45774592T>A	chr15	45774592	T	A	+		AC068722.1	lnc_RNA				driver
chr15:g.54141448T>G	chr15	54141448	T	G	+		UNC13C	intron_variant	c.2984-1570T>G			passenger
chr15:g.64896421G>A	chr15	64896421	G	A	+							driver
chr15:g.66645408G>T	chr15	66645408	G	T	+		LINC01169	lnc_RNA				driver

chr15:g.67369679G>A	chr15	67369679	G	A	+		IQCH	intron_variant	c.754-2432G>A			driver
chr15:g.71541568C>G	chr15	71541568	C	G	+		THSD4	intron_variant	c.1153-118962C>G			driver
chr15:g.81354334A>T	chr15	81354334	A	T	+		TMC3	intron_variant	c.935+1391T>A			driver
chr15:g.86518380C>G	chr15	86518380	C	G	+		AGBL1	intron_variant	c.2556-4430C>G			driver
chr15:g.89387442G>T	chr15	89387442	G	T	+		MIR9-3HG	lnc_RNA				driver
chr15:g.98766893A>C	chr15	98766893	A	C	+		IGF1R	intron_variant	c.640+58786A>C			driver
chr16:g.5359310C>T	chr16	5359310	C	T	+		RBFOX1	intron_variant	c.220-107906C>T			driver
chr16:g.5379185A>G	chr16	5379185	A	G	+		RBFOX1	intron_variant	c.220-88031A>G			passenger
chr16:g.6567608G>T	chr16	6567608	G	T	+		RBFOX1	intron_variant	c.-63-86995G>T			driver
chr16:g.6764128T>C	chr16	6764128	T	C	+		RBFOX1	intron_variant	c.-16+109478T>C			passenger
chr16:g.9472022A>G	chr16	9472022	A	G	+		AC012178.1	lnc_RNA				passenger
chr16:g.9614789C>T	chr16	9614789	C	T	+		AC007221.1	lnc_RNA				passenger
chr16:g.16046339T>C	chr16	16046339	T	C	+		ABCC1	intron_variant	c.1218+326T>C			passenger
chr16:g.31180128G>A	chr16	31180128	G	A	+		FUS	5_prime_UTR_variant	c.-87G>A		TSG	NA
chr16:g.48586686C>A	chr16	48586686	C	A	+		N4BP1	intron_variant	c.198+23089G>T			driver
chr16:g.51629907T>C	chr16	51629907	T	C	+		HNRNPA1P48	intron_variant	c.-622-15424T>C			driver
chr16:g.58197553G>A	chr16	58197553	G	A	+		CSNK2A2	intron_variant	c.104+80C>T			driver
chr16:g.64500805T>C	chr16	64500805	T	C	+		AC092131.1	lnc_RNA				driver
chr16:g.65395762G>T	chr16	65395762	G	T	+		AC009055.1	lnc_RNA				driver
chr16:g.68856744T>G	chr16	68856744	T	G	+		TANGO6	intron_variant	c.95-3140T>G			driver
chr16:g.68910195A>G	chr16	68910195	A	G	+		TANGO6	intron_variant	c.1992+793A>G			driver
chr16:g.76798398T>G	chr16	76798398	T	G	+		AC106729.1	lnc_RNA				passenger
chr16:g.82855986T>C	chr16	82855986	T	C	+		CDH13	intron_variant	c.46-2376T>C			driver
chr16:g.83258659C>G	chr16	83258659	C	G	+		CDH13	intron_variant	c.636+41162C>G			driver
chr16:g.85428641G>T	chr16	85428641	G	T	+							passenger
chr16:g.89686836C>T	chr16	89686836	C	T	+		CDK10	intron_variant	c.87+39C>T			driver
chr17:g.11335618G>T	chr17	11335618	G	T	+		SHISA6	intron_variant	c.800-43796G>T			driver
chr17:g.11377509G>A	chr17	11377509	G	A	+		SHISA6	intron_variant	c.800-1905G>A			driver
chr17:g.11719748C>G	chr17	11719748	C	G	+		DNAH9	intron_variant	c.5709+258C>G			driver
chr17:g.17752827C>A	chr17	17752827	C	A	+		RAI1	intron_variant	c.-17+28668C>A			driver
chr17:g.17789733G>A	chr17	17789733	G	A	+		RAI1	intron_variant	c.-16-3200G>A			driver
chr17:g.31476832C>T	chr17	31476832	C	T	+		RAB11FIP4	intron_variant	c.337-40819C>T			driver
chr17:g.59808050A>G	chr17	59808050	A	G	+		VMP1	intron_variant	c.715-746A>G			driver
chr17:g.68626044T>C	chr17	68626044	T	C	+		LINC01482	lnc_RNA				passenger
chr17:g.70173268T>G	chr17	70173268	T	G	+		KCNJ2	intron_variant	c.-216-1556T>G			driver
chr17:g.75105256G>A	chr17	75105256	G	A	+		SLC16A5	intron_variant	c.1365-624G>A			driver
chr17:g.75521466G>A	chr17	75521466	G	A	+	Yes	TSEN54	synonymous_variant	c.579G>A	p.Leu193=		passenger
chr18:g.1683453C>T	chr18	1683453	C	T	+		AC019183.1	lnc_RNA				driver
chr18:g.7784671A>C	chr18	7784671	A	C	+		PTPRM	intron_variant	c.196+10400A>C			driver
chr18:g.9813977G>T	chr18	9813977	G	T	+		RAB31	intron_variant	c.202-43G>T			driver
chr18:g.28724378G>T	chr18	28724378	G	T	+		AC090365.1	lnc_RNA				passenger
chr18:g.44878670G>A	chr18	44878670	G	A	+		SETBP1	intron_variant	c.540+9387G>A		Oncogene	NA
chr18:g.45403970G>A	chr18	45403970	G	A	+		SLC14A2	intron_variant	c.-124-79263G>A			driver
chr18:g.50891442T>G	chr18	50891442	T	G	+		ME2	intron_variant	c.-12-4367T>G			driver
chr18:g.53314878G>T	chr18	53314878	G	T	+		DCC	intron_variant	c.2054-7169G>T			driver
chr18:g.55777288T>C	chr18	55777288	T	C	+		AC022031.2	lnc_RNA				passenger
chr18:g.59063290G>A	chr18	59063290	G	A	+							driver
chr18:g.63674039C>G	chr18	63674039	C	G	+							driver
chr18:g.63921453T>A	chr18	63921453	T	A	+		SERPINB10	intron_variant	c.490+1548T>A			driver
chr18:g.67690426A>T	chr18	67690426	A	T	+		AC114689.3	lnc_RNA				driver
chr19:g.1194473G>T	chr19	1194473	G	T	+							passenger
chr19:g.5111065G>T	chr19	5111065	G	T	+		KDM4B	intron_variant	c.1115+247G>T			driver
chr19:g.6813315G>C	chr19	6813315	G	C	+		VAV1	intron_variant	c.205-7387G>C		fusion	driver
chr19:g.29672573C>G	chr19	29672573	C	G	+		PLEKHF1	intron_variant	c.-16-1251C>G			driver
chr19:g.30302031G>T	chr19	30302031	G	T	+		ZNF536	processed_transcript				driver
chr19:g.30858645C>A	chr19	30858645	C	A	+		AC011507.1	lnc_RNA				driver
chr19:g.37018963T>A	chr19	37018963	T	A	+							driver
chr19:g.38040133G>C	chr19	38040133	G	C	+		SIPA1L3	intron_variant	c.-311+10977G>C			driver
chr19:g.39446117A>G	chr19	39446117	A	G	+		SUPT5H	intron_variant	c.75+152A>G			driver
chr19:g.41258349G>A	chr19	41258349	G	A	+		AXL	intron_variant	c.2333+720G>A			driver
chr19:g.48756848T>A	chr19	48756848	T	A	+		FGF21	intron_variant	c.236-78T>A			passenger
chr19:g.49007141G>A	chr19	49007141	G	A	+	Yes	RUVBL2	missense_variant	c.389G>A	p.Arg130His		0.27859
chr19:g.54453223A>C	chr19	54453223	A	C	+		LENG8	intron_variant	c.316-323A>C			passenger
chr19:g.56739228A>G	chr19	56739228	A	G	+		AC006115.2	lnc_RNA				driver
chr19:g.57495456G>A	chr19	57495456	G	A	+		ZNF419	2kb_downstream_variant	c.*1366G>A			passenger
chr19:g.58253805T>A	chr19	58253805	T	A	+		ZNF544	intron_variant	c.244+7011T>A			driver
chr20:g.2153870A>G	chr20	2153870	A	G	+							driver
chr20:g.2557033A>T	chr20	2557033	A	T	+		TMC2	intron_variant	c.83-1423A>T			driver
chr20:g.10942406T>A	chr20	10942406	T	A	+		AL050403.2	lnc_RNA				driver
chr20:g.15633298G>T	chr20	15633298	G	T	+		MACROD2	intron_variant	c.645+133451G>T			driver
chr20:g.21172515C>G	chr20	21172515	C	G	+		KIZ	intron_variant	c.1352+9356C>G			driver
chr20:g.37723808T>G	chr20	37723808	T	G	+		CTNNBL1	intron_variant	c.31-9071T>G			driver
chr20:g.37909558C>A	chr20	37909558	C	A	+		VSTM2L	intron_variant	c.121+6087C>A			driver
chr20:g.45809636C>T	chr20	45809636	C	T	+		DNTTIP1	intron_variant	c.795+451C>T			driver
chr20:g.49289302A>G	chr20	49289302	A	G	+		ZFAS1	lnc_RNA				passenger
chr20:g.53111989T>G	chr20	53111989	T	G	+		TSHZ2	intron_variant	c.40+138656T>G			passenger
chr20:g.53345485G>A	chr20	53345485	G	A	+		TSHZ2	intron_variant	c.*8+88914G>A			driver
chr20:g.58593206C>A	chr20	58593206	C	A	+		APCDD1L-DT	lnc_RNA				driver
chr20:g.59745604G>A	chr20	59745604	G	A	+		PHACTR3	intron_variant	c.281-2154G>A			passenger
chr21:g.14216250C>A	chr21	14216250	C	A	+	Yes	RBM11	synonymous_variant	c.64C>A	p.Arg22=		passenger
chr21:g.22337827G>T	chr21	22337827	G	T	+		AP000561.1	lnc_RNA				driver
chr21:g.34747490T>G	chr21	34747490	T	G	+		LINC01426	lnc_RNA				passenger
chr22:g.39472916G>T	chr22	39472916	G	T	+		MGAT3	intron_variant	c.-1-14431G>T			driver

chr22:g.42786007C>T	chr22	42786007	C	T	+							passenger
chr22:g.50713847C>G	chr22	50713847	C	G	+		SHANK3	intron_variant	c.2114-1070C>G			driver

Table S8. Annotation of putative somatic SNVs from the TNBC sample measured in single-cell DNA profile.

Supplementary Note 1: Monopogen implementation

a

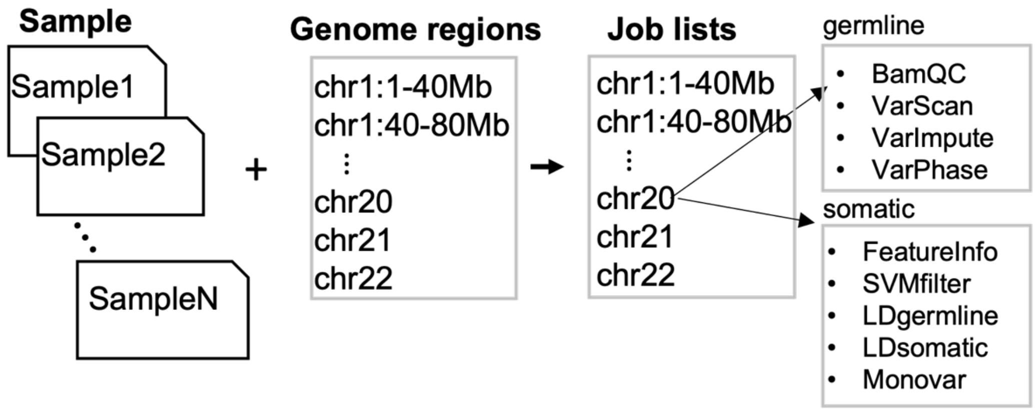

b

SNV caller	version	No. of CPUs	Wall clock time	Peak memory
Monopogen	V1.0	8	24 mins	49.6GB
Samtools	V1.2	1	43 mins	103Mb
GATK	V4.2.6.1	8	180 mins	68.4GB
FreeBayes	V1.3.6	1	10 mins	84Mb
Strelka2	V2.9.10	8	8 mins	664Mb
scAllele	V0.0.9.3	8	36 mins	24.4GB
cellSNP	V0.3.2	8	128 mins	4.4GB

Implementations of Monopogen on SNV calling in single cell sequencing data. a, Monopogen adopts the framework implemented in GotCloud for Trans-Omics for Precision Medicine Program (TOPMed), by splitting the genome into small chunks (defined by the users), performing variant scan and LD refinement in massive parallelization for individual chunks and merging the results. **b**, Wall clock time and memory usage for 7 SNV callers on germline SNV calling. We evaluated computational usages using single cell DNA sequencing reads from chr20 of TNBC sample (TN28). Samtools and FreeBayes do not support multi-threading feature. For other tools, the peak

memory includes memory usage from all CPUs. The tests were performed on a high-performance computing cluster with Intel Xeon CPUs (2.30 GHz).
