# Supplementary material for: Single-nucleotide variant calling in single-cell sequencing data with Monopogen
Source: Nat Biotechnol. 2023 Aug 17;42(5):803–12. doi: 10.1038/s41587-023-01873-x (PMC11098741; doi:10.1038/s41587-023-01873-x)
Supplement: Supplementary file 2 — Reporting Summary [file 41587_2023_1873_MOESM2_ESM.pdf]

nature portfolio

Corresponding author(s): Ken Chen

Last updated by author(s): May 26, 2023

Reporting Summary

Nature Portfolio wishes to improve the reproducibility of the work that we publish. This form provides structure for consistency and transparency in reporting. For further information on Nature Portfolio policies, see our Editorial Policies and the Editorial Policy Checklist.

Statistics

For all statistical analyses, confirm that the following items are present in the figure legend, table legend, main text, or Methods section.

n/a	Confirmed	
☐	☒	The exact sample size (*n*) for each experimental group/condition, given as a discrete number and unit of measurement
☐	☒	A statement on whether measurements were taken from distinct samples or whether the same sample was measured repeatedly
☐	☒	The statistical test(s) used AND whether they are one- or two-sided<br>*Only common tests should be described solely by name; describe more complex techniques in the Methods section.*
☐	☒	A description of all covariates tested
☐	☒	A description of any assumptions or corrections, such as tests of normality and adjustment for multiple comparisons
☐	☒	A full description of the statistical parameters including central tendency (e.g. means) or other basic estimates (e.g. regression coefficient) AND variation (e.g. standard deviation) or associated estimates of uncertainty (e.g. confidence intervals)
☐	☒	For null hypothesis testing, the test statistic (e.g. *F*, *t*, *r*) with confidence intervals, effect sizes, degrees of freedom and *P* value noted<br>*Give P values as exact values whenever suitable.*
☒	☐	For Bayesian analysis, information on the choice of priors and Markov chain Monte Carlo settings
☒	☐	For hierarchical and complex designs, identification of the appropriate level for tests and full reporting of outcomes
☐	☒	Estimates of effect sizes (e.g. Cohen's *d*, Pearson's *r*), indicating how they were calculated

Our web collection on statistics for biologists contains articles on many of the points above.

Software and code

Policy information about availability of computer code

Data collection	Our dataset is collected from public datasets or generated from our collaborators and hence have no specific softwares/tools used for collecting them.
Data analysis	Samtools (version 1.2); bcftools (version 1.8); GATK (version 4.2.6.1); RFMix (version 2); Beagle (version 4.1); Picard (version 2.274); GCTA (version 1.94.1); LASER (version 2.04); Seurat (version 4.01); FreeBayes (version1.3.6); Strelka2 (version 2.9.10); scAllele (version0.0.93), cellSNP (version 0.3.2); Mutec2 (included in GATK). R package qqman (version 0.1.8); plot_karyogram.py (https://github.com/armartin/ ancestry_pipeline/blob/master/plot_karyogram.py); R (version 3.6.1); Python (3.8.0);

For manuscripts utilizing custom algorithms or software that are central to the research but not yet described in published literature, software must be made available to editors and reviewers. We strongly encourage code deposition in a community repository (e.g. GitHub). See the Nature Portfolio guidelines for submitting code & software for further information.

Data

Policy information about availability of data

All manuscripts must include a data availability statement. This statement should provide the following information, where applicable:

- Accession codes, unique identifiers, or web links for publicly available datasets
- A description of any restrictions on data availability
- For clinical datasets or third party data, please ensure that the statement adheres to our policy

The sci-ATAC profiles from the two transverse colon samples were downloaded from ENCODE database at https://www.encodeproject.org/files/ENCFF354SCV/ and https://www.encodeproject.org/files/ENCFF491HQL/. The dataset is partly from ENCODE study [60].The matched VCF files for WGS genotypes were from accession https://www.encodeproject.org/files/ENCFF944WLM/ and https://www.encodeproject.org/files/ENCFF907ASL/.

The snRNA-seq and snATAC-seq profiles from the human heart left ventricle tissues of 65 donors were downloaded from ENCODE study [61] at https://www.encodeproject.org/matrix/?type=Experiment&assay_title=snATAC-seq&assay_title=scRNA-seq&biosample_ontology.term_name=heart+left+ventricle.

The 12 scRNA-seq samples with matched WGS genotypes were downloaded from GTEx database [61] with https://anvil.terra.bio/#workspaces/anvil-datastorage/AnVIL_GTEx_V9_hg38.

The 1KG3 genotypes were from 1000 genome project [62] and downloaded from https://ftp.1000genomes.ebi.ac.uk/vol1/ftp/data_collections/1000G_2504_high_coverage/working/20201028_3202_phased/.

The HGDP panel [63] genotypes were downloaded from http://csg.sph.umich.edu/chaolong/LASER/HGDP-938-632958.tar.gz.

The scDNA-seq from the TNBC sample was downloaded from breast cancer study [32].

The single-cell RNA of bone marrow sample used for somatic calling evalution was from MAESTER technology [33]. The fastq files were downloaded from SRA database with SRR15598778, SRR15598779, SRR15598780, SRR15598781, and SRR15598782. The integrated single-cell multi-omics profiles including gene expressions, mtDNA variant calls and TCR profiles were downloaded from https://vangalenlab.bwh.harvard.edu/resources/maester-2021/

The single cell profiles of 20 HBCA samples, 20 AIDA samples, and 4 retina samples were generated as part of the cell atlas and genetic ancestry networks organized by the Chan Zuckerberg Initiative. The 20 AIDA single-cell samples could be downloaded from https://data.humancellatlas.org/explore/projects/f0f89c14-7460-4bab-9d42-22228a91f185.
The 4 retina single-cell samples could be downloaded from https://data.humancellatlas.org/explore/projects/f0f89c14-7460-4bab-9d42-22228a91f185.
The 20 HBCA single-cell samples could be accessed through GSE195665 (https://navinlabcode.github.io/HumanBreastCellAtlas.github.io/dataAccess.html).

Human research participants

Policy information about studies involving human research participants and Sex and Gender in Research.

Reporting on sex and gender	Retina samples sex and gender information: 19D013-Female; 19D014-Male; 19D015-Male; 19D016-Male;<br>For heart left ventricle datasets from ENCODE, they are from public datasets with gender information in https://www.encodeproject.org/matrix/?type=Experiment&assay_title=snATAC-seq&assay_title=scRNA-seq&biosample_ontology.term_name=heart+left+ventricle.

Population characteristics	20 HBCA samples Caucasus;<br>20 AIDA samples:<br>JP_H045: Japanese<br>JP_H046: Japanese<br>JP_H047: Japanese<br>JP_H048: Japanese<br>JP_H137: Japanese<br>JP_H146: Japanese<br>JP_H148: Japanese<br>JP_H149: Japanese<br>KR_H001: Korean<br>KR_H002: Korean<br>KR_H004: Korean<br>KR_H005: Korean<br>KR_H160: Korean<br>KR_H161: Korean<br>KR_H164: Korean<br>KR_H165: Korean<br>Lonza_3038016: Unknown<br>Lonza_3038097: Unknown<br>Lonza_3038099: Unknown<br>Lonza_3038306: Unknown<br><br>Retina studies:<br>19D013: European<br>19D014: European<br>19D015: Hispanic<br>19D016: European<br><br>65 samples in heart left ventricle: Unknown. Identified using the software developed in this study.<br><br>2 colon single cell samples: Unknown<br><br>7 GTEx single cell samples: Unknown<br><br>1 TNBC sample: Unknown
Recruitment	N.A.
Ethics oversight	N.A.

Note that full information on the approval of the study protocol must also be provided in the manuscript.

Field-specific reporting

Please select the one below that is the best fit for your research. If you are not sure, read the appropriate sections before making your selection.

☒ Life sciences ☐ Behavioural & social sciences ☐ Ecological, evolutionary & environmental sciences

For a reference copy of the document with all sections, see nature.com/documents/nr-reporting-summary-flat.pdf

Life sciences study design

All studies must disclose on these points even when the disclosure is negative.

Sample size	HBCA cohort: 20 single cell samples; AIDA cohort: 20 single cell samples; ENCODE: 65 single cell samples; Retina cohort: 4 single cell samples. Colon single cell studies: 2 single cell samples; GTEx cohort: 7 single cell samples; TNBC study: sample size 1; Our study focused on SNV calling evaluation and each sample included over 100K SNVs. Thus one sample for each study is enough for SNV calling evaluation.
Data exclusions	No datasets were excluded
Replication	Each sample includes over 100K SNVs for SNV calling evaluation and replicates are not necessary
Randomization	Each sample includes over 100K SNVs for SNV calling evaluation and randomization of study samples is not necessary
Blinding	There is no clinical trial and blinding design is not necessary

Reporting for specific materials, systems and methods

We require information from authors about some types of materials, experimental systems and methods used in many studies. Here, indicate whether each material, system or method listed is relevant to your study. If you are not sure if a list item applies to your research, read the appropriate section before selecting a response.

Materials & experimental systems

n/a	Involved in the study
☒	☐ Antibodies
☒	☐ Eukaryotic cell lines
☒	☐ Palaeontology and archaeology
☒	☐ Animals and other organisms
☒	☐ Clinical data
☒	☐ Dual use research of concern

Methods

n/a	Involved in the study
☒	☐ ChIP-seq
☒	☐ Flow cytometry
☒	☐ MRI-based neuroimaging
